# The economic value of reducing mortality due to noncommunicable diseases and injuries

**DOI:** 10.1038/s41591-024-03248-4

**Published:** 2024-09-27

**Authors:** Stéphane Verguet, Sarah Bolongaita, Angela Y. Chang, Diego S. Cardoso, Gretchen A. Stevens

**Affiliations:** 1 Department of Global Health and Population, Harvard T.H. Chan School of Public Health, Boston, MA USA; 2 Bergen Centre for Ethics and Priority Setting, Department of Global Public Health and Primary Care, University of Bergen, Bergen, Norway; 3 Danish Institute for Advanced Study, University of Southern Denmark, Odense, Denmark; 4 Interdisciplinary Centre on Population Dynamics (CPop), University of Southern Denmark, Odense, Denmark; 5 Department of Agricultural and Consumer Economics, University of Illinois Urbana-Champaign, Urbana, IL USA; 6 Independent researcher, Los Angeles, CA USA

**Keywords:** Health care, Health care economics

## Abstract

With population aging, national health systems face difficult trade-offs in allocating resources. The World Bank launched the Healthy Longevity Initiative to generate evidence for investing in policies that can improve healthy longevity and human capital. As part of this initiative, we quantified the economic value of reducing avoidable mortality from major noncommunicable diseases and injuries. We estimated avoidable mortality—the difference between lowest-achieved mortality frontiers and projected mortality trajectories—for each cause of death, for 2000, 2019 and 2050, and for geographic regions, with high-income countries, India and China considered separately; we applied economic values to these estimates. The economic value of reducing cardiovascular disease avoidable mortality would be large for both sexes in all regions, reaching 2–8% of annual income in 2019. For cancers, it would be 5–6% of annual income in high-income countries and China, and for injuries, it would be around 5% in sub-Saharan Africa and Latin America and the Caribbean. Despite the large uncertainty surrounding our estimates, we offer economic values for reducing avoidable mortality by cause and metrics comparable to annual incomes, which enable multisectoral priority setting and are relevant for high-level policy discussions around budget and resource allocations.

## Abstract (web summary)

This study reports substantial economic value in annual income if mortality due to cardiovascular diseases, cancers and injuries is reduced.

## Main

Before the onset of the COVID pandemic in 2020, life expectancy at birth and longevity were regularly progressing^1,2^. In many countries, the proportion of older adults is increasing as a result of major declines in fertility and important progresses in longevity^3^. Such rapid transitions can be accompanied by either the expansion or compression of morbidity^4–6^. This imposes new and substantial challenges to health systems, necessitating advanced technologies, the implementation of innovative delivery platforms and the strengthening of financial architectures^7–9^. Facing limited resources and an ever-increasing burden of noncommunicable diseases (NCDs), including cardiovascular diseases (CVDs; for example, stroke, ischemic heart disease) and malignant neoplasms (for example, cancers), now leading causes of death in a large number of countries globally^10,11^, at times accompanied by large burdens of injuries (either intentional or unintentional) in specific geographic regions, national health systems face difficult trade-offs when allocating resources toward improving the healthy longevity of their populations.

In this context, the World Bank designed the Healthy Longevity Initiative (HLI) to develop an evidence base for life-course investments in policies that can improve healthy longevity and human capital. The HLI provides new tools for decision-makers to launch healthy longevity programs tailored to national priorities^12,13^. Estimating the economic value of healthy longevity, via reducing avoidable mortality from major causes of death including NCDs, provides the foundations toward evaluating the potential longevity benefits of the prevention and control policies under consideration by the HLI. A money-metric value of reducing avoidable mortality by cause of death can directly speak to decision-makers beyond ministries of health, those in other sectors and ministers of finance.

Researchers use a variety of analytical methods for generating evidence to inform decision-making on which health interventions should or should not be prioritized. Common approaches include reporting on the mortality and morbidity outcomes of diseases. However, using such non-monetary valuations of health outcomes can prevent comparisons with assessments outside of the health sector (for example, education and social protection). As such, they are less amenable to comparison and decision for those making intersectoral allocations such as ministers of finance.

One approach that can address this intersectoral comparison issue is the use of value per statistical life (VSL)^14,15^, the value that one places on small reductions in mortality risk, where mortality outcomes are expressed in monetary terms. The Copenhagen Consensus Center^16^ has long used monetized health outcomes to compare the impact of health interventions with a variety of policies globally across key human capital and development sectors (for example, education, agriculture) in low- and middle-income countries (LMICs)^17–19^. Likewise, a seminal study that estimated the economic gains from reducing under-five mortality in LMICs also showed the utility of monetizing health outcomes^20^. Such methods were then replicated to assess the economic value of changing mortality risks associated with NCDs^21^, pointing to the vast welfare losses associated with elevated NCD mortality.

Therefore, transforming longevity measures into monetary values will prove useful when policymakers intend to set national priorities and to compare mortality reduction benefits with other kinds of benefits emanating from different sectors, beyond the health sector. While one will also need the implementation costs of health interventions when allocating resources, our paper focuses on estimating the economic value of reducing avoidable mortality caused by NCD or injury, a step toward quantifying a subset (associated with mortality only) of the extent of benefits associated with health improvements in monetary terms.

Our assessment is pursued for six world regions, three geographic regions (Eurasia and the Mediterranean, Latin America and the Caribbean, sub-Saharan Africa), with India, China and high-income countries considered separately, and 31 major disease and injury areas (for example, cancers, CVD). Our objective is to provide a systematic assessment of the economic value associated with reducing mortality by cause. In doing so, we derive a metric comparable to annual incomes (that is, gross national income (GNI)), which also can be interpreted as the percentage of income an individual would be willing to forgo to live 1 year under the lowest possible mortality rate for a given cause of death. The main findings and policy implications of the work are summarized in Table 1.

**Table 1:** Policy summary

Background	• With population aging and the increasing burden of NCDs, national health systems face difficult trade-offs in allocating resources across disease control priorities and compete for securing finances outside of the health sector.
	• We quantified avoidable mortality—the difference between lowest-achieved mortality and projected mortality trajectories—from major disease and injury causes of death, to which, using standard economic methods, we assigned a monetary value for 2000, 2019 and 2050, for geographic regions, with high-income countries, China and India separated.
Main findings and limitations	• The economic value associated with CVD avoidable mortality is large for females and males in all regions, spanning 2–8% of annual income in 2019. For cancers, it would represent 5–6% of annual income for both males and females in high-income countries and China. For injuries, it would be substantial in Latin America and the Caribbean and sub-Saharan Africa (5% of annual income).
	• While we applied standard economic methods and best practices, and used the best available data, important limitations include cause-specific mortality estimates based on incomplete data, especially in low- and middle-income countries, and absence of cost considerations of health interventions, which are needed to fully assess the returns on investment from NCD and injury control, including the political constraints associated with achieving mortality frontiers in many countries.
	• We also must stress that our estimates of the economic value of reducing avoidable mortality are surrounded by large uncertainty that is difficult to quantitatively ascertain.
Policy implications	• The economic implications of controlling NCD and injury mortality would be substantial, particularly for CVD, cancers and injuries. This highlights the urgency of scaling up largely underused cost-effective preventive, curative and public health interventions in all countries.
	• We derive an economic metric of avoidable mortality directly comparable to annual incomes, which enables intersectoral priority setting and can directly speak to ministers of finance in showing the large value associated with longevity and health.
	• This analysis of the economic value of reducing avoidable mortality by cause of death would be best interpreted and used as evidence in high-level policy discussions.

## Results

We first report on the estimation of avoidable mortality by cause of death and region. We then show the economic values associated with reducing avoidable mortality per cause and region. We used and condensed the World Health Organization (WHO) comprehensive list of disease and injury causes of death to a smaller list of mutually exclusive, collectively exhaustive set of causes primarily focused on NCDs and injuries (Table 2).

**Table 2:** Causes of death used in the study, adapted from the WHO Global Health Estimates (GHE)^10^

Level	Cause of death
1	**Communicable, maternal, perinatal and nutritional conditions**
2	Infectious and parasitic diseases
2	Maternal and neonatal conditions
2	Nutritional deficiencies
1	**NCDs**
2	CVDs
3	Ischemic heart disease
3	Stroke
3	Other CVDs
2	Diabetes mellitus
2	Digestive diseases
3	Cirrhosis of the liver
3	Other digestive diseases
2	Malignant neoplasms
3	Breast cancer
3	Cervix uteri cancer
3	Liver cancer
3	Mouth and oropharynx cancers
3	Esophagus cancer
3	Stomach cancer
3	Trachea, bronchus and lung cancers
3	Other malignant neoplasms
2	Respiratory diseases
3	Chronic obstructive pulmonary disease
3	Other respiratory diseases
2	Other NCDs
1	**Injuries**
2	Intentional injuries
2	Unintentional injuries
3	Road injury
3	Other unintentional injuries

Level 1 and 2 causes of death are mutually exclusive and collectively exhaustive; within level 2 causes of death, level 3 causes are mutually exclusive and collectively exhaustive. Supplementary Table 2 gives a mapping between GHE causes of death and the causes of death used in the analysis.

### Frontiers and region-specific mortality trajectories

**Fig. 1:**
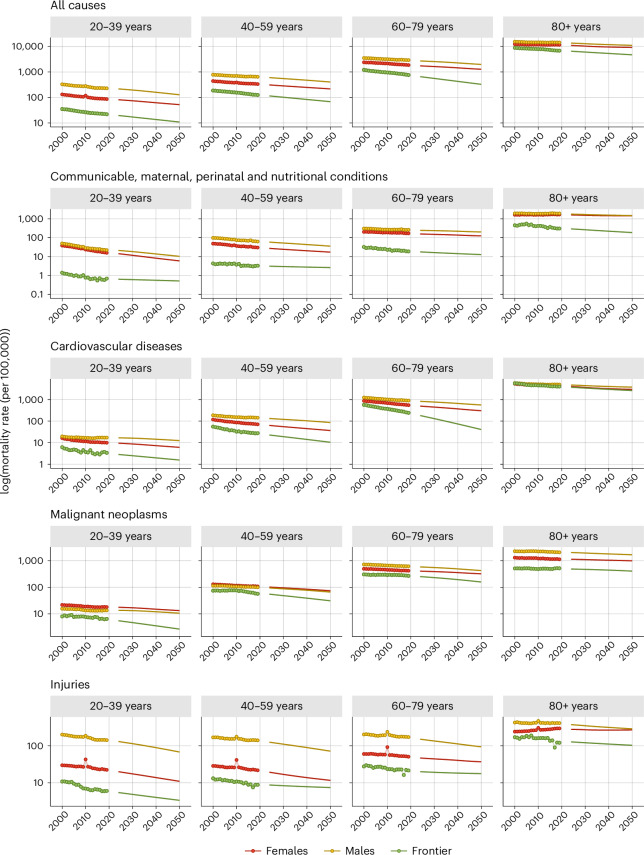
Mortality trajectories in Latin America and the Caribbean compared to the mortality frontier. Mortality trajectories in Latin America and the Caribbean and the mortality frontier for the period 2000–2050. By sex, age group (20–39 years, 40–59 years, 60–79 years and 80+ years) and cause of death (all causes; communicable, maternal, perinatal and nutritional conditions; cardiovascular diseases; malignant neoplasms; and injuries). Points correspond to WHO Global Health Estimates^10^ for the period 2000–2019; the lines are projections. The impact of COVID is not accounted for in these projections; therefore, 2020–2023 is not shown. Some of the slight fluctuations observed for the frontiers are due to scaling with all-cause frontiers estimated in ref. ^22^. For the mortality trajectories, the fluctuations observed are due to mortality shocks such as the Haiti earthquake in 2010, the method used for calculating regional estimates (aggregation of population-weighted country-specific estimates) and the scaling with the all-cause trajectory.

Figure 1 shows the estimated mortality frontiers (for 2000–2050) for ages 20 years and older (the principal age groups over which NCDs manifest) for the following broad causes of death: all causes; communicable, maternal, perinatal and nutritional conditions; CVD; malignant neoplasms (cancers); and injuries (Fig. 1). As expected, the mortality frontiers are higher (higher mortality rates) for older-age groups.

**Extended Data Fig. 1:**
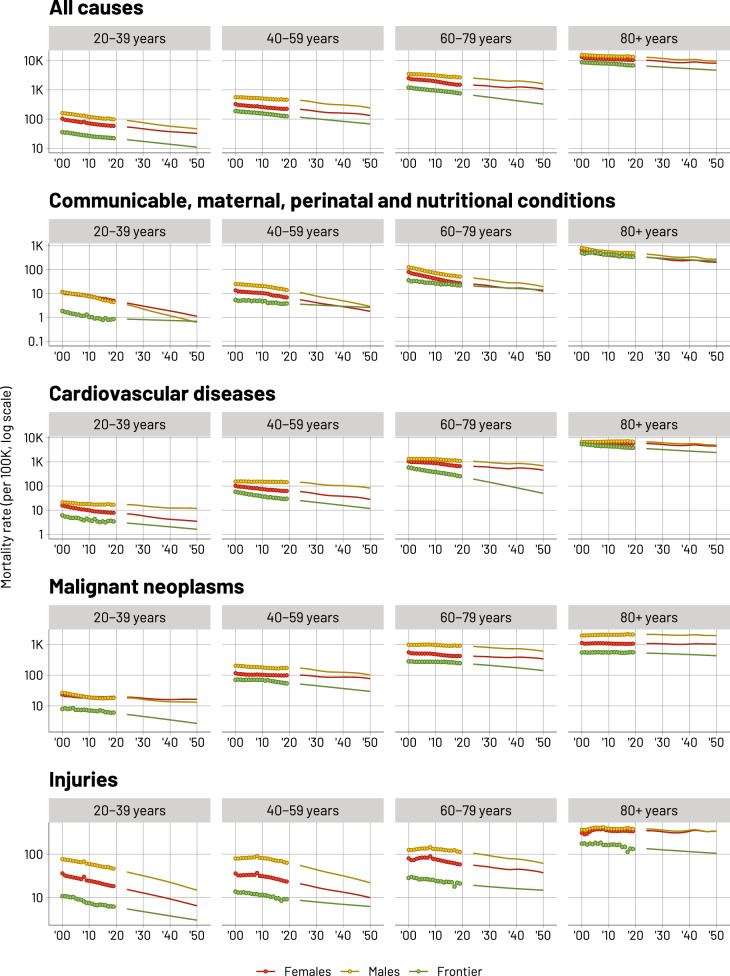
Mortality trajectories in China and the mortality frontier for the period 2000–2050. By sex, age group (20–39 years, 40–59 years, 60–79 years, 80+ years), and cause of death (all causes; communicable, maternal, perinatal and nutritional conditions; cardiovascular diseases; malignant neoplasms; and injuries). Points correspond to WHO’s Global Health Estimates^10^ for the period 2000–2019; the lines are projections. The impact of COVID is not accounted for in these projections and therefore years 2020–2023 are not displayed. Some of the slight fluctuations observed for the frontiers are due to scaling with all-cause frontiers estimated in ref. ^22^. For the mortality trajectories, the fluctuations observed are due to mortality shocks such as the Haiti earthquake in 2010, the method used for calculating regional estimates (aggregation of population-weighted country-specific estimates) as well as the scaling with the all-cause trajectory.

**Extended Data Fig. 2:**
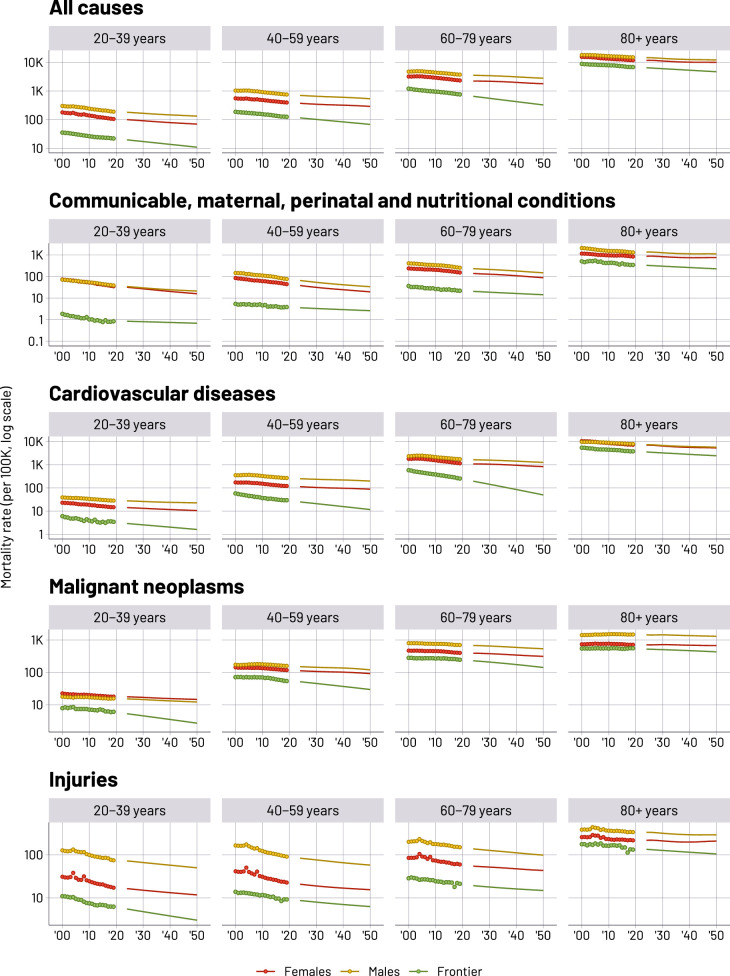
Mortality trajectories in Eurasia & the Mediterranean and the mortality frontier for the period 2000–2050. By sex, age group (20–39 years, 40–59 years, 60–79 years, 80+ years), and cause of death (all causes; communicable, maternal, perinatal and nutritional conditions; cardiovascular diseases; malignant neoplasms; and injuries). Points correspond to WHO’s Global Health Estimates^10^ for the period 2000–2019; the lines are projections. The impact of COVID is not accounted for in these projections and therefore years 2020–2023 are not displayed. Some of the slight fluctuations observed for the frontiers are due to scaling with all-cause frontiers estimated in ref. ^22^. For the mortality trajectories, the fluctuations observed are due to mortality shocks such as the Haiti earthquake in 2010, the method used for calculating regional estimates (aggregation of population-weighted country-specific estimates) as well as the scaling with the all-cause trajectory.

**Extended Data Fig. 3:**
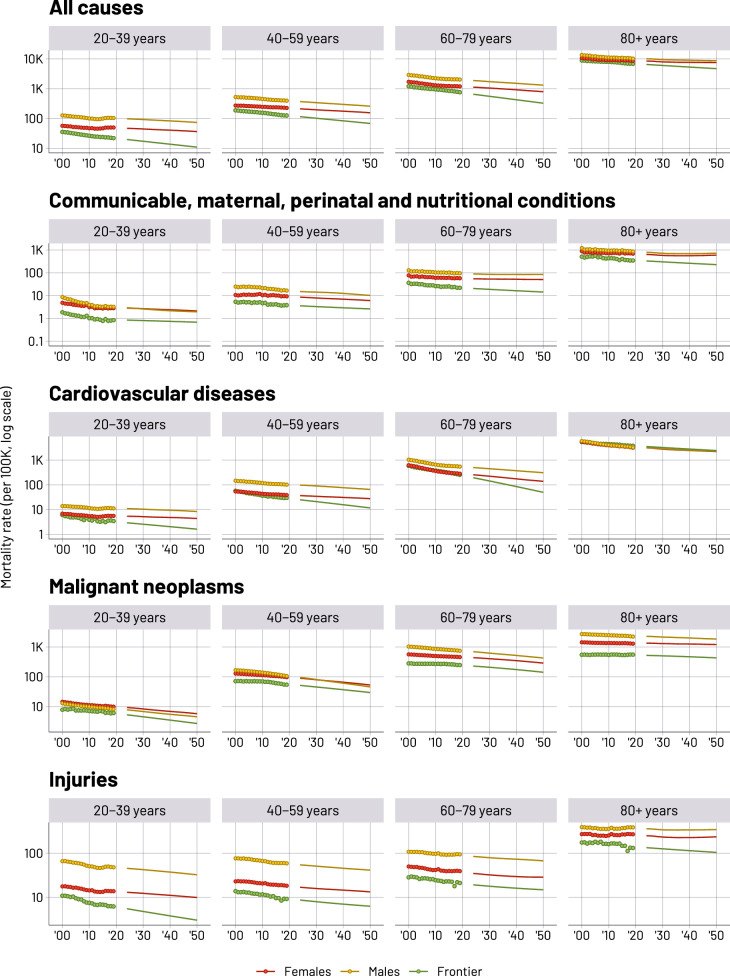
Mortality trajectories in High-Income and the mortality frontier for the period 2000–2050. By sex, age group (20–39 years, 40–59 years, 60–79 years, 80+ years), and cause of death (all causes; communicable, maternal, perinatal and nutritional conditions; cardiovascular diseases; malignant neoplasms; and injuries). Points correspond to WHO’s Global Health Estimates^10^ for the period 2000–2019; the lines are projections. The impact of COVID is not accounted for in these projections and therefore years 2020–2023 are not displayed. Some of the slight fluctuations observed for the frontiers are due to scaling with all-cause frontiers estimated in ref. ^22^. For the mortality trajectories, the fluctuations observed are due to mortality shocks such as the Haiti earthquake in 2010, the method used for calculating regional estimates (aggregation of population-weighted country-specific estimates) as well as the scaling with the all-cause trajectory.

**Extended Data Fig. 4:**
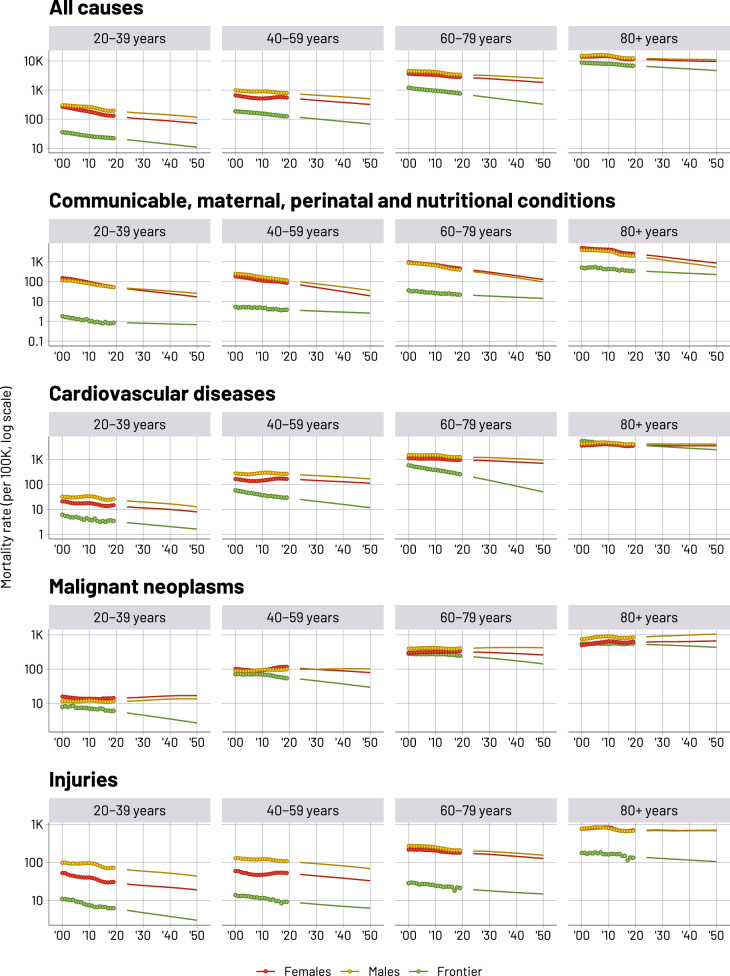
Mortality trajectories in India and the mortality frontier for the period 2000–2050. By sex, age group (20–39 years, 40–59 years, 60–79 years, 80+ years), and cause of death (all causes; communicable, maternal, perinatal and nutritional conditions; cardiovascular diseases; malignant neoplasms; and injuries). Points correspond to WHO’s Global Health Estimates^10^ for the period 2000–2019; the lines are projections. The impact of COVID is not accounted for in these projections and therefore years 2020–2023 are not displayed. Some of the slight fluctuations observed for the frontiers are due to scaling with all-cause frontiers estimated in ref. ^22^. For the mortality trajectories, the fluctuations observed are due to mortality shocks such as the Haiti earthquake in 2010, the method used for calculating regional estimates (aggregation of population-weighted country-specific estimates) as well as the scaling with the all-cause trajectory.

**Extended Data Fig. 5:**
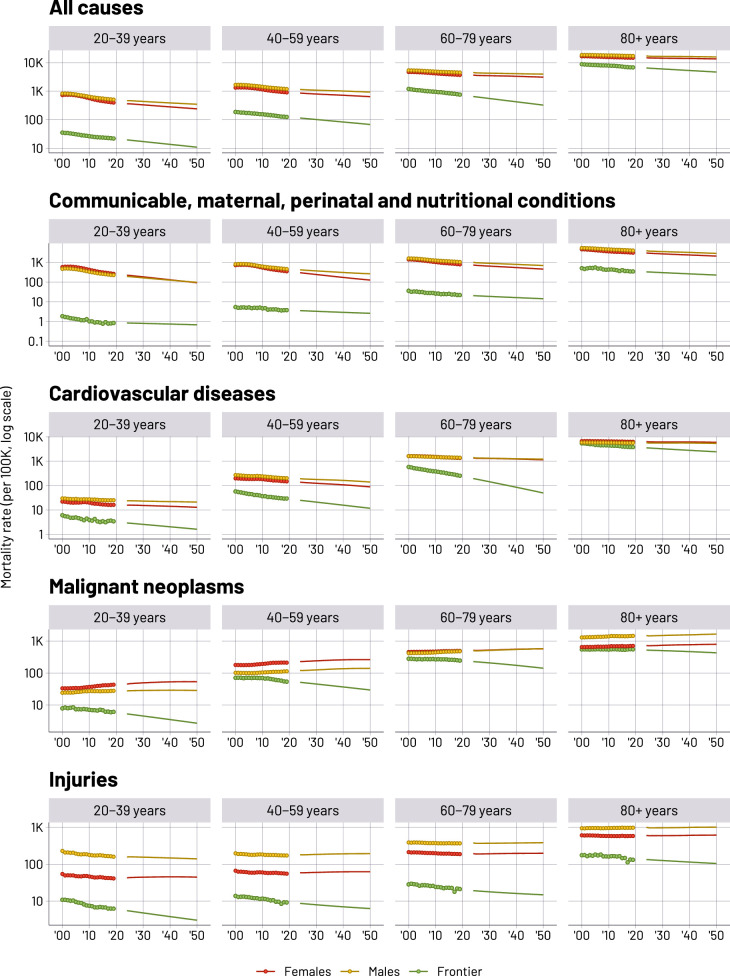
Mortality trajectories in sub-Saharan Africa and the mortality frontier for the period 2000–2050. By sex, age group (20–39 years, 40–59 years, 60–79 years, 80+ years), and cause of death (all causes; communicable, maternal, perinatal and nutritional conditions; cardiovascular diseases; malignant neoplasms; and injuries). Points correspond to WHO’s Global Health Estimates^10^ for the period 2000–2019; the lines are projections. The impact of COVID is not accounted for in these projections and therefore years 2020–2023 are not displayed. Some of the slight fluctuations observed for the frontiers are due to scaling with all-cause frontiers estimated in ref. ^22^. For the mortality trajectories, the fluctuations observed are due to mortality shocks such as the Haiti earthquake in 2010, the method used for calculating regional estimates (aggregation of population-weighted country-specific estimates) as well as the scaling with the all-cause trajectory.

**Extended Data Fig. 6:**
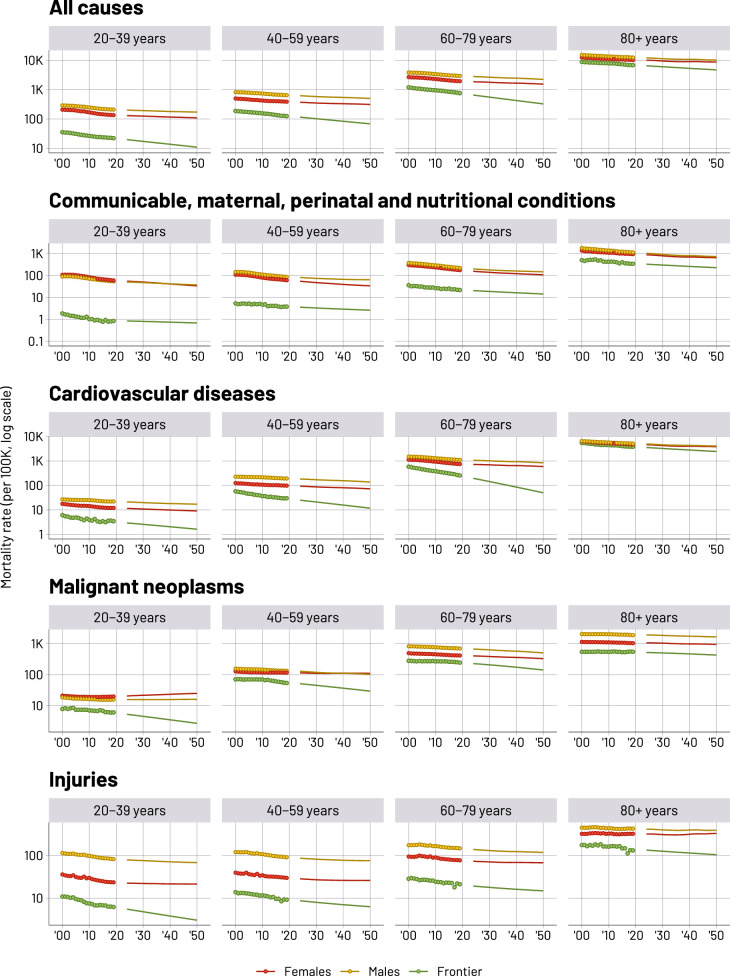
Mortality trajectories for the World and the mortality frontier for the period 2000–2050. By sex, age group (20–39 years, 40–59 years, 60–79 years, 80+ years), and cause of death (all causes; communicable, maternal, perinatal and nutritional conditions; cardiovascular diseases; malignant neoplasms; and injuries). Points correspond to WHO’s Global Health Estimates^10^ for the period 2000–2019; the lines are projections. The impact of COVID is not accounted for in these projections and therefore years 2020–2023 are not displayed. Some of the slight fluctuations observed for the frontiers are due to scaling with all-cause frontiers estimated in ref. ^22^. For the mortality trajectories, the fluctuations observed are due to mortality shocks such as the Haiti earthquake in 2010, the method used for calculating regional estimates (aggregation of population-weighted country-specific estimates) as well as the scaling with the all-cause trajectory.

Mortality trajectories for the six regions were estimated by sex for all age groups and causes (over 2000–2050). As an illustration, we show selected trajectories for the Latin America and the Caribbean region (Fig. 1); all other regional groupings are shown in Extended Data Figs. 1–6. As expected, the trajectories show higher mortality rates for the older-age groups for all causes; in general, the trajectories are also higher for males than for females. All estimated frontiers for all causes along with mortality trajectories for all regions and causes are available online (‘Data Availability’).

### Economic value of reducing avoidable mortality

**Fig. 2:**
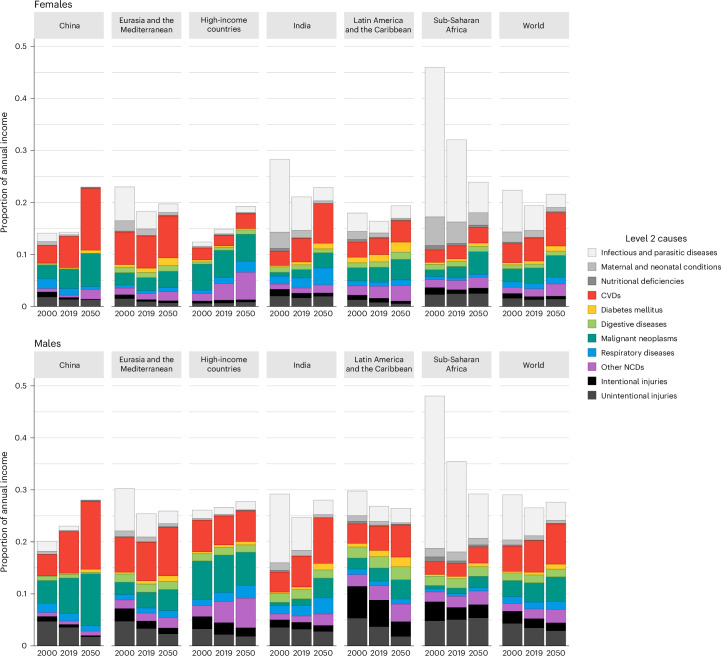
Economic value of reducing avoidable mortality. Economic values of reducing avoidable mortality, measured as proportion of annual income, assigned to selected causes of death (level 2 causes of death) for the six regions and the world, in 2000, 2019 and 2050; females and males.

For each region and the years 2000, 2019 and 2050, we show the economic valuations (summed across all age groups) per cause (Fig. 2 and Supplementary Table 4). Alternative disaggregations (according to level 1 and 3 causes) along with relative distributions of these economic valuations are shown in Supplementary Information 2 (section 1).

We observe substantial variations across regions, as well as some slight differences when comparing females and males. First, CVD contributes a very substantial economic value. For example, in 2019, the economic value of reducing avoidable CVD mortality amounted to around 6.8% of annual income in Eurasia and the Mediterranean (6.1% for females, 7.4% for males), 7.0% in China (5.9% for females, 8.0% for males), 5.2% in India (4.5% for females, 5.9% for males), 3.9% in Latin America and the Caribbean (3.1% for females, 4.6% for males), 3.7% in high-income countries (2.0% for females, 5.5% for males) and 2.5% in sub-Saharan Africa (2.5% for females, 2.5% for males).

Second, the economic value of reducing avoidable cancer (malignant neoplasms) mortality was also large, yet it was more geographically concentrated. In 2019, the corresponding economic value was approximately 6.2% of annual income in high-income countries (5.2% for females, 7.2% for males) and 5.3% in China (3.8% for females, 6.8% for males). These economic values were smaller in Eurasia and the Mediterranean with 2.8% of annual income in 2019 (2.5% for females, 3.1% for males), in India with 1.4% (1.6% for females, 1.3% for males), in Latin America and the Caribbean with 2.7% (2.9% for females, 2.5% for males) and in sub-Saharan Africa with 1.7% (2.2% for females, 1.1% for males).

Third, the economic value of reducing avoidable injury mortality varied geographically and by sex. The economic values were substantial for males in Latin America and the Caribbean, with around 8.8% of annual income in 2019 (compared with 1.6% for females) and a concentration within intentional injuries (5.1% of annual income for males). They were also large for males in sub-Saharan Africa, with approximately 7.4% of annual income in 2019 (compared with 3.2% for females) and a concentration within unintentional injuries (5.1% of annual income for males). On the contrary, for the other four regions, the economic values of reducing avoidable injury mortality were lower: between 4.1% and 4.8% of annual income for males, between 1.3% and 2.6% for females, in the year 2019.

Fourth, we note that the economic value associated with the cause category ‘communicable, maternal, perinatal and nutritional conditions’ was very substantial in sub-Saharan Africa, amounting to around 20.0% of annual income in 2019 (20.4% for females, 19.6% for males), and it was also large in India with about 7.8% of annual income in 2019 (8.1% for females, 7.5% for males). This is due to high mortality rates for these causes and to large young populations, especially in sub-Saharan Africa^1^. The economic values of reducing avoidable mortality for this cause category were, however, much smaller in the other four regions. This was particularly so in high-income countries with around 1.6% of annual income in 2019 (1.5% for females, 1.8% for males) and China with about 0.9% of annual income in 2019 (0.8% for females, 1.1% for males), two geographical locations that had the lowest mortality from communicable, maternal, perinatal and nutritional conditions.

**Fig. 3:**
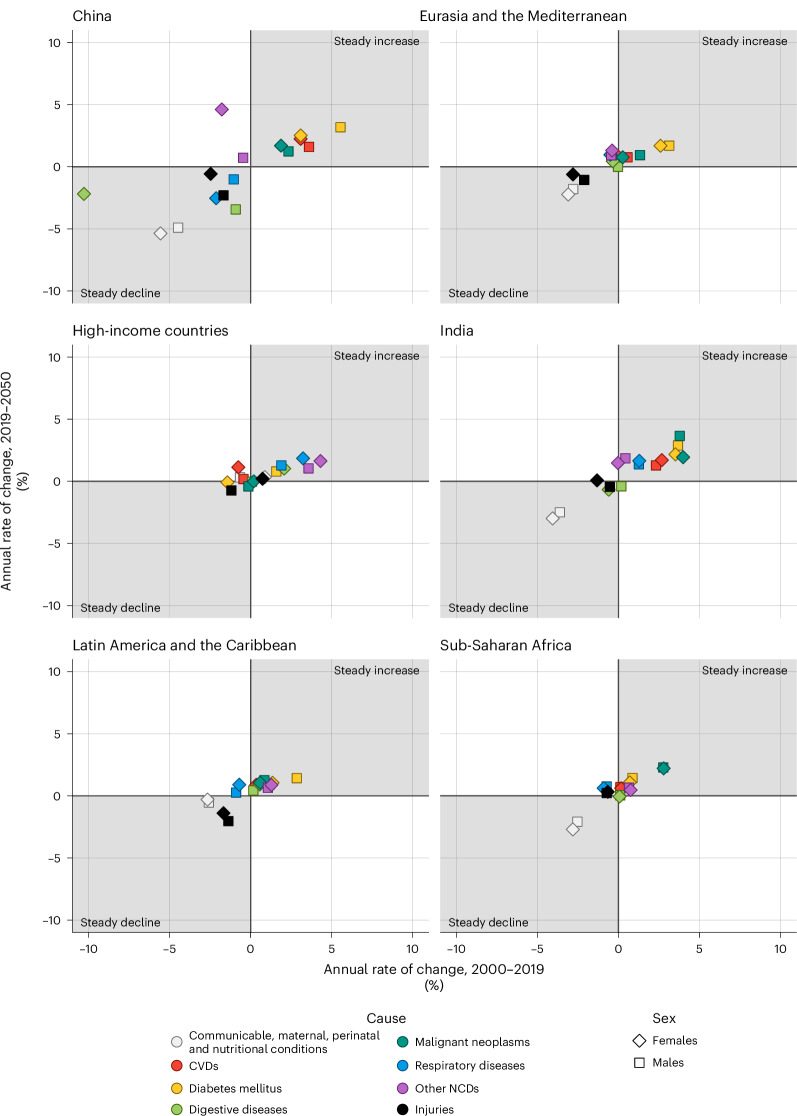
Rates of change of the economic value of reducing avoidable mortality. Rates of change (percentage per year) of the economic values of reducing avoidable mortality assigned to selected causes of death (communicable, maternal, perinatal and nutritional conditions; cardiovascular diseases (CVDs); diabetes mellitus; digestive diseases; malignant neoplasms; respiratory diseases; other noncommunicable diseases (NCDs); and injuries) for the six regions, over the time periods 2000–2019 and 2019–2050; females and males.

Lastly, in almost all regions, we could point to the rapid expansion of avoidable deaths associated with NCDs, in particular CVDs and cancers, and to the fast compression of avoidable mortality from communicable, perinatal, maternal and nutritional conditions, into the future (from 2000 to 2019 and 2050; Fig. 3). This is a testimony of the epidemiological transition from communicable to noncommunicable diseases and aging. Over 2000–2050, we observed steady declines in the economic value associated with reducing avoidable mortality from communicable, perinatal, maternal and nutritional conditions coupled with steady expansions of the economic value of reducing avoidable mortality from CVD (except for sub-Saharan Africa) and cancers in China, India and sub-Saharan Africa. For the other regions (for example, Eurasia and the Mediterranean, Latin America and the Caribbean, high-income countries), the extent of CVD and cancer expansions may be smaller, especially in high-income countries showing a rather stagnant picture. Overall, this dynamic evolution emphasizes the paramount challenges and economic losses expected from increasing numbers of CVD and cancer deaths, replacing communicable, perinatal, maternal and nutritional conditions, particularly in the emerging economies of China, India and sub-Saharan Africa.

### Uncertainty and sensitivity analyses

First and foremost, we must highlight that there are major structural and parameter uncertainties accompanying the application of our approach and, thus, our estimations. Therefore, uncertainty around the estimates of economic value of reducing avoidable mortality we provide remains, to a large extent, unquantifiable.

**Extended Data Fig. 7:**
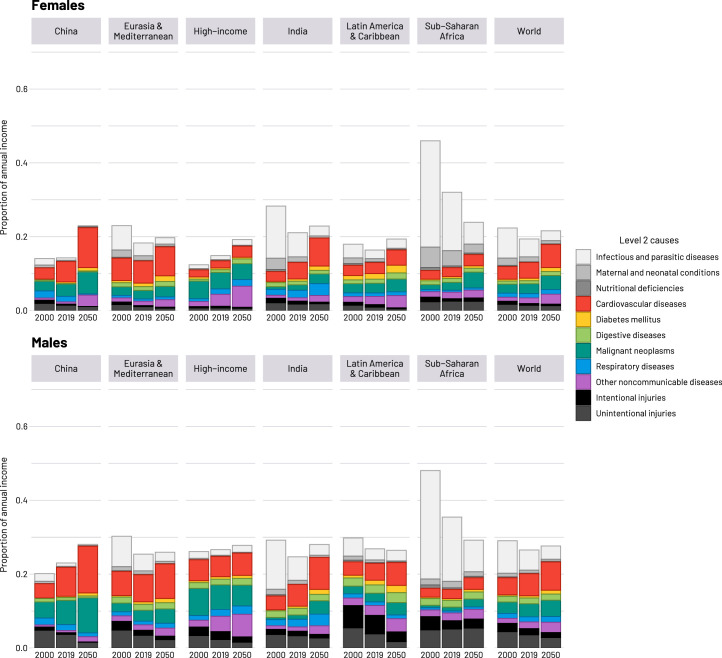
Economic values of reducing avoidable mortality: Using the minimum frontier definition. Economic values of reducing avoidable mortality, measured as percent of annual income, assigned to selected causes of death (level 2 causes of death) for the six regions and the world, in the years 2000, 2019, and 2050.

While we acknowledge that we do not fully know to what extent our economic value estimates are reliable, we proceeded to conducting numerous univariate sensitivity analyses. First, we implemented an alternative benchmark for the mortality frontiers, that is, the minimum mortality rates, instead of the 10th percentile of mortality rates (Extended Data Fig. 7), while maintaining the scaling to the frontiers previously estimated^22^. In this case, we observed only slight differences in the economic values of avoidable mortality estimated (Fig. 2 and Supplementary Table 4). For example, in 2019, the economic value of reducing avoidable CVD mortality would slightly change from 6.8% to 6.7% of annual income in Eurasia and the Mediterranean, from 7.0% to 6.7% in China and from 3.9% to 3.7% in Latin America and the Caribbean. There would be no change in India (5.2%), high-income countries (3.7%) and sub-Saharan Africa (2.5%).

**Fig. 4:**
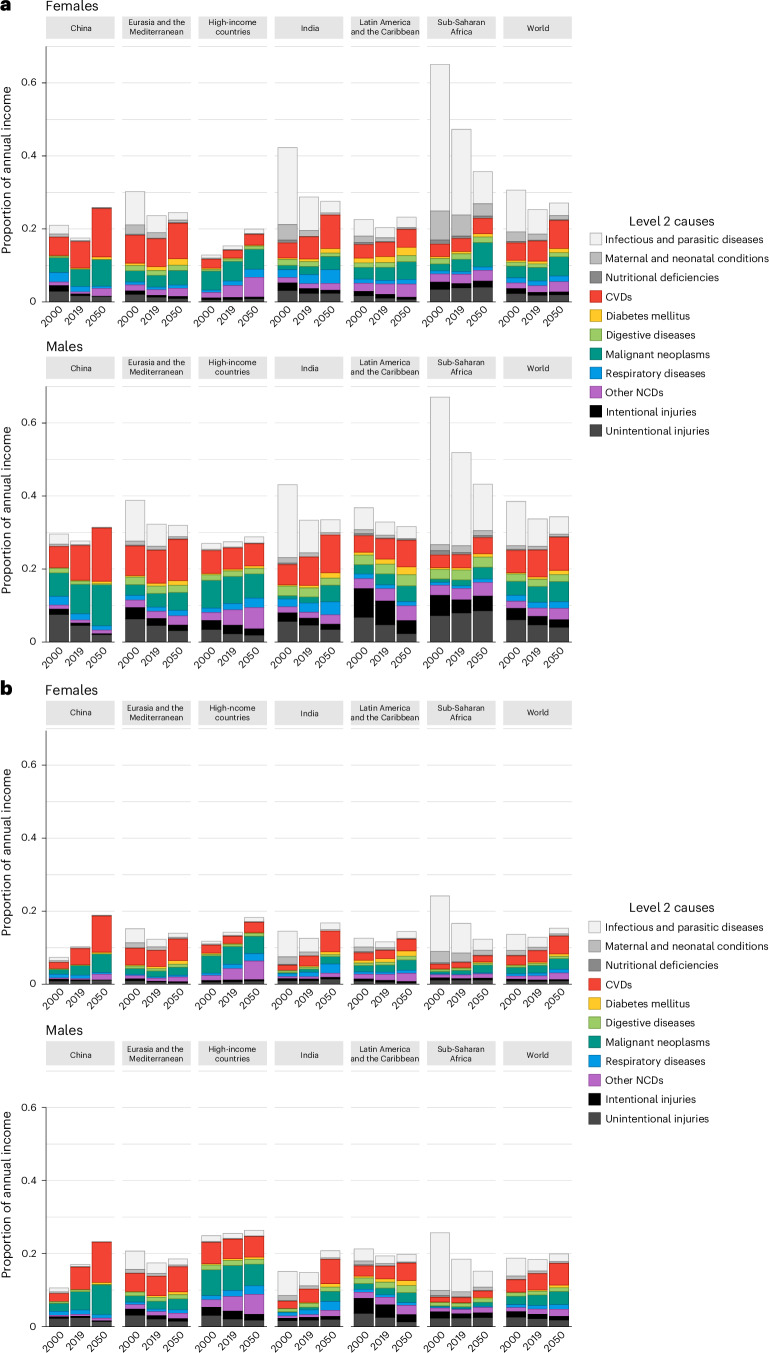
Economic value of reducing avoidable mortality based on sensitivity analyses. Economic values of reducing avoidable mortality, measured as the percentage of annual income, assigned to selected causes of death (level 2 causes of death) for the six regions and the world, in 2000, 2019 and 2050. **a**, Economic values of reducing avoidable mortality using a lower income elasticity of 1.0. **b**, Economic values of reducing avoidable mortality using a higher income elasticity of 1.5.

Second, we applied the full set of sensitivity analyses following best practices^14^. Firstly, we assigned different estimates of income elasticities (Fig. 4) for the VSL (the value that one places on reducing mortality risks). As expected, economic values for countries with lower income than the United States would then decrease with a higher elasticity and increase with a lower elasticity. For instance, in 2019, the economic value of reducing avoidable CVD mortality would change from 6.8% to either 4.8% (higher elasticity) or 8.3% (lower elasticity) of annual income in Eurasia and the Mediterranean, from 7.0% to either 5.2% (higher elasticity) or 8.3% (lower elasticity) in China, from 5.2% to either 3.1% (higher elasticity) or 6.9% (lower elasticity) in India, from 3.9% to either 2.8% (higher elasticity) or 4.7% (lower elasticity) in Latin America and the Caribbean, from 3.7% to either 3.6% (higher elasticity) or 3.8% (lower elasticity) in high-income countries and from 2.5% to either 1.3% (higher elasticity) or 3.5% (lower elasticity) in sub-Saharan Africa. However, in spite of these changes in quantitative estimates, we did not observe qualitatively distinct findings; that is, the relative distributions of the economic values of reducing avoidable mortality estimated would largely remain similar across disease categories.

**Extended Data Fig. 8:**
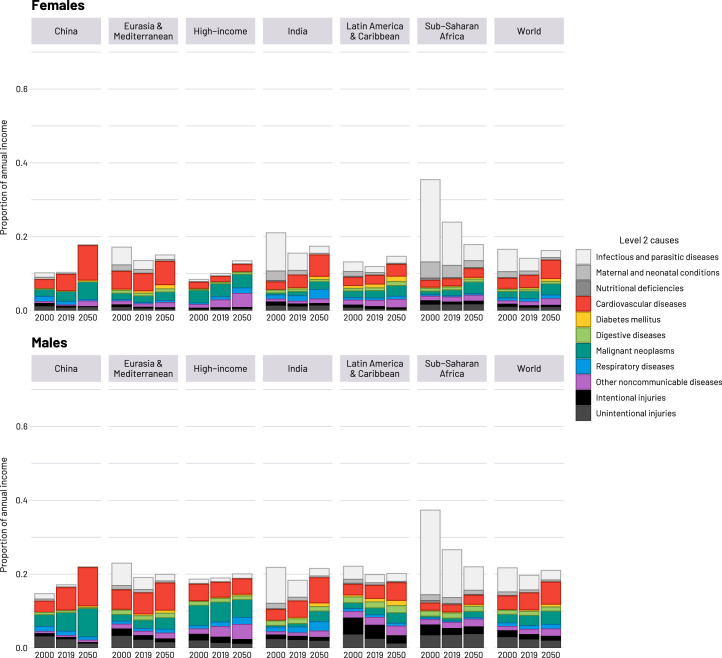
Economic values of reducing avoidable mortality: Using an alternative baseline value-per-statistical-life-to-income ratio of 100. Economic values of reducing avoidable mortality, measured as percent of annual income, assigned to selected causes of death (level 2 causes of death) for the six regions and the world, in the years 2000, 2019, and 2050.

Secondly, we set an alternative baseline for the initial VSL-to-income ratio (Extended Data Fig. 8). Here the estimated economic values would decrease in magnitude, but yet again, the relative distributions and rankings across causes of death would be maintained. For example, in 2019, the economic value of reducing avoidable CVD mortality would change from 6.8% to 5.2% of annual income in Eurasia and the Mediterranean, from 7.0% to 5.2% in China, from 5.2% to 3.9% in India, from 3.9% to 2.9% in Latin America and the Caribbean, from 3.7% to 2.6% in high-income countries and from 2.5% to 1.9% in sub-Saharan Africa.

**Extended Data Fig. 9:**
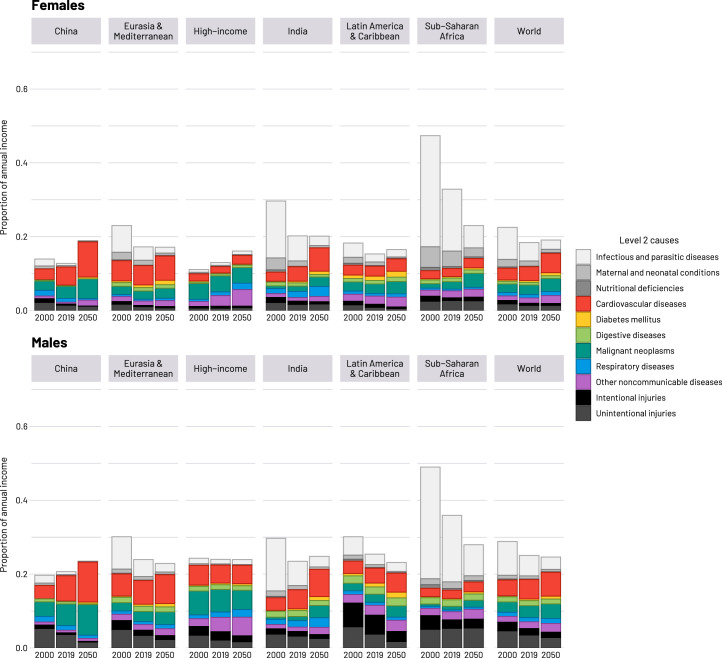
Economic values of reducing avoidable mortality: Using a lower discount rate of 1% per year. Economic values of reducing avoidable mortality, measured as percent of annual income, assigned to selected causes of death (level 2 causes of death) for the six regions and the world, in the years 2000, 2019, and 2050.

**Extended Data Fig. 10:**
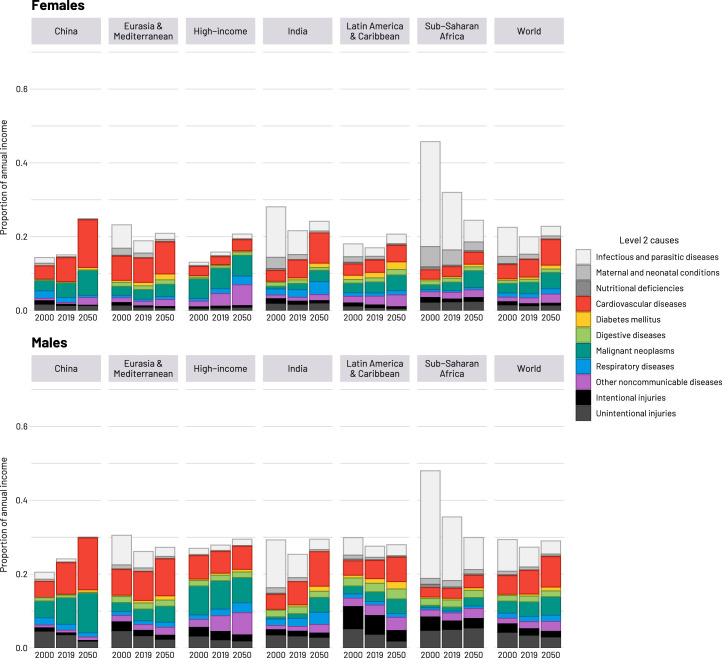
Economic values of reducing avoidable mortality: Using a higher discount rate of 5% per year. Economic values of reducing avoidable mortality, measured as percent of annual income, assigned to selected causes of death (level 2 causes of death) for the six regions and the world, in the years 2000, 2019, and 2050.

Thirdly, we applied alternative annual discount rates (1% and 5% per year; Extended Data Figs. 9 and 10). We then observed the following changes in the resulting economic values of reducing avoidable CVD mortality in 2019: from 6.8% to either 5.8% (1% discount rate) or 7.2% (5% discount rate) of annual income in Eurasia and the Mediterranean, from 7.0% to either 5.9% (1% discount rate) or 7.5% (5% discount rate) in China, from 5.2% to either 4.5% (1% discount rate) or 5.5% (5% discount rate) in India, from 3.9% to either 3.3% (1% discount rate) or 4.1% (5% discount rate) in Latin America and the Caribbean, from 3.7% to either 3.3% (1% discount rate) or 4.0% (5% discount rate) in high-income countries and from 2.5% to either 2.2% (1% discount rate) or 2.6% (5% discount rate) in sub-Saharan Africa.

## Discussion

We computed monetary values associated with reducing avoidable mortality for specific causes of death across geographic regions, high-income countries, and China and India. This economic framework provides a systematic assessment of the burden of avoidable mortality by cause. It can help quantify the benefits of health sector interventions and allow comparisons within the health sector as well as beyond across any sector, during high-level policy discussions around budget and resource allocations.

The findings indicate that the economic implications of controlling NCDs and injuries could be substantial and the costs of inaction would be enormous. We found that the economic value associated with reducing avoidable CVD mortality would be large in all regions, on the order of 2% to 8% of annual income in 2019. This is a testimony of the fact that CVD is a leading cause of death with the largest proportion of avoidable mortality almost everywhere globally^10^. We also observed that the economic value for cancers would be large in high-income countries (6% of annual income in 2019) and China (5%). This can be explained by the rapidly aging populations of Europe, North America and China^1,11^, combined with improvements in the control of major CVD risk factors such as hypertension in these regions^23,24^. As for injuries, the economic value would be substantial for males in Latin America and the Caribbean, essentially owing to intentional injuries (for example, homicides)^11,25^. It would also be large in sub-Saharan Africa, primarily as a result of unintentional injuries (for example, road traffic injuries)^11,25^. These substantial differences across regions and countries point to differential priorities in terms of the preventive, curative and public health interventions to be rolled out^26^.

The economic values estimated are very substantial and on the order of magnitude of current gross domestic expenditures in health in LMICs. The world’s current health expenditures amounted to 11% of the gross domestic product in 2020, with domestic general government health expenditures of 7% of the gross domestic product in 2020, ranging from around 1% and 3% in low-income and middle-income countries, respectively, to 9% in high-income countries^27^.

In computing these economic values, we assigned economic values for cause-specific mortality reductions and provided a metric (percentage of annual income) that is directly comparable to GNIs and annual budgetary allocations. In summary, our dual focus on avoidable mortality and monetary valuation of mortality risk reductions provides a priority-setting dimension complementary to traditional cause-of-death distributions (Supplementary Fig. 8). It also provides an economic metric directly comparable to annual incomes, which can provide direct evidence to ministers of finance and show them the enormous value of improving longevity and health.

Relatedly, a number of studies using similar economic approaches have been conducted. Bloom and colleagues have pioneered comparable methods and published seminal works to make the investment case for NCDs^28^, and they have also led macroeconomic modeling to assess the economic losses attributable to NCDs^29,30^. These studies, like ours, showed the very substantial welfare impact of NCDs. Khadka and Verguet^21^ developed approaches to quantify the monetary values associated with NCD excess mortality and estimated the corresponding welfare losses, particularly for CVD and cancers, under different feasible counterfactual scenarios in LMICs. Similarly, Arias and colleagues^31^, building on the work of Bloom and colleagues, pointed to the potentially largely undervalued economic losses tied to mental disorders globally. Beyond NCDs, comparable methods have been developed and applied, for example, for estimating the global costs of premature mortality from COVID^32,33^ or for quantifying the value of years of life lost due to mortality^34^. In summary, our analyses provide a systematic and comprehensive application to a large number of countries, causes of death and years, along with sophisticated estimation of mortality frontiers, rather than the development of intrinsically new methods. Of note, Da Costa et al.^35^ have also computed monetary values for the burden of disease (from both mortality and morbidity) in sub-Saharan Africa while assigning marginal values for extending the lives of older versus younger individuals, with direct implications for prioritizing NCDs versus communicable diseases.

Nevertheless, we fully acknowledge that our analysis has limitations. We used estimates of cause-specific mortality for countries worldwide, which are based on incomplete data, particularly in LMICs outside of Latin America^10^. Although we applied well-established and widely used methods for projecting mortality^36^, our future trends (over 2020–2050) in age–cause–sex-specific frontier mortality rates are based on previous trends using simple log-linear extrapolations. A more complex mortality forecasting approach could be pursued, yielding probabilistic distributions of a space of plausible future outcomes^37^. Yet, this would probably bring additional complications for interpretation without, however, being further grounded within empirical data. Also, we did not consider the short- or long-term impacts of COVID on age–cause-specific mortality rates in our forecasts, consistent with the UN World Population Prospects 2022^1^. In addition, in estimating mortality frontiers, we mostly used high-income country data because high-income countries would probably achieve the lowest mortality rates^10,11^. Alternative frontiers could be constructed (for example, region-specific frontiers instead of global ones). Relatedly, differences in cause-of-death patterns across geographies and time may relate to quality and practices in cause-of-death assignments versus real epidemiological differences, especially for related causes (for example, diabetes and CVD). Our methods are based on quantification of avoidable mortality, which by design excludes non-fatal conditions, such as sensory disorders (for example, vision and hearing impairments) and musculoskeletal and psychiatric disorders. As such, the exclusion of morbidity in the paper is a major limitation toward quantifying the full economic benefits of extended healthy living.

While we applied standard economic methods consistent with best practices^14^ and rigorously following our companion analysis^22^, there are major limitations with such approaches. There is high sensitivity of our VSL estimates to income elasticity parameters^15,38^, and we made a number of argued prescriptions regarding our parametrization, including specifying a modulating function varying with the extent of mortality risk reductions^22,32,39^. Therefore, we did not pursue probabilistic sensitivity analyses. Rather, we conducted recommended univariate sensitivity analyses, cognizant of the important structural uncertainties pertaining to our application of best practices^14^ (for example, the use of the US VSL as anchor). As an illustration, we tested alternative values of income elasticity and discount rates (Fig. 4 and Extended Data Figs. 8–10). In this respect, caution should be exercised when comparing the absolute economic values associated with reducing avoidable mortality across regions, even though we made an explicit effort to report our economic values in relative terms, that is, in percentage of annual incomes. Furthermore, to many, monetizing health outcomes raises ethical dilemmas. Often, VSL estimates are misunderstood as economic values society assigns to one life, rather than being understood as trade-offs individuals are willing to make in giving up a portion of income in exchange for small reductions in mortality risk^14^. Similarly, while avoidable mortality would probably vary substantially across socioeconomic groups (for example, income quintiles), within and across countries, we purposefully did not provide estimates across socioeconomic groups for ethical reasons. That being said, disaggregated valuations might be conducted, with the potential application of prioritarianism principles, that is, giving explicit priority to the worse off^40^.

We also computed monetary values associated with longevity losses, which only provides one element and does not include valuation of morbidity reduction and of potential non-health benefits (for example, financial protection). We did not estimate costs, including political and implementation challenges. This would enable full assessment of the value of disease and injury control, with, for example, the rollout of health sector and public health interventions^26,41^. We provide here only estimates for what would be mortality reduction benefits of hypothetical investments bringing countries to a low mortality frontier, even though, besides financial investments, political and implementation constraints could still be paramount. While costing the interventions to be rolled out was not our focus, even without intervention costs, reporting economic values in terms of percentage of income still yields metrics that can directly speak to ministries of finance making intersectoral allocations. Moreover, we do not state in this paper that achieving low mortality frontiers must be the goal for all countries. Rather, we use low mortality frontiers as upper bounds and benefits of lower targets of mortality reduction could be easily calculated. The consideration of opportunity costs to reach low mortality frontiers, which might be greater for LMICs, was beyond the scope of our analysis, even though we applied diminishing marginal utility of income as an attempt to incorporate increasing marginal opportunity costs of economic resources for delivering these large hypothetical benefits. Indeed, such adjustments were previously overlooked in the literature using constant VSL to estimate the benefits of large mortality reductions.

Lastly, we stress that our estimates of the economic value of reducing avoidable mortality are surrounded by large uncertainty, which is difficult to quantitively ascertain. Therefore, our work would be best interpreted and used in the context of intersectoral and high-level policy discussions.

In summary, we produced a systematic economic valuation of reducing the burden of avoidable mortality associated with diseases and injuries, globally and in major world regions. We found very large economic values linked to reducing avoidable NCD and injury mortality, amounting to several percentages of annual incomes. The economic implications of controlling NCDs and injuries would be substantial, particularly for CVDs, cancers and injuries. This prompts the urgent rolling out and scaling up of largely underused cost-effective preventive, curative and public health interventions in all countries. More broadly, our framework could also be adapted to estimate the economic returns from investing in health. In doing so, we derive an economic metric directly comparable to annual incomes, which can contribute to high-level policy discussions and enable both health sector and intersectoral priority setting, and which can directly speak to ministers of finance in highlighting the enormous value of reducing avoidable mortality.

## Methods

### General approach

We proceeded in three steps. First, we projected age–cause–sex-specific mortality rates into the future (2020–2050) for 113 countries (Supplementary Table 1). We estimated age–cause-specific mortality frontiers for the period 2000–2019, with the mortality frontiers being the lowest estimated mortality rates for a given cause and age group, globally. We then projected these frontiers over 2020–2050. They can be thought of as ‘aspirational’ mortality rates for countries that experience mortality rates greater than the frontier.

Second, we compared country- and cause-specific mortality rates with frontier rates to yield ‘avoidable mortality’, the gap between a country’s mortality rate and the frontier mortality rate. We aggregated avoidable mortality across six analytical geographic regions (Supplementary Table 1).

Third, we assigned a monetary value to reducing the avoidable mortality. This gave an estimated percentage of annual income an individual would be willing to forgo to live one year under the lowest possible mortality rate for a given death cause, and a metric comparable to national income (for example, GNI).

These three steps are further detailed below.

### Data sources

Estimates of age–cause–sex-specific death rates for all countries for 2000–2019 were from the WHO Global Health Estimates (GHE)^10^. We condensed the comprehensive list of disease and injury causes of death of WHO to a smaller list of mutually exclusive, collectively exhaustive set of causes primarily focused on NCDs and injuries (Table 2 and Supplementary Table 2). These causes of death were selected as they are leading causes of mortality according to burden of disease assessments^10,11^. For cancers, we focused on tobacco-related (for example, lung, mouth, esophagus) and infection-related (for example, cervix, liver, stomach) cancers; breast cancer was selected as it was the leading cause of cancer mortality among females^10,11^.

All calculations were done by sex and 5 year age groups (except for the first two and final age groups; 0–1, 1–4, 5–9,…, 80–84 and 85+ years). Age- and sex-specific population and all-cause mortality estimates (used as described below) for all countries for the period 2000–2050 were sourced from the UN 2022 World Population Prospects (WPP)^1^.

### Analytical approach

#### Estimating the mortality frontiers

Cause-of-death assignment practices and quality vary geographically and over time, and mortality rates are affected by stochastic variability. Because of this variability, the lowest observed or estimated cause-specific mortality rates may be implausible. We took two steps to ensure stability and minimize stochastic variation in the resulting computed frontiers. First, we selected mortality rates from countries if they had populations of at least 5 million in 2019 (to eliminate stochastic variability, which became quite important for populations smaller than 5 million), available income data (GNI per capita, current international dollars) for 2019, high-quality vital registration data (to reduce variability from low-quality data; as defined in ref. ^42^) and GHE estimates for 2019 (Supplementary Table 1). Second, we selected the 10th percentile of the age–cause-specific mortality rates of eligible countries in a given year, rather than the lowest mortality rate, to exclude countries with unusually low assignment of deaths to a particular cause^22,43^. The sum of 10th percentile cause-specific mortality rates by age and sex was closer to the lowest all-cause mortality rates than the sum of the lowest cause-specific mortality rates, implying that the 10th percentile results in a more plausible frontier cause profile. The frontiers were age and cause specific for all causes except for breast cancer and cervix uteri cancer, for which age–sex–cause-specific frontiers were computed given the sex-specific epidemiology of these two conditions (Note that male-specific cancers, such as testicular or prostate cancers, were not included as they are less common and affect older ages, so their overall burden is much lower.).

For 2000–2019, all-cause frontiers were obtained from our companion analysis^22^ and cause-specific frontiers were selected from GHE estimates. To ensure that the frontiers of lower-level causes appropriately summed to the frontiers of higher (‘parent’)-level causes (Table 2), we used a nested, level-wise approach. The frontiers for level 1 causes (communicable, maternal, perinatal and nutritional conditions; NCDs; and injuries) were summed to calculate the parent level (level 0: all-cause) frontier. The resulting calculated level 0 all-cause frontier was then compared with the all-cause frontier to determine a scaling factor. This scaling factor was then applied to the component level 1 frontiers. This process was continued for level 2 causes (for example, CVD, malignant neoplasms) and level 3 causes (for example, stroke, stomach cancer).

Log-linear regressions were fitted to 2000–2019 frontier mortality rates, separately for each age–cause or age–sex–cause combination, to project the 2020–2050 frontier mortality rates, consistent with previous analyses^36^ (Supplementary Information 1, pp.13–48). Finally, cause-specific frontier mortality rates were scaled to sum to all-cause frontier mortality from our companion analysis^22^, in the same nested, level-wise approach described above.

#### Estimating country mortality projections

We projected country–age–sex–cause-specific mortality rates for the period 2020–2050 using the same methods as for the frontier projections. We then aggregated these country-specific mortality rate projections within each region studied. For that purpose, we weighted country rates by country–age–sex-specific population from the 2022 WPP^1^ to yield region–age–sex–cause-specific mortality rate projections.

#### Economic value associated with reducing cause-specific avoidable mortality

We compared a country’s age group’s probability of dying (*C* = country) to the estimated frontier mortality rate of dying $(\mathcal{F})$, that is, *q*_*C*_(a) to ${q}_{\mathcal{F}}$(*a*), over 1 year periods. The difference in those probabilities is the avoidable mortality ${\delta}_{c,\mathcal{F}}$(*a*) = *q*_*C*_(*a*) − ${q}_{\mathcal{F}}$(*a*). We then assigned a value, *v*, to ${\delta}_{c,{\mathcal{F}}}$(*a*) that depends on the magnitude of ${\delta}_{c,\mathcal{F}}$ and on the VSL in country *C* (VSL_*C*_) using standard economic methods recommended by best practices documented in a reference case^14^ and extended further in our companion study^22^. The value that one places on reducing a mortality risk (proxied by ${\delta}_{c,\mathcal{F}}$) is often quantified with the VSL. The VSL captures the amount of money that one individual is willing to forgo in exchange for reducing her own mortality risk by a small amount, such as a 1 per 10,000 reduction in the probability of dying in a given year^14^.

We directly applied the methods developed in ref. ^22^, where *v* increases with ${\delta}_{c,{\mathcal{F}}}$ and its marginal rate of increase decreases with ${\delta}_{c,\mathcal{F}}$. That is, as avoidable mortality becomes greater, each additional reduction in the mortality risk would have a smaller economic value^22,32,39^.

Following closely best practices^14^ and their adaptation^22^, the value of VSC_*C*_ was extrapolated from the VSL in the United States (VSC_US_) using an income elasticity, which quantifies how the relative change in income determines the relative change in VSL. To derive VSL_*C*_, we set 0.8 as income elasticity for countries with GNI per capita greater than that of the United States and 1.2 for countries with lower GNI per capita^14,44^. (For countries with income lower than that of the United States, this means that individuals would dedicate a greater share of their income to other expenses and thus spend less proportionally than US residents on mortality reduction. For countries with higher income than the United States, this means that individuals would dedicate a smaller share of their income to other expenses and thus spend more proportionally than US residents on mortality reduction^14,22^) (Alternative income elasticity values were also tested in sensitivity analyses, as described below). We also set a floor constraint for the initial ratio between VSL and GNI per capita of 20. We used VSL_US_ = 160 times US GNI per capita, and a discount rate of 3% per year, again per best practices^14^.

GNI per capita was expressed in 2017 international dollars (adjusted for purchasing power parity)^45^ for the period 2000–2021, and projected to 2050 using Organisation for Economic Co-operation and Development (OECD) projected country-specific growth rates over 2021–2050 for listed countries (OECD and G20 countries) and the world average growth rate during the same time period for all remaining countries.

Therefore, per year, country, cause and age group, we derived an economic value further aggregated by geographic region, with China, India and high-income countries shown separately. We provided estimates for 2000, 2019 and 2050, which coincide with the beginning of major increases in development assistance for health, the last year before the onset of COVID, and a milestone year for major international goals. The economic value estimates provided are comparable to annual incomes and capture the percentage of income an individual would be willing to forgo to live 1 year under the lowest possible mortality rate for a given cause of death (in a given region); they are presented as percentage of annual income.

All the economic methods applied in this section directly build on best practices^14^ and our companion study^22^ (see additional details in Supplementary Information 1, p. 49).

#### Uncertainty and sensitivity analyses

First and foremost, we must highlight that there are major structural and parameter uncertainties accompanying the application of our approach and thus our estimations. Therefore, uncertainty around the estimates of economic value of reducing avoidable mortality we provide remains, to a large extent, unquantifiable.

While we acknowledge that we do not fully know to what extent our economic value estimates are reliable, we proceeded to conducting numerous univariate sensitivity analyses. First, we implemented an alternative benchmark for the mortality frontiers: we estimated the mortality frontiers in using the lowest cause-specific mortality rates (instead of the 10th percentile of mortality rates), but still scaling to the all-cause frontiers^22^.

Second, we applied the full set of sensitivity analyses following best practices^14^. Importantly, when quantifying economic values, resulting estimates are highly sensitive to VSL values assigned. This would not necessarily affect the relative distribution of the cause-specific allocations and economic values that we report in terms of multiples of annual income. Yet, cognizant of this sensitivity to VSL valuation, we applied two sets of standard sensitivity analyses related to VSL estimates, per best-practice recommendations^14^. That is, (1) we varied income elasticities to either a low (1.0) or high (1.5) value, and (2) we set an alternative baseline VSL-to-income ratio of 100 relative to the average income among OECD countries (instead of the US VSL-to-income ratio of 160). Lastly, per best-practice recommendations^14^, we applied alternative discount rates of either 1% or 5% per year (in place of 3% per year in the base case).

All computations and simulations were conducted using R software (version 2022.02.3).

## Ethics and inclusion statement

All data used in this study are secondary publicly available data. Therefore, ethical approvals were not required for this study.

Estimates are provided for the world and six regions, with those of high-income countries, India and China provided separately. This study does not examine a single country in depth. Yet, the estimates are locally relevant to all countries at the regional level.

We fully endorse collaboration and inclusivity in global health research. Therefore, with this work, we hope to generate interest from local researchers and policymakers including those from low- and middle-income countries to co-develop and co-author subsequent contextualized analyses at the country level in the future.

### Reporting summary

Further information on research design is available in the Nature Portfolio Reporting Summary linked to this article.

## Online content

Any methods, additional references, Nature Portfolio reporting summaries, source data, extended data, supplementary information, acknowledgements, peer review information; details of author contributions and competing interests; and statements of data and code availability are available at 10.1038/s41591-024-03248-4.

## Supplementary information

Supplementary Information Detailed methods and results.

Reporting Summary

## Data Availability

All data used in this study are secondary publicly available data. The WHO Global Health Estimates can be obtained from https://www.who.int/data/global-health-estimates. UN World Population Prospects estimates can be obtained from https://population.un.org/wpp/. The World Bank World Development Indicators can be obtained from https://datatopics.worldbank.org/world-development-indicators/. All datasets used are available via GitHub at https://github.com/sbolongaita/HLI_Cause-Specific-EVAM.

## Code availability

All codes, including the replication code, are available via GitHub at https://github.com/sbolongaita/HLI_Cause-Specific-EVAM.
