# Supplementary material for: The economic value of reducing mortality due to noncommunicable diseases and injuries
Source: Nat Med. 2024 Sep 27;30(11):3335–44. doi: 10.1038/s41591-024-03248-4 (PMC11564085; doi:10.1038/s41591-024-03248-4)
Supplement: Supplementary file 1 — Detailed methods and results. [file 41591_2024_3248_MOESM1_ESM.pdf]

nature medicine

Supplementary information https://doi.org/10.1038/s41591-024-03248-4

The economic value of reducing mortality due to noncommunicable diseases and injuries

In the format provided by the authors and unedited

Supplementary Information 1 – Detailed Methods

In this document, we give supplementary information related to the methods used in the paper to estimate the economic value associated with reducing avoidable mortality by cause of death and country, over the time period 2000-2050.

1. Analytical regions and cause of death mapping

Table 1 describes the analytical regions and the different countries constituting these regions. Table 2 details the cause of death mapping conducted in the analysis.

Table 1. Analytical regions

Countries with populations of at least five million in 2019 and with available income (i.e., gross national income (GNI) per capita) data for 2019 were included in the analysis. Of the countries included, those with high-quality vital registration data and which were included in the World Health Organization's Global Health Estimates (GHE) for 2019 were included in the frontier analysis (denoted by * in the table).

China			
China			
Eurasia and the Mediterranean			
Afghanistan	Algeria	Azerbaijan	Bangladesh
Belarus	Bulgaria	Cambodia	Egypt
Indonesia	Iran	Iraq	Jordan
Kazakhstan	Kyrgyzstan	Lao PDR	Lebanon
Libya	Malaysia	Morocco	Myanmar
Nepal	Pakistan	Papua New Guinea	Philippines *
Romania *	Russia	Serbia *	Sri Lanka
Tajikistan	Thailand	Tunisia	Turkey
Turkmenistan	Ukraine	Uzbekistan	Vietnam
High-income			
Australia *	Austria *	Belgium *	Canada *
Chile *	Czechia *	Denmark *	Finland *
France *	Germany *	Greece	Hungary *
Israel *	Italy *	Japan *	Netherlands *
Norway *	Poland	Portugal *	Saudi Arabia
Singapore *	Slovakia *	South Korea *	Spain *
Sweden *	Switzerland *	United Arab Emirates	United Kingdom *
United States *			
India			
India			
Latin America and the Caribbean			
Argentina	Bolivia	Brazil *	Colombia *
Costa Rica *	Dominican Republic	Ecuador	El Salvador
Guatemala	Haiti	Honduras	Mexico *

Nicaragua *	Paraguay	Peru	
**Sub-Saharan Africa**			
Angola	Benin	Burkina Faso	Burundi
Cameroon	Central African Republic	Chad	Congo
Côte d'Ivoire	Democratic Republic of Congo	Ethiopia	Ghana
Guinea	Kenya	Madagascar	Malawi
Mali	Mozambique	Niger	Nigeria
Rwanda	Senegal	Sierra Leone	Somalia
South Africa	Sudan	Tanzania	Togo
Uganda	Zambia	Zimbabwe	

* Included in the frontier analysis

Table 2. Cause of death mapping

The causes of death used in the analysis were adapted from a comprehensive set of causes put forth by the World Health Organization (WHO) in its Global Health Estimates (GHE) (WHO, 2020) [10]. Level 1 and 2 causes of death are mutually exclusive and collectively exhaustive; within level 2 causes of death, level 3 causes are mutually exclusive and collectively exhaustive. The table below shows the mapping between the causes of death used in this analysis and the original causes of death used in the WHO GHE data.

Level	Analysis cause of death	Original WHO GHE cause of death	
	Cause	Code	Cause
1	Communicable, maternal, perinatal and nutritional conditions	10	Communicable, maternal, perinatal and nutritional conditions
2	Infectious and parasitic diseases	20	Infectious and parasitic diseases
		380	Respiratory infections
2	Maternal and neonatal conditions	420	Maternal conditions
		490	Neonatal conditions
2	Nutritional deficiencies	540	Nutritional deficiencies
1	Noncommunicable diseases	600	Noncommunicable diseases
2	Cardiovascular diseases	1100	Cardiovascular diseases
3	Ischaemic heart disease	1130	Ischaemic heart disease
3	Stroke	1140	Stroke
3	Other cardiovascular diseases	1110	Rheumatic heart disease
		1120	Hypertensive heart disease
		1150	Cardiomyopathy, myocarditis, endocarditis
		1160	Other circulatory diseases
2	Diabetes mellitus	800	Diabetes mellitus
2	Digestive diseases	1210	Digestive diseases
3	Cirrhosis of the liver	1230	Cirrhosis of the liver
3	Other digestive diseases	1220	Peptic ulcer disease
		1240	Appendicitis
		1241	Gastritis and duodenitis
		1242	Paralytic ileus and intestinal obstruction

Level	Analysis cause of death	Original WHO GHE cause of death	
		1244	Inflammatory bowel disease
		1246	Gallbladder and biliary diseases
		1248	Pancreatitis
		1250	Other digestive diseases
2	Malignant neoplasms	610	Malignant neoplasms
3	Breast cancer	700	Breast cancer
3	Cervix uteri cancer	710	Cervix uteri cancer
3	Liver cancer	660	Liver cancer
3	Mouth and oropharynx cancers	620	Mouth and oropharynx cancers
3	Oesophagus cancer	630	Oesophagus cancer
3	Stomach cancer	640	Stomach cancer
3	Trachea, bronchus, lung cancers	680	Trachea, bronchus, lung cancers
3	Other malignant neoplasms	650	Colon and rectum cancers
		670	Pancreas cancer
		690	Melanoma and other skin cancers
		720	Corpus uteri cancer
		730	Ovary cancer
		740	Prostate cancer
		742	Testicular cancer
		745	Kidney cancer
		750	Bladder cancer
		751	Brain and nervous system cancers
		752	Gallbladder and biliary tract cancer
		753	Larynx cancer
		754	Thyroid cancer
		755	Mesothelioma
		760	Lymphomas, multiple myeloma
		770	Leukaemia
		780	Other malignant neoplasms
2	Respiratory diseases	1170	Respiratory diseases
3	Chronic obstructive pulmonary disease	1180	Chronic obstructive pulmonary disease
3	Other respiratory diseases	1190	Asthma
		1200	Other respiratory diseases
2	Other noncommunicable diseases	790	Other neoplasms
		810	Endocrine, blood, immune disorders
		820	Mental and substance use disorders
		940	Neurological conditions
		1020	Sense organ diseases
		1260	Genitourinary diseases
		1330	Skin diseases
		1340	Musculoskeletal diseases
		1400	Congenital anomalies
		1470	Oral conditions

Level	Analysis cause of death	Original WHO GHE cause of death	
		1505	Sudden infant death syndrome
1	Injuries	1510	Injuries
2	Intentional injuries	1600	Intentional injuries
2	Unintentional injuries	1520	Unintentional injuries
3	Road injury	1530	Road injury
3	Other unintentional injuries	1540	Poisonings
		1550	Falls
		1560	Fire, heat and hot substances
		1570	Drowning
		1575	Exposure to mechanical forces
		1580	Natural disasters
		1590	Other unintentional injuries

2. Estimating the mortality frontiers

Figure 1 compares two definitions of mortality frontiers, the 10th percentile of eligible country mortality rates and the minimum of eligible country mortality rates, for different years and different causes of death. It clearly shows that for the 10th percentile of eligible country mortality rates, that is the frontier retained in our analysis, ensures stability and minimizes stochastic variation in the resulting computed frontiers.

Figure 1. Comparison of frontier definitions

Two definitions of the frontier were assessed: the minimum and the 10th percentile of frontier eligible-country age- and sex-specific mortality rates. The figures below depict the frontier using both definitions prior to any further processing (i.e., harmonization, projection, scaling). The 10th percentile definition was selected for the analysis, as the resulting frontier was more stable and had less stochastic variation compared to the frontier resulting from the minimum definition.

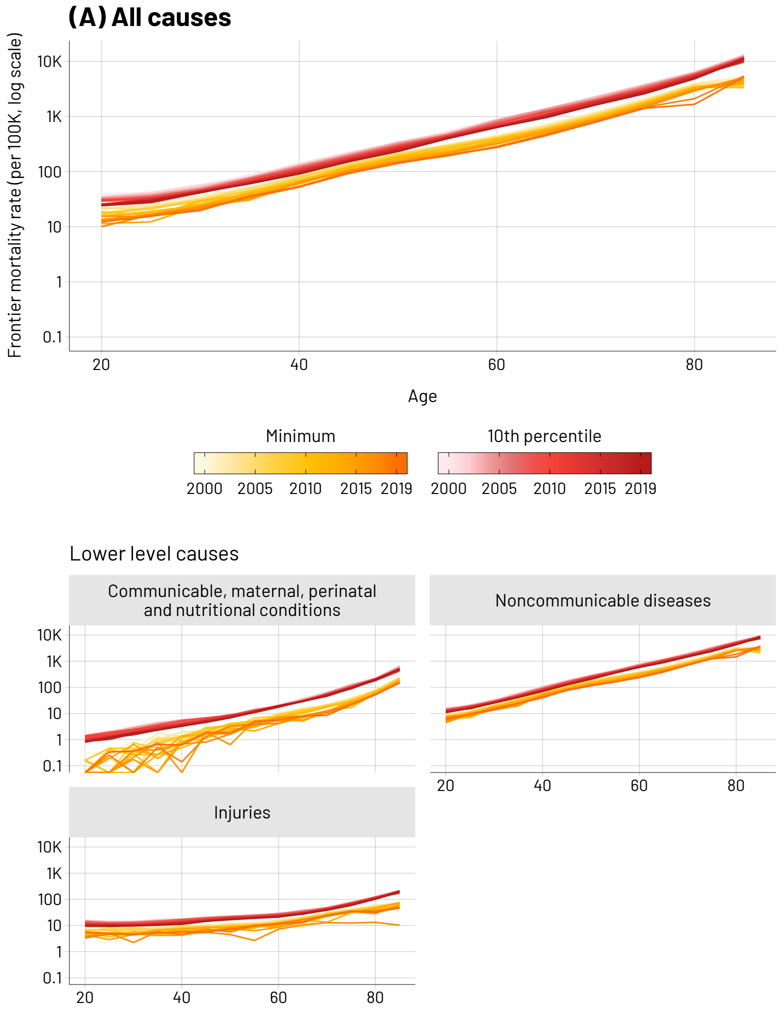

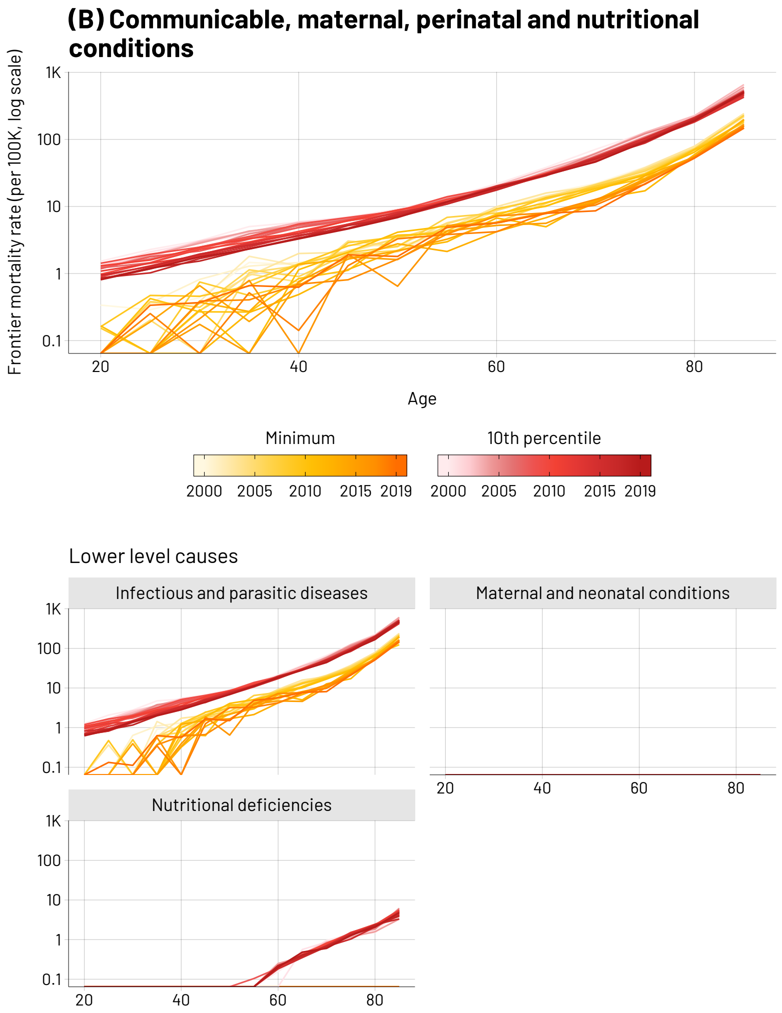
(B) Communicable, maternal, perinatal and nutritional conditions
Frontier mortality rate (per 100K, log scale)
1K
100
10
1
0.1
20
40
60
80
Age
Minimum
10th percentile
2000 2005 2010 2015 2019
2000 2005 2010 2015 2019
Lower level causes
Infectious and parasitic diseases
Maternal and neonatal conditions
Nutritional deficiencies

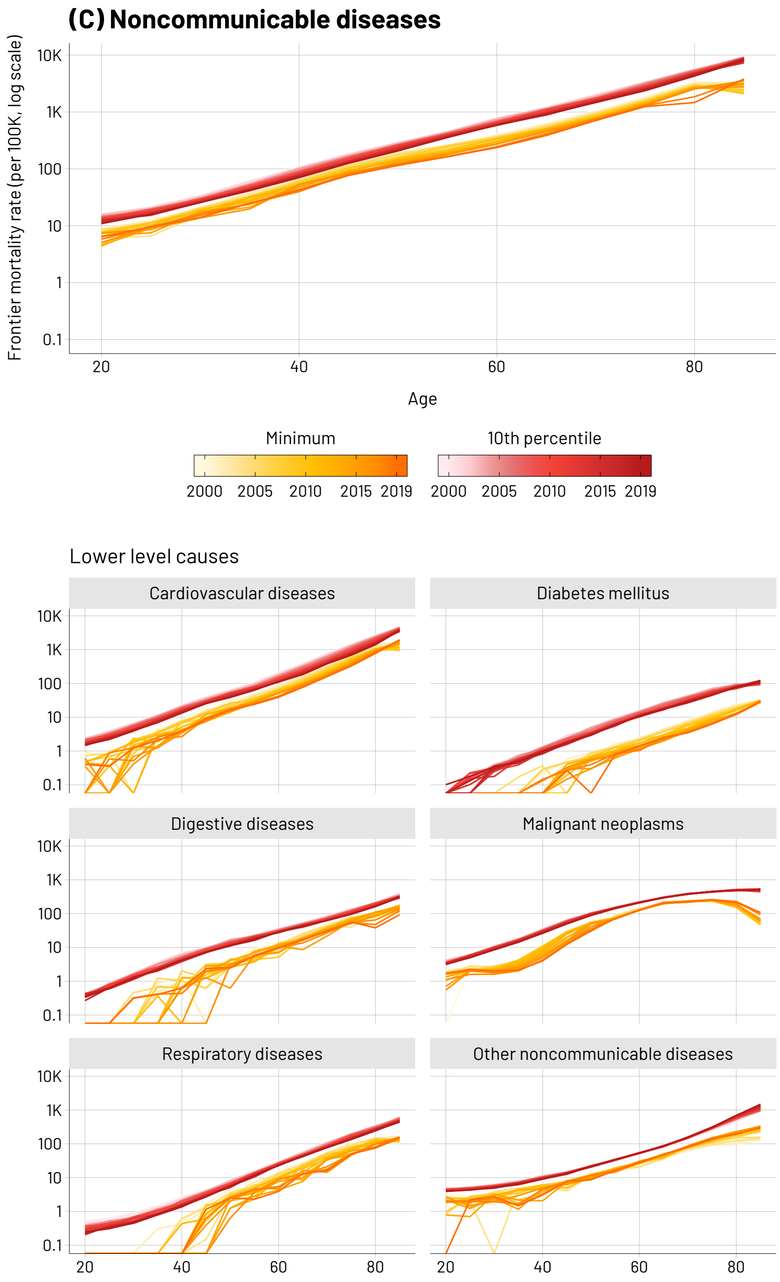

(C) Noncommunicable diseases
Frontier mortality rate (per 100K, log scale)
10K
1K
100
10
1
0.1
20
40
60
80
Age
Minimum
10th percentile
2000 2005 2010 2015 2019
2000 2005 2010 2015 2019
Lower level causes
Cardiovascular diseases
Diabetes mellitus
Digestive diseases
Malignant neoplasms
Respiratory diseases
Other noncommunicable diseases

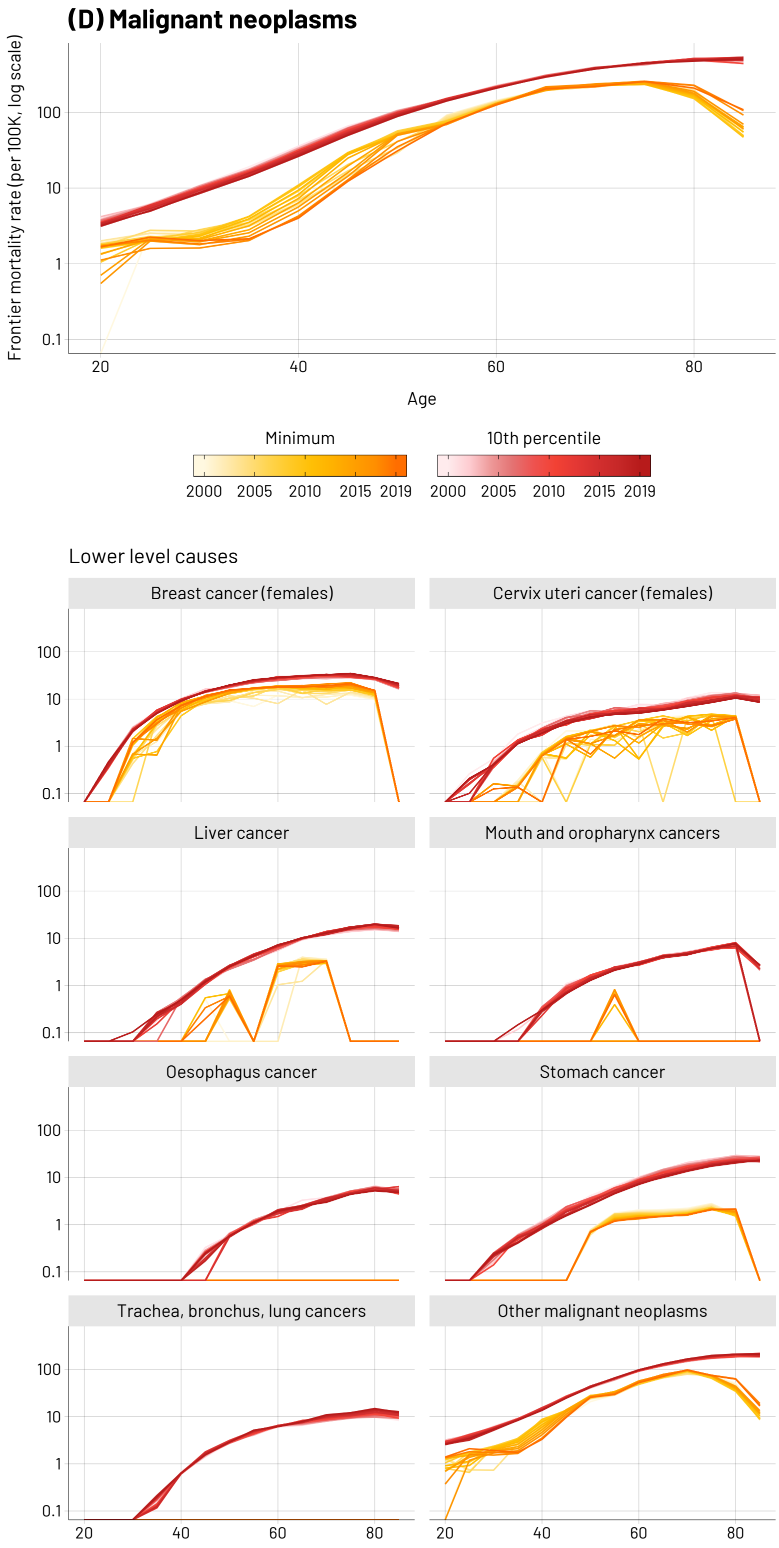
(D) Malignant neoplasms
Frontier mortality rate (per 100K, log scale)
100
10
1
0.1
20
40
60
80
Age
Minimum
10th percentile
2000 2005 2010 2015 2019
2000 2005 2010 2015 2019
Lower level causes
Breast cancer (females)
Cervix uteri cancer (females)
Liver cancer
Mouth and oropharynx cancers
Oesophagus cancer
Stomach cancer
Trachea, bronchus, lung cancers
Other malignant neoplasms

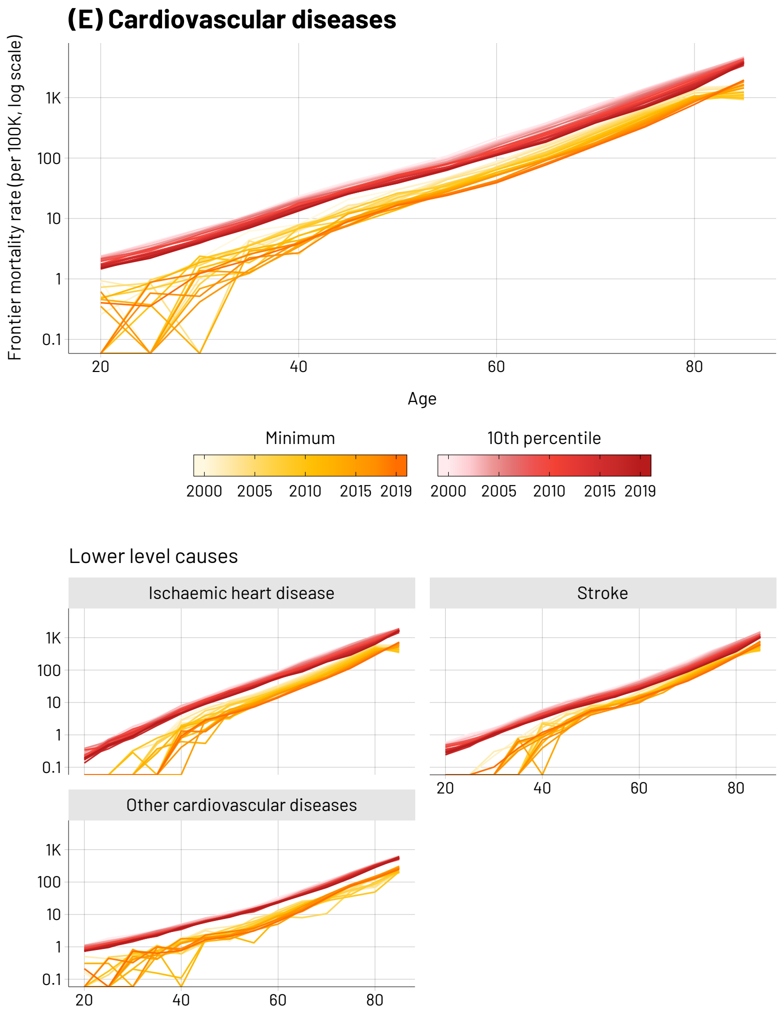

(E) Cardiovascular diseases
Frontier mortality rate (per 100K, log scale)
1K
100
10
1
0.1
20
40
60
80
Age
Minimum
2000 2005 2010 2015 2019
10th percentile
2000 2005 2010 2015 2019
Lower level causes
Ischaemic heart disease
Stroke
Other cardiovascular diseases

(F) Respiratory diseases

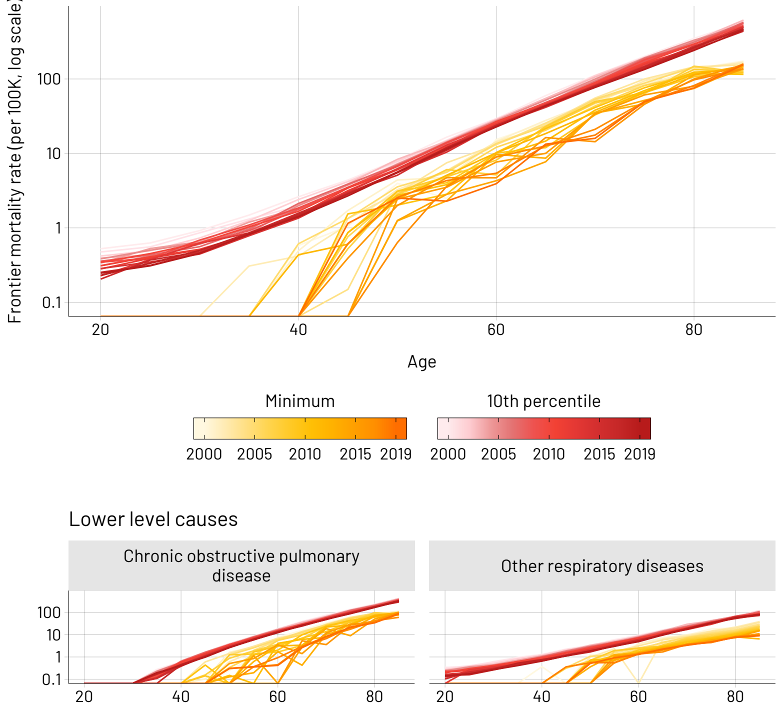

(G) Digestive diseases

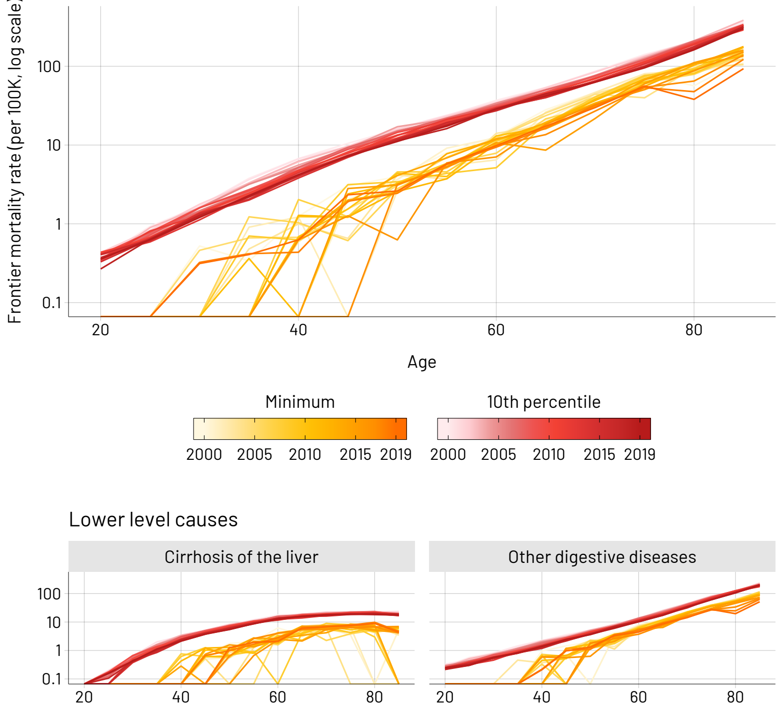

(H) Injuries

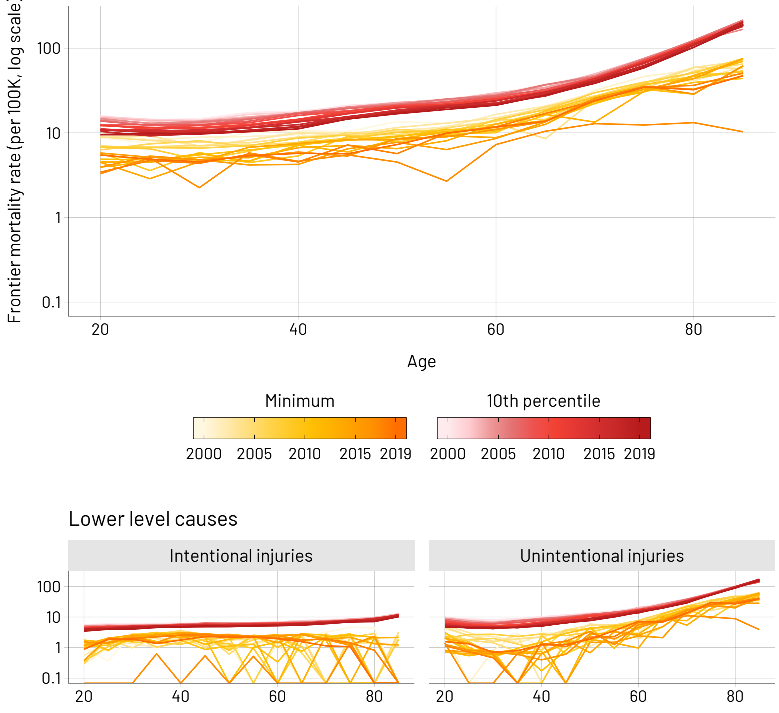

(I) Unintentional injuries

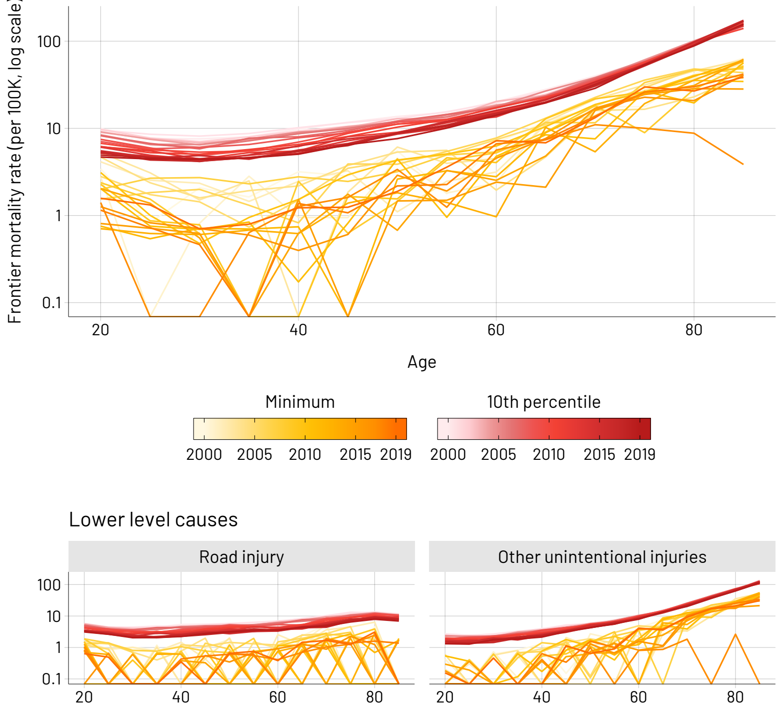

Frontier projection method

For the period 2020-2050, the frontiers were calculated based on the trends underlying the 2010-2019 frontiers. Log-linear models were used to project the frontier mortality rates using the following equation:

$$ln(M_{a,K,Y}) \sim C_{a,K} + \beta_{A,K} * Y \,, \qquad [1]$$

where $M_{a,K,Y}$ was the mortality rate for age group a in year Y (2000, 2019, and 2050), and cause of death K. $C_{a,K}$ was a constant term and Y was the year. $\beta_{A,K}$ was the estimated annual rate of change (decline) of the mortality rate associated with each cause and broad age group A (0-4, 5-14, 15-29, 30-44, 45-59, 60-69, 70-84, and 85+ year-olds). The projections (based on $\beta_{A,K}$ and [1]) were conducted separately by cause K and broad age group A to ensure similar mortality trends across neighboring five-year age groups, consistent with previous projection analyses (Mathers & Loncar 2006[37]).

When the 2010-2019 frontier mortality rates contained a zero-value (i.e., an observation of 0 deaths per 100,000 population) or when the average number of deaths (averaged over the 2010-2019 frontiers) for a given age-cause group was less than 100, then the average mortality rate (for the period 2010-2019) was used for the 2020-2050 projection for all five-year age groups within the corresponding broad age group category (yielding a constant frontier over 2020-2050). Additionally, if the regression ([1]) gave a positive trendline ($\beta_{A,K} > 0$, which would indicate increasing frontier rates over time), then the average mortality rate (across years) was also used for all five-year age groups within the corresponding broad age group category (Table A3).

To ensure consistency with all-cause mortality frontiers (i.e., Chang and colleagues' all-cause mortality frontiers of avoidable mortality[23]), our all-cause frontier mortality rates were scaled to these all-cause frontiers. We divided our age- and year-specific all-cause frontiers by the corresponding external age- and year-specific all-cause frontier mortality rates to produce a set of age-year-specific scaling factors between our all-cause frontiers and the Chang and colleagues' frontiers. The resulting scalars were then applied to our cause-specific frontiers (i.e., cause levels 1, 2, and 3) to ensure consistency with the avoidable mortality frontiers put forward by Chang and colleagues (Figure A2).

Table 3. Frontier projection method

Ordinary least squares (OLS) regression was used to project frontier mortality rates into the future (the period 2020-2050) unless a given broad age-cause group: (a) had an average annual number of deaths less than 100 (denoted by * in the table), (b) contained zero-value frontier mortality rate (i.e., an observation of 0 deaths per 100,000 population; denoted by †), or (c) had an OLS regression that yielded a positive trendline (denoted by ‡). In those cases, the average frontier mortality rate over 2010-2019 was used for the period 2020-2050.

Level	Cause	30-44 years	45-59 years	60-69 years	70-84 years	85+ years
0	All causes	OLS	OLS	OLS	OLS	Average ‡
1	Communicable, maternal, perinatal and nutritional conditions	Average *	OLS	OLS	OLS	Average ‡
2	Infectious and parasitic diseases	Average *	OLS	OLS	OLS	Average ‡
2	Maternal and neonatal conditions	Average *†	Average *†	Average *†	Average *†	Average *†
2	Nutritional deficiencies	Average *†	Average *†	Average *	Average *	Average *
1	Noncommunicable diseases	OLS	OLS	OLS	OLS	Average ‡
2	Cardiovascular diseases	Average *	OLS	OLS	OLS	OLS
3	Ischaemic heart disease	Average *	OLS	OLS	OLS	OLS
3	Stroke	Average *	Average *	OLS	OLS	OLS
3	Other cardiovascular diseases	Average *	Average *	OLS	OLS	Average ‡
2	Diabetes mellitus	Average *	Average *	OLS	OLS	Average ‡
2	Digestive diseases	Average *	OLS	OLS	OLS	Average ‡
3	Cirrhosis of the liver	Average *	Average *	OLS	Average *	Average *
3	Other digestive diseases	Average *	Average *	OLS	OLS	Average ‡
2	Malignant neoplasms	OLS	OLS	OLS	OLS	Average ‡
3	Breast cancer (females)	OLS	OLS	OLS	Average *	Average *
3	Breast cancer (males)	Average *†	Average *†	Average *†	Average *†	Average *†
3	Cervix uteri cancer (females)	Average *	Average *	Average *	Average *	Average *
3	Cervix uteri cancer (males)	Average *†	Average *†	Average *†	Average *†	Average *†
3	Liver cancer	Average *†	Average *	Average *	Average *	Average *
3	Mouth and oropharynx cancers	Average *†	Average *	Average *	Average *	Average *†
3	Oesophagus cancer	Average *†	Average *†	Average *	Average *	Average *
3	Stomach cancer	Average *	Average *	Average *	Average *	Average *
3	Trachea, bronchus, lung cancers	Average *†	Average *	Average *	Average *	Average *

Level	Cause	30-44 years	45-59 years	60-69 years	70-84 years	85+ years
3	Other malignant neoplasms	OLS	OLS	OLS	OLS	Average *
2	Respiratory diseases	Average *	Average *	OLS	OLS	OLS
3	Chronic obstructive pulmonary disease	Average *	Average *	OLS	OLS	OLS
3	Other respiratory diseases	Average *	Average *	OLS	OLS	OLS
2	Other noncommunicable diseases	OLS	OLS	OLS	Average ‡	Average ‡
1	Injuries	OLS	OLS	OLS	OLS	Average ‡
2	Intentional injuries	Average *	Average *	Average *	Average *	Average *
2	Unintentional injuries	Average *	Average *	OLS	OLS	Average ‡
3	Road injury	Average *	Average *	Average *	Average *	Average *
3	Other unintentional injuries	Average *	Average *	Average *	Average *	Average *

* Average number of deaths less than 100
† Frontier mortality rates contain 0
‡ OLS linear regression indicated positive trend

Figure 2. Frontier projection process

After the cause-specific frontiers were extracted, using the 10th percentile definition ("Base", see Figure 1), mortality rates were harmonized such that the mortality rates of lower-level causes of death summed to the mortality rates of higher, parent-level causes ("Harmonized"). Log cause-specific frontiers were then projected using ordinary least squares (OLS) regression or an average mortality rate ("Projected", see Table 3). Scaling factors were used to ensure that the cause-specific frontiers put forth in this analysis were consistent with the all-cause frontiers estimated by Chang and colleagues[23] in their assessment of avoidable mortality ("Scaled").

(A) All causes

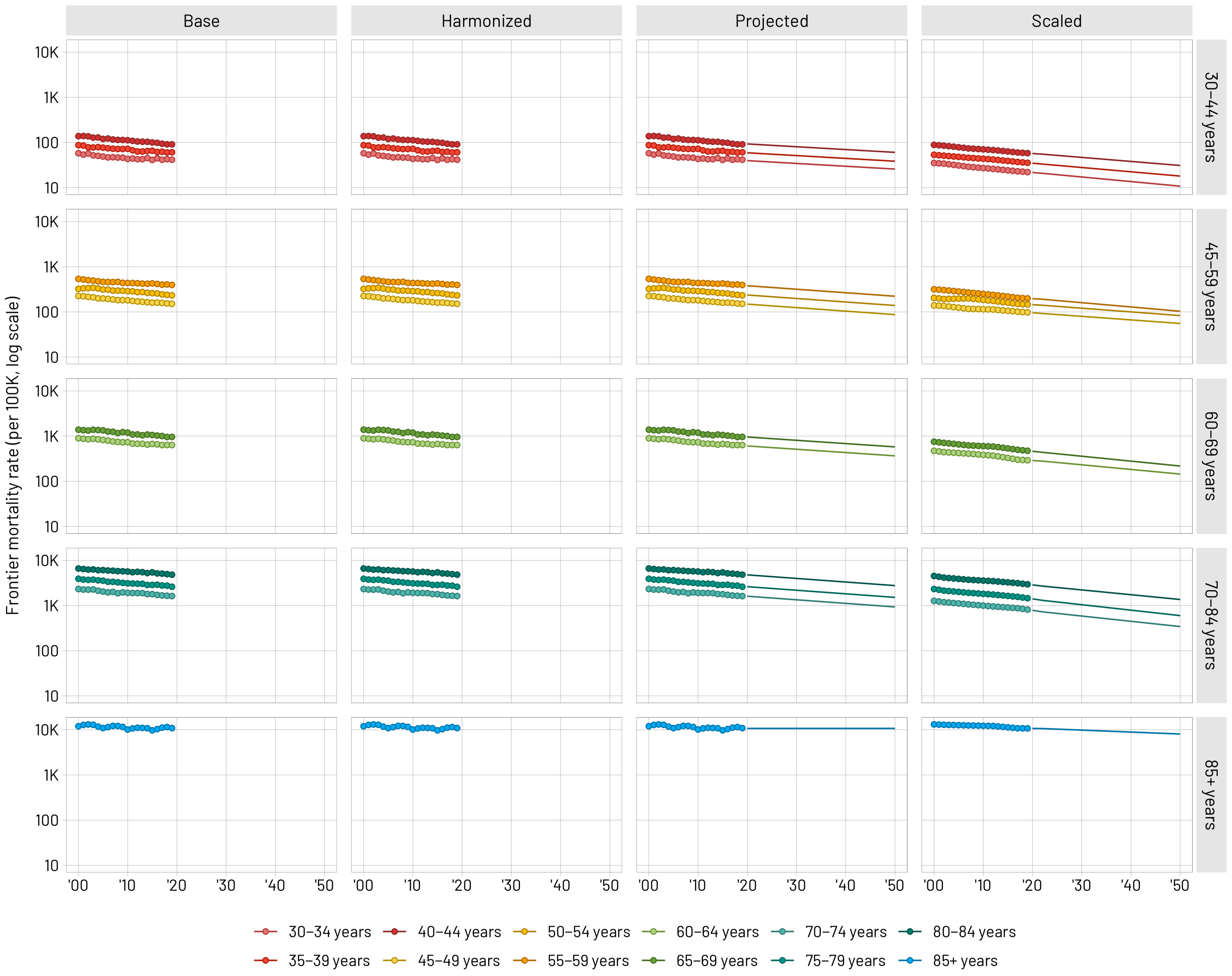

Base
Harmonized
Projected
Scaled
30–44 years
45–59 years
60–69 years
70–84 years
85+ years
Frontier mortality rate (per 100K, log scale)
10K
1K
100
10
'00
'10
'20
'30
'40
'50
30–34 years
35–39 years
40–44 years
45–49 years
50–54 years
55–59 years
60–64 years
65–69 years
70–74 years
75–79 years
80–84 years
85+ years

(B) Communicable, maternal, perinatal and nutritional conditions

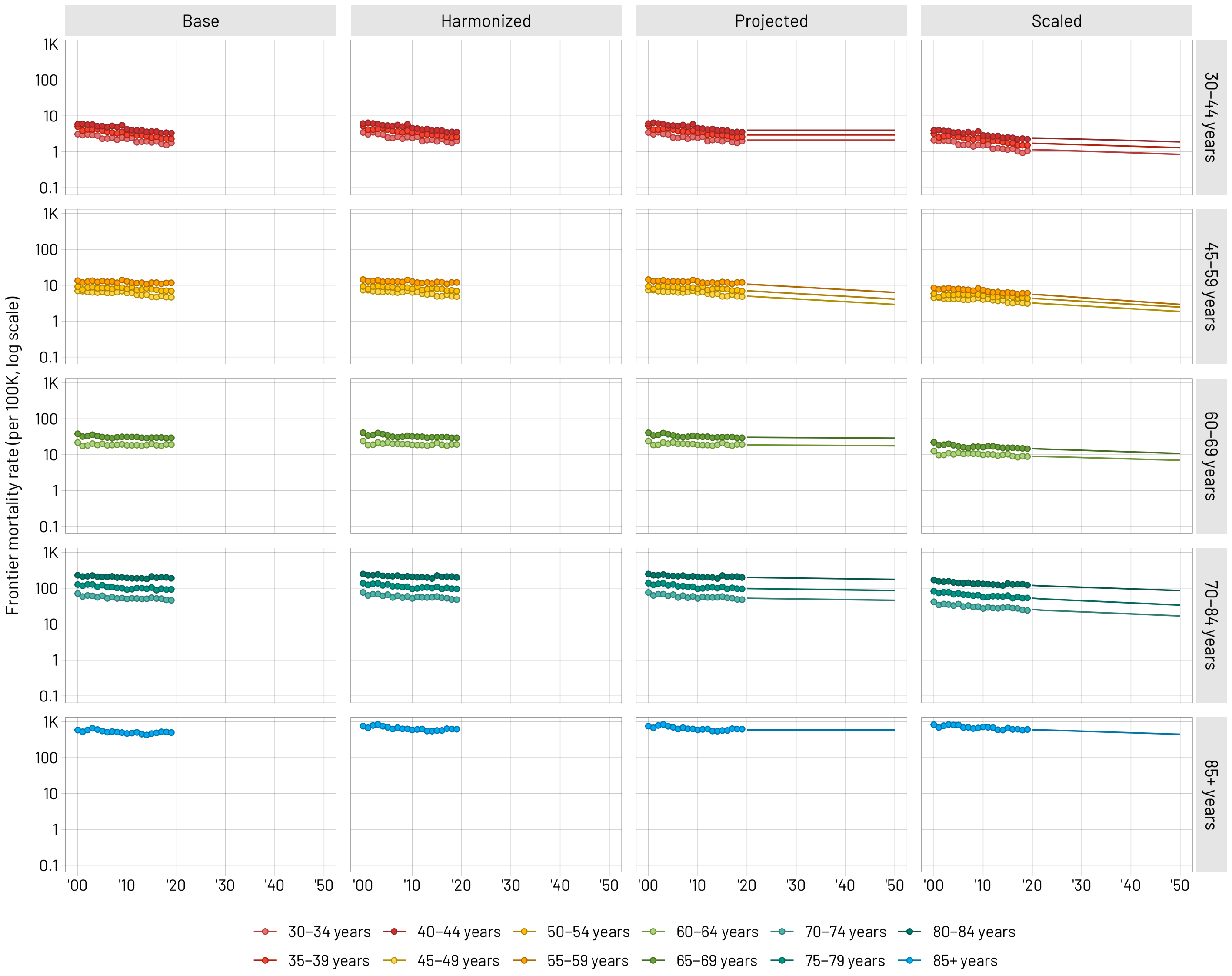

(C) Infectious and parasitic diseases

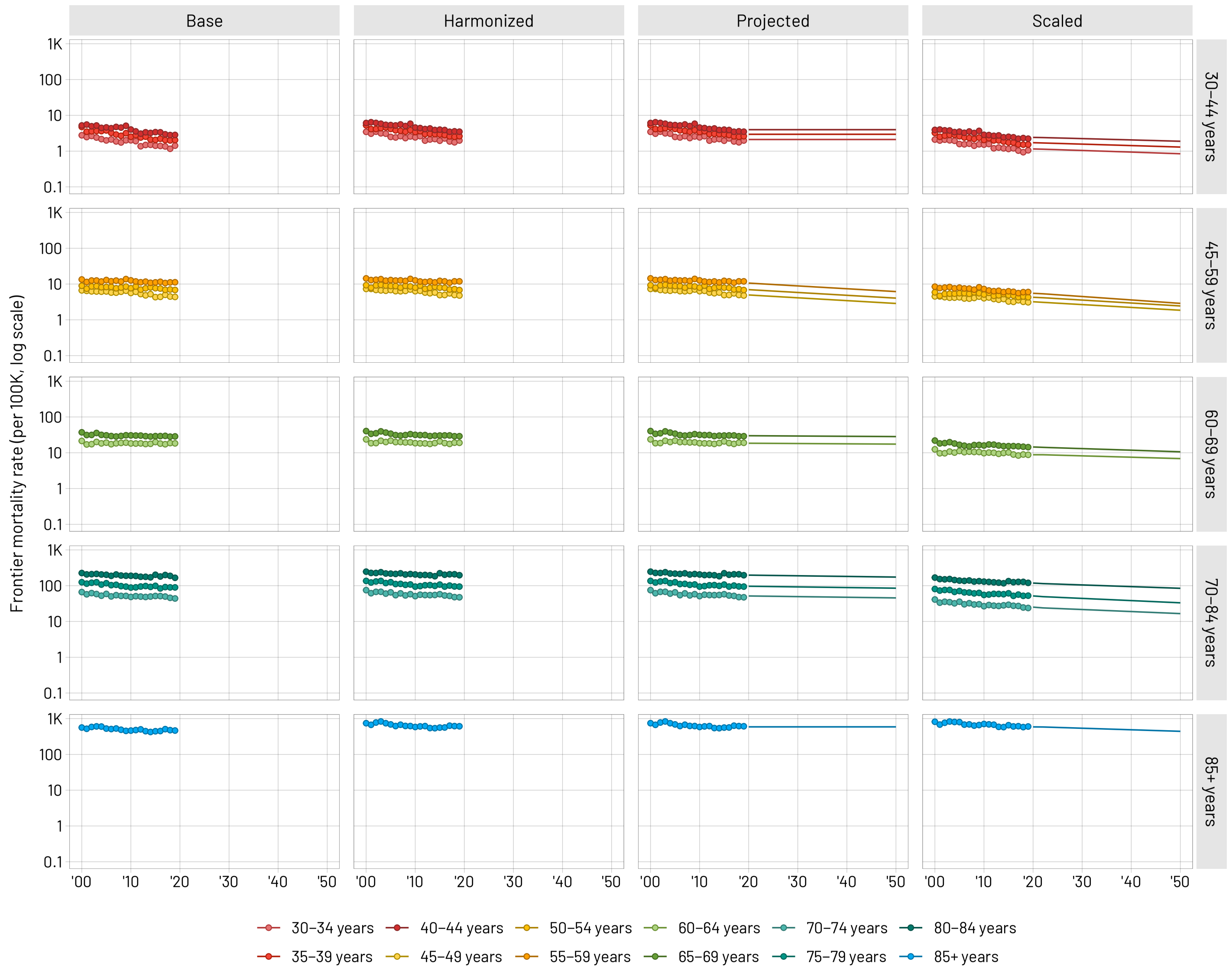

(D) Maternal and neonatal conditions

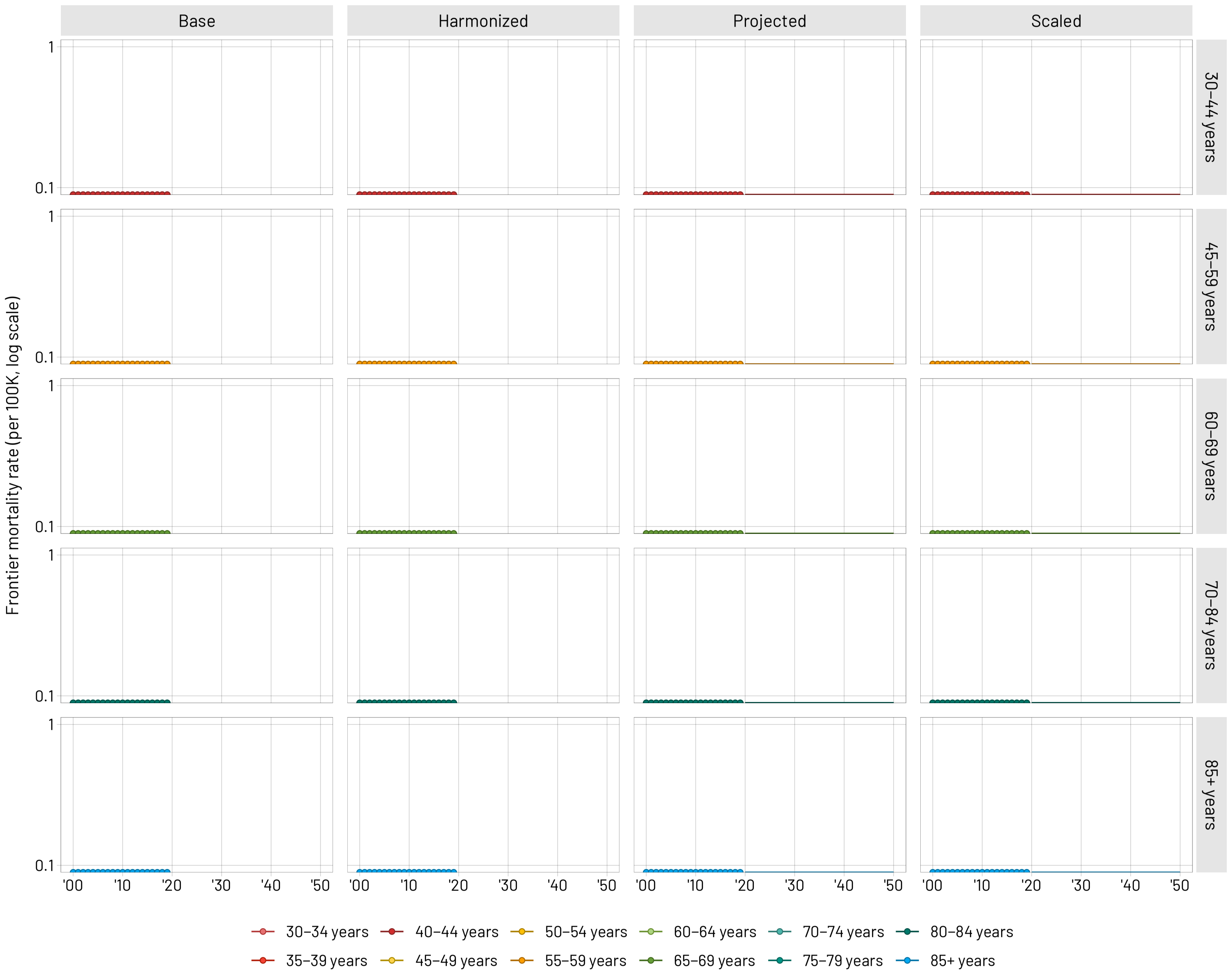

(E) Nutritional deficiencies

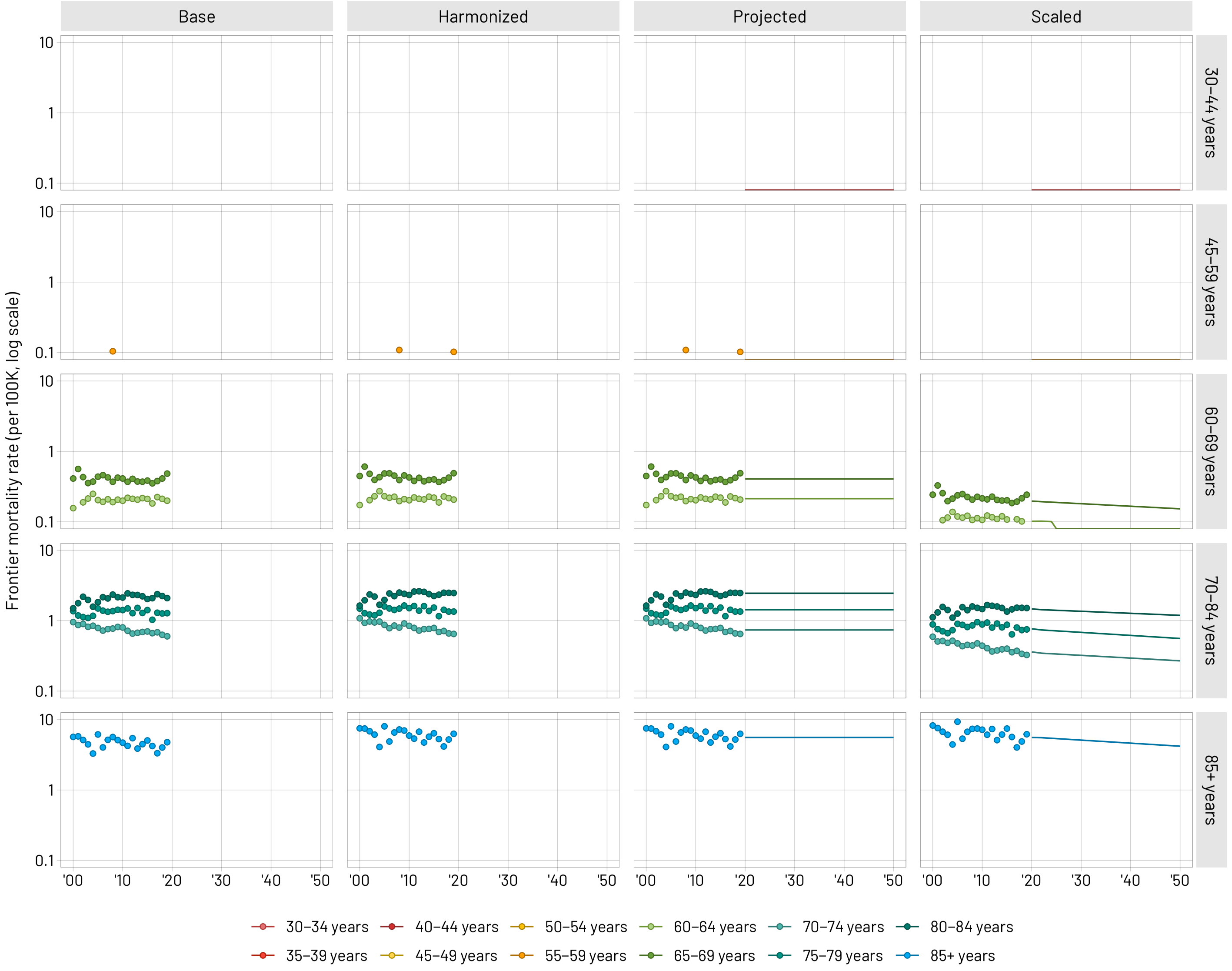

(F) Noncommunicable diseases

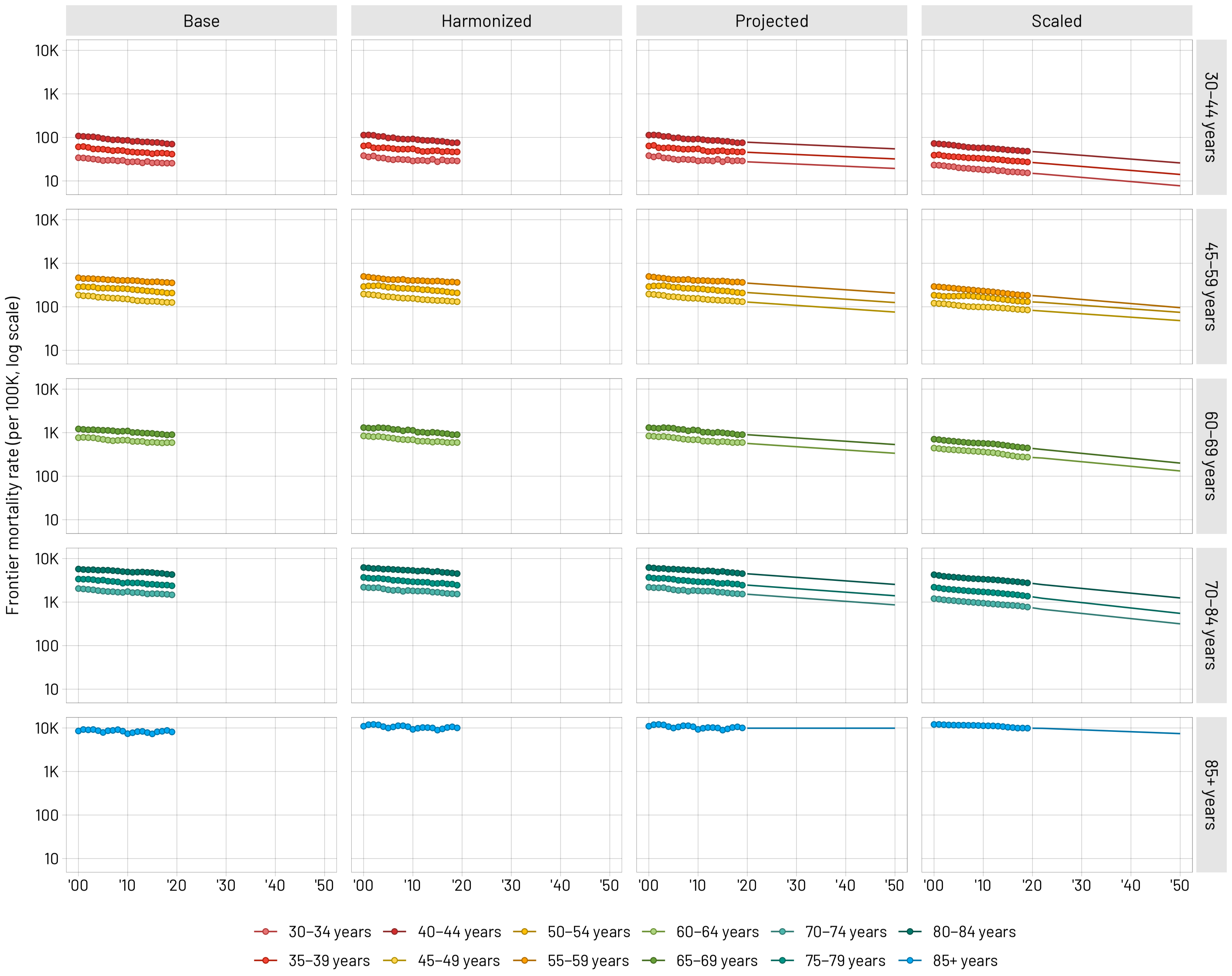

(G) Malignant neoplasms

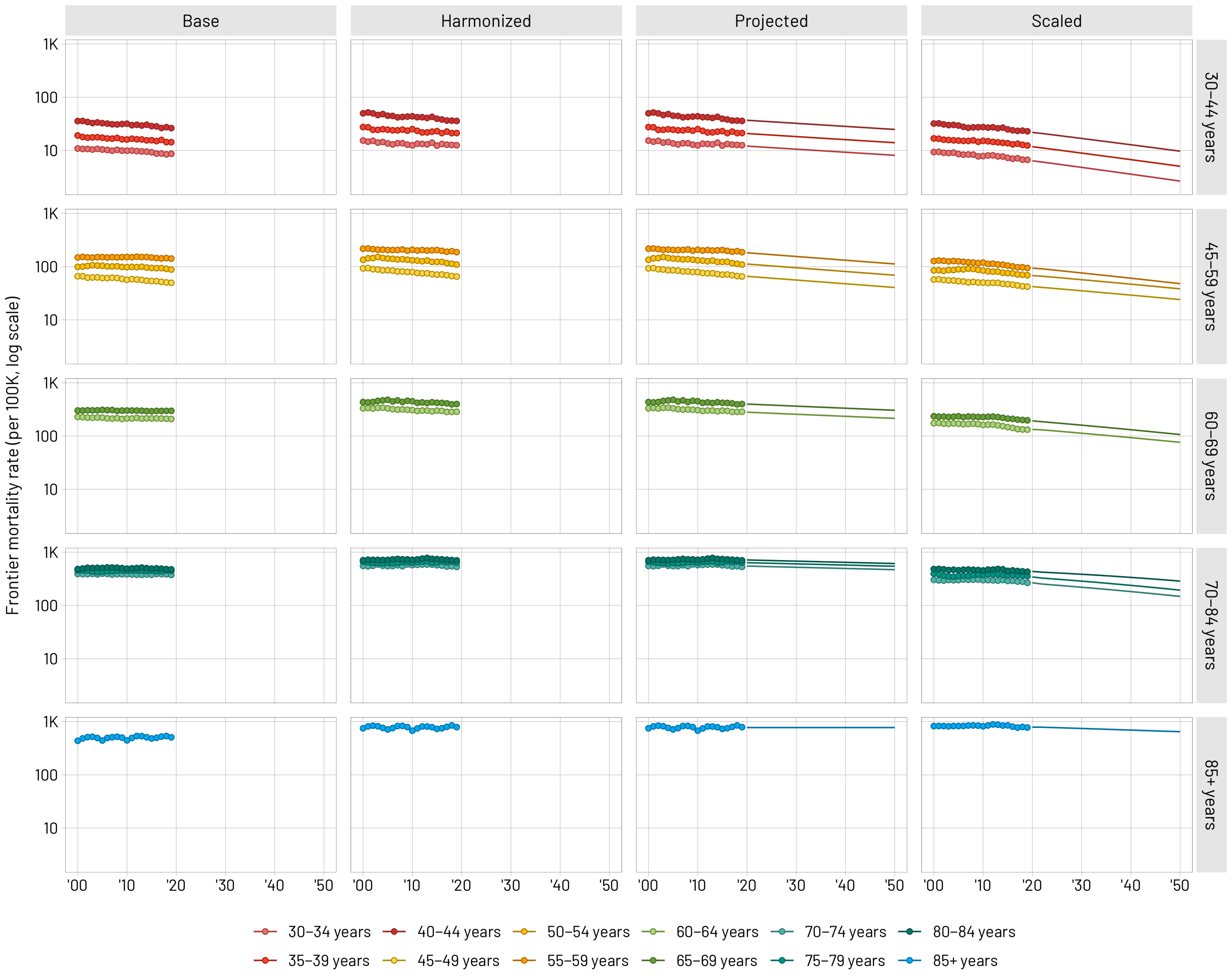

(H) Mouth and oropharynx cancers

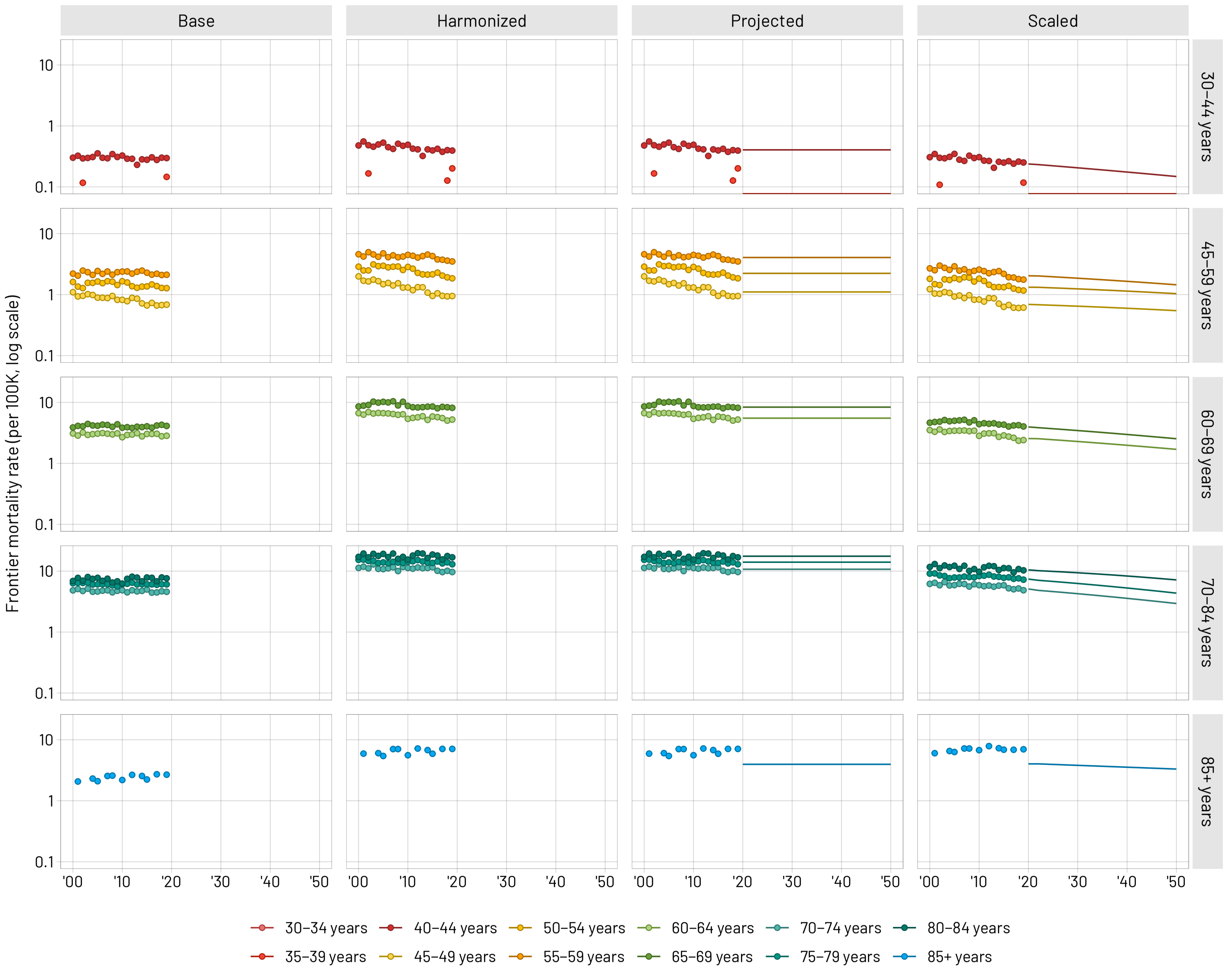

(l) Oesophagus cancer

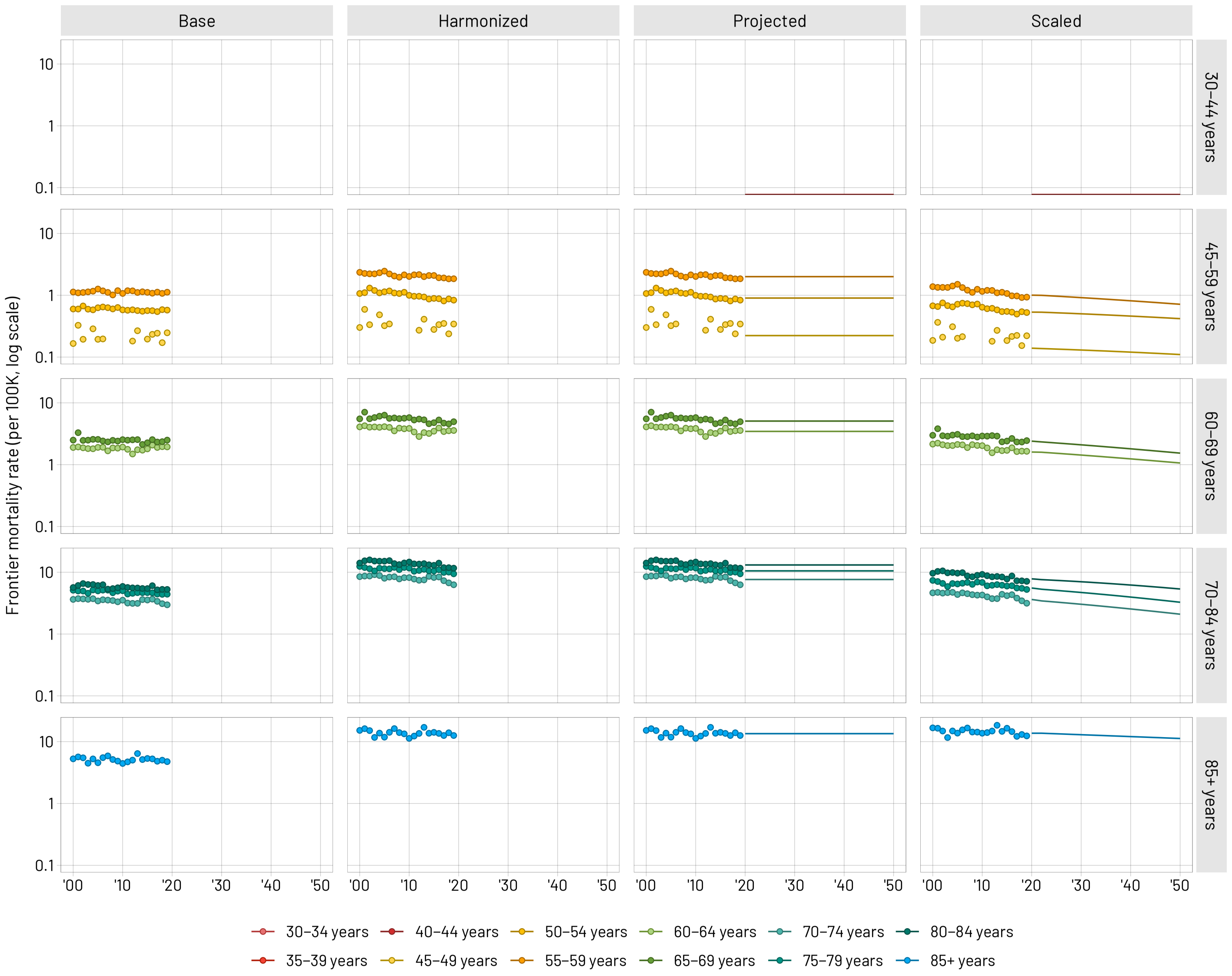

(J) Stomach cancer

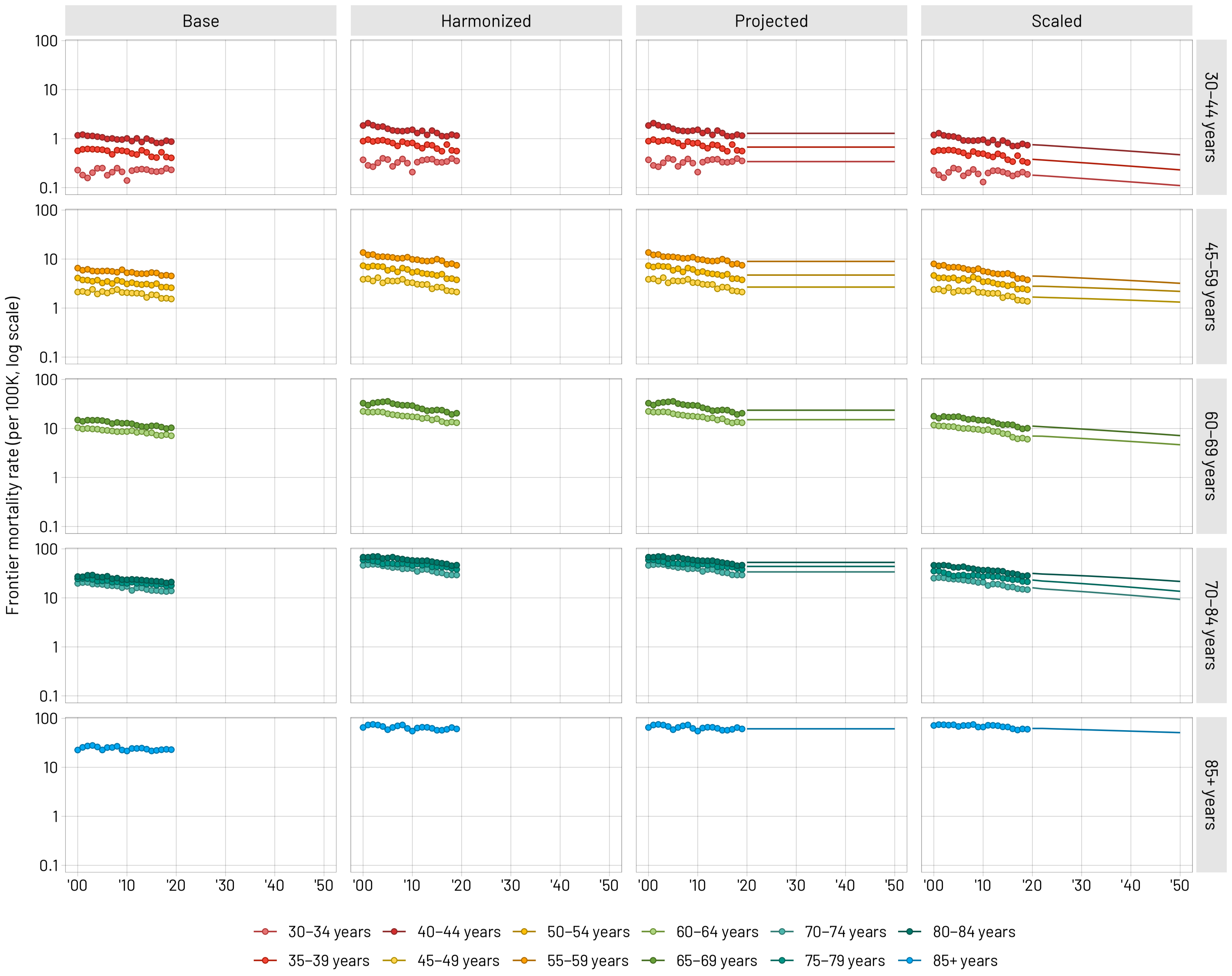

(K) Liver cancer

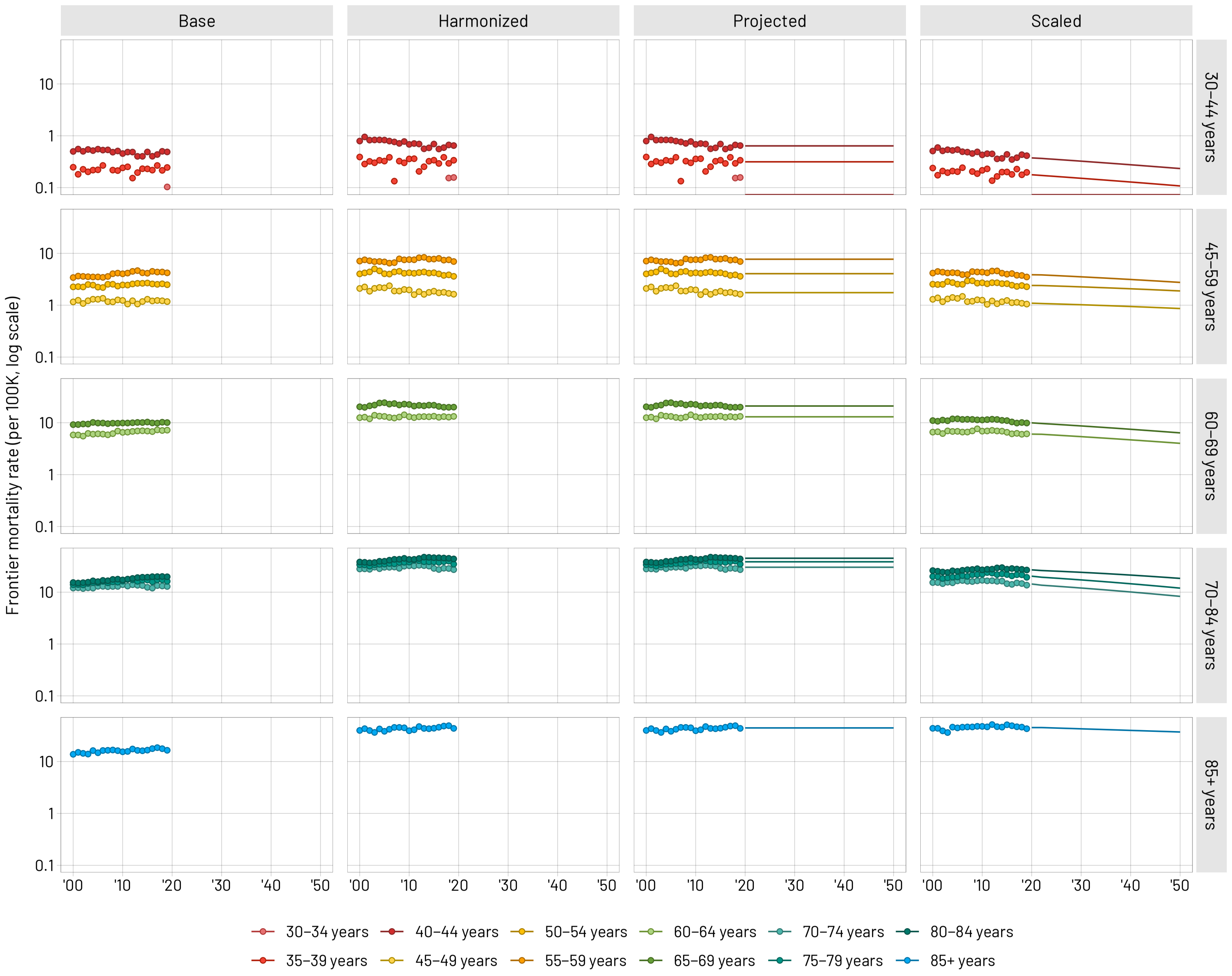

(L) Trachea, bronchus, lung cancers

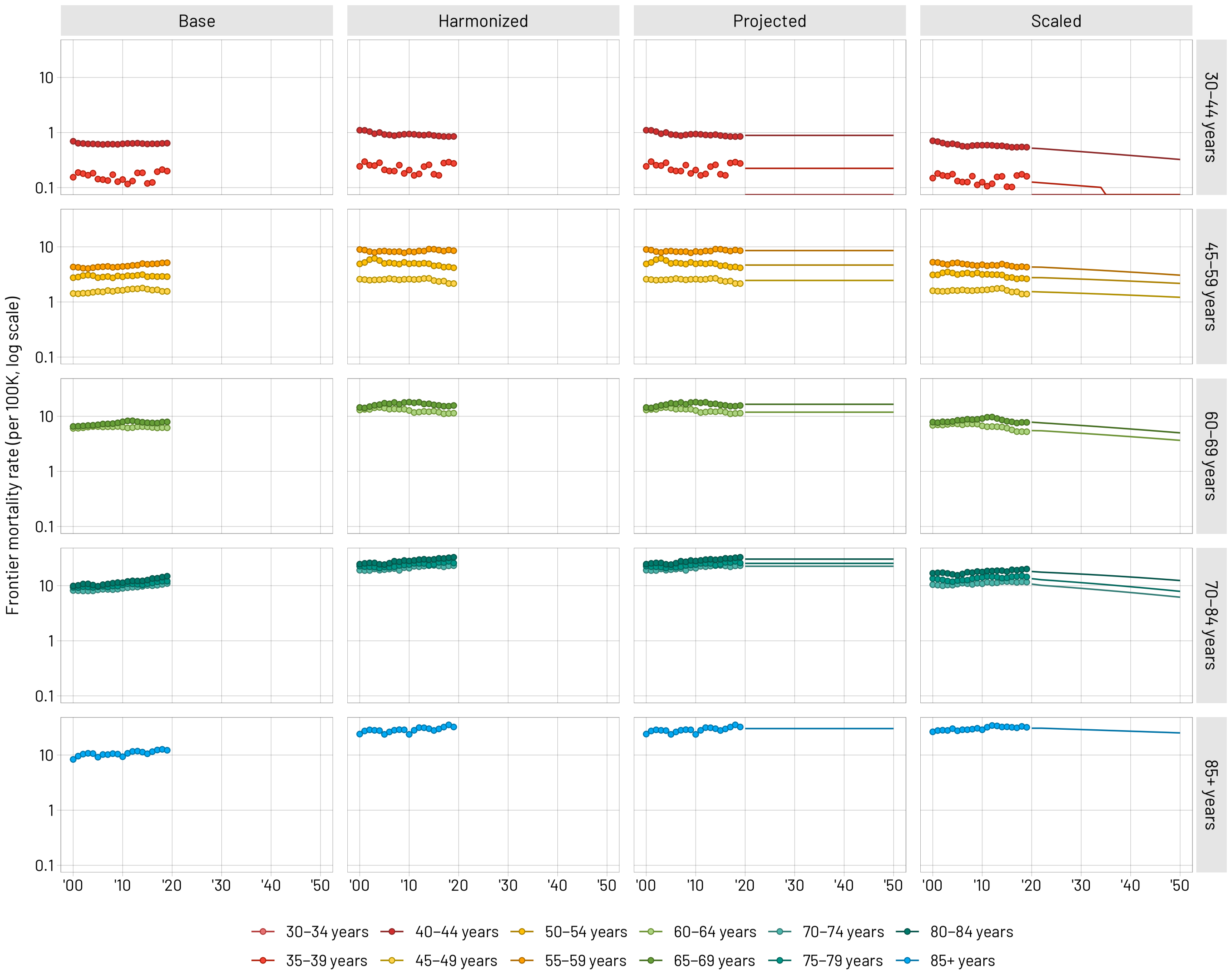

(M) Breast cancer (females)

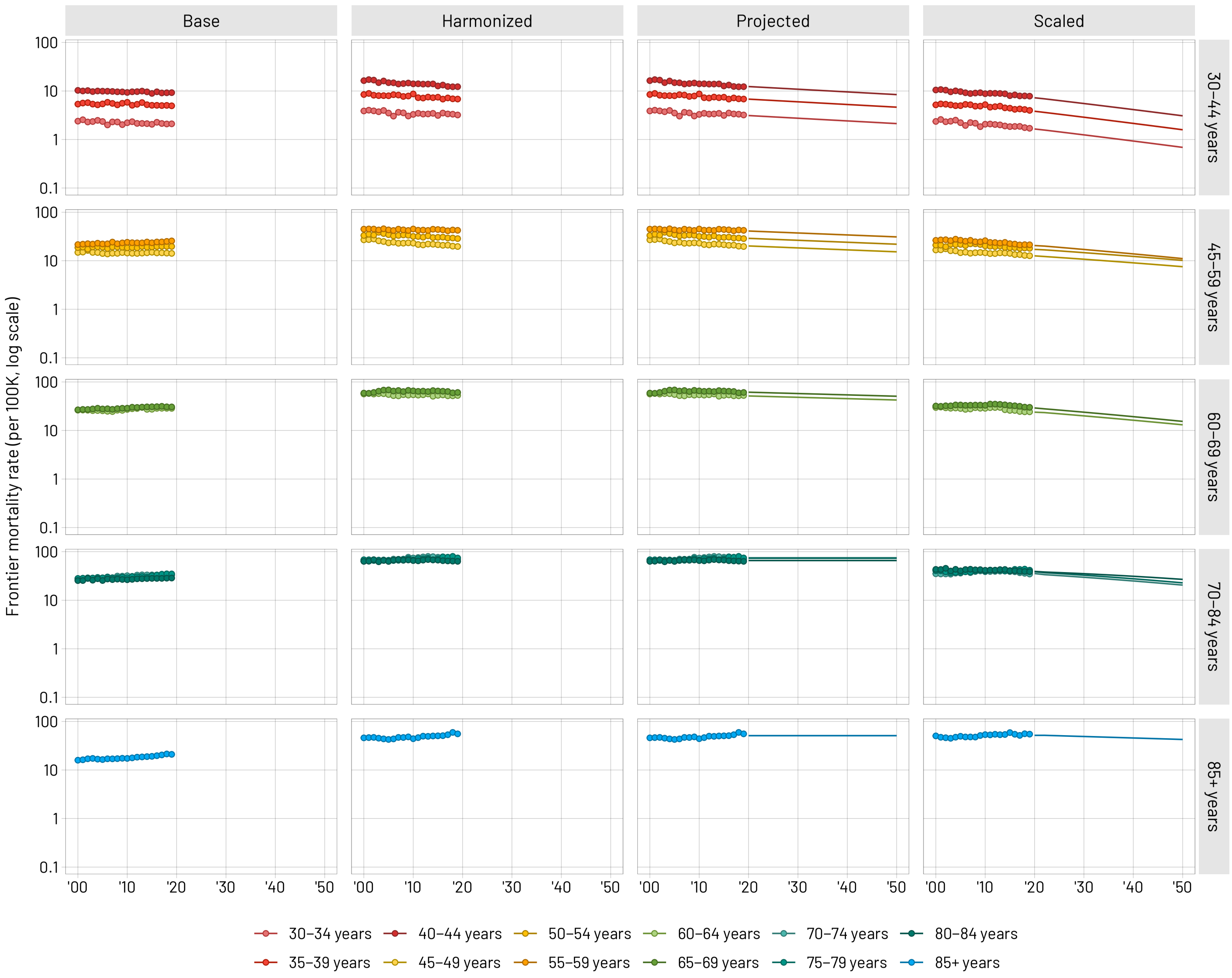

(N) Cervix uteri cancer (females)

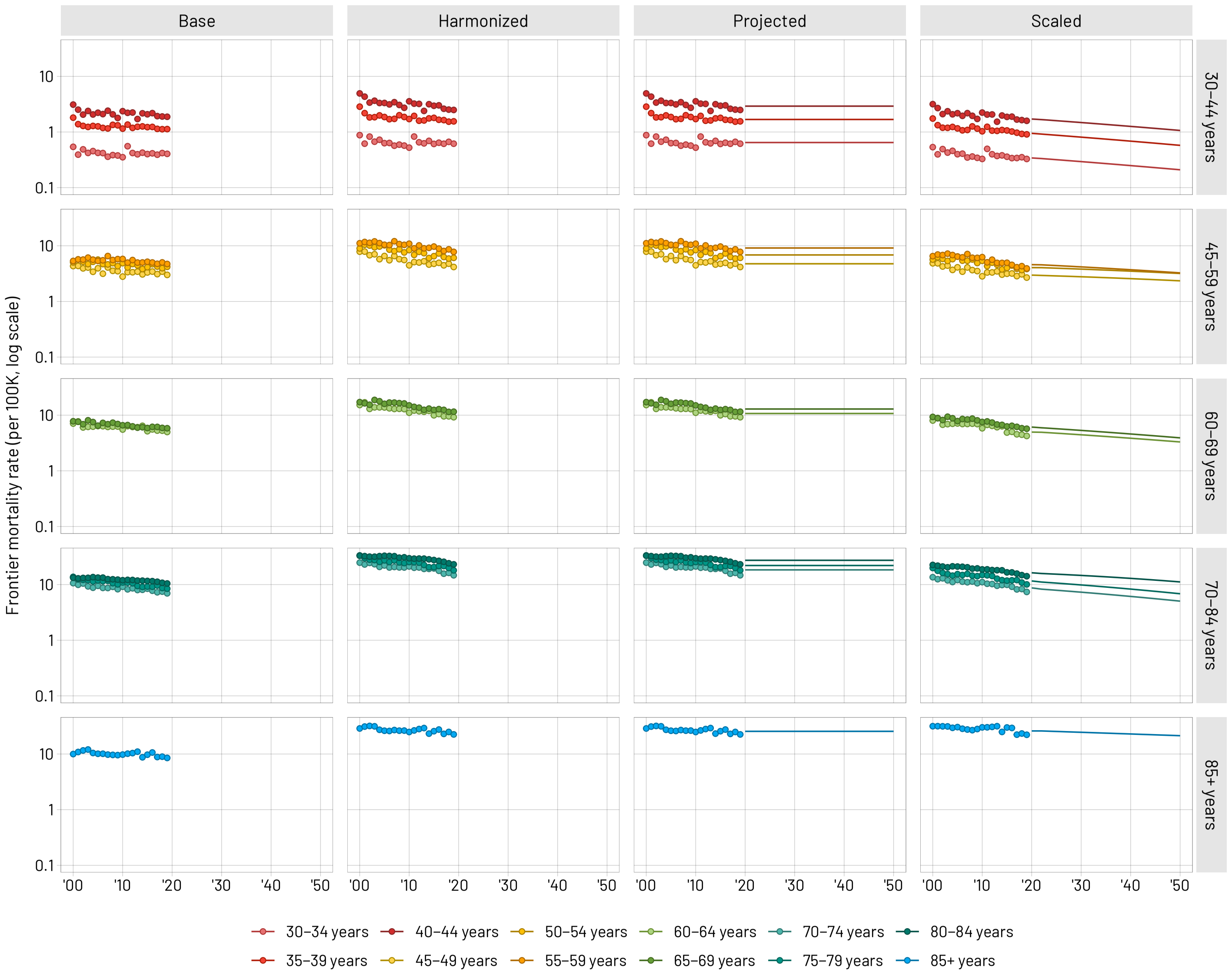

(O) Other malignant neoplasms

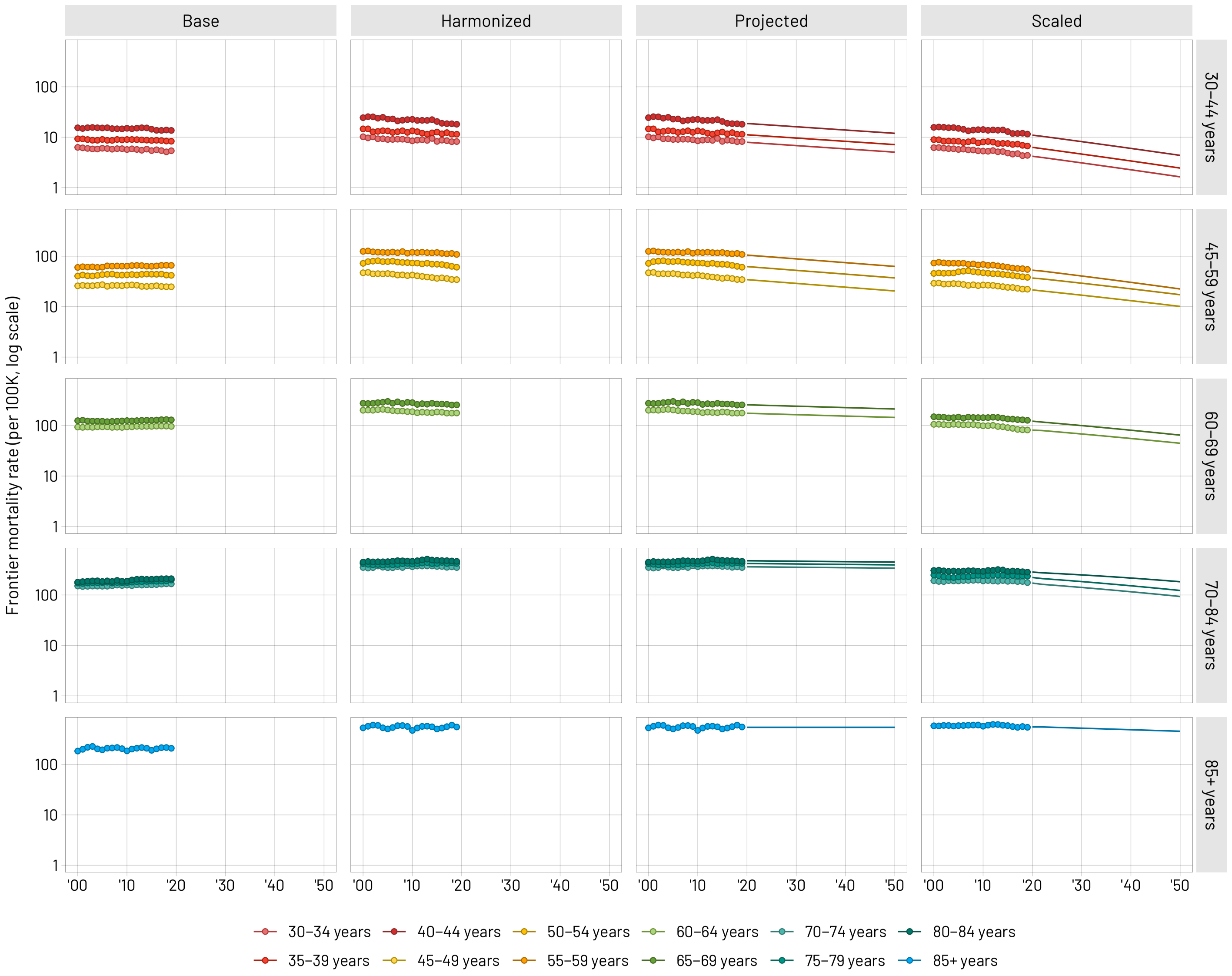

(P) Diabetes mellitus

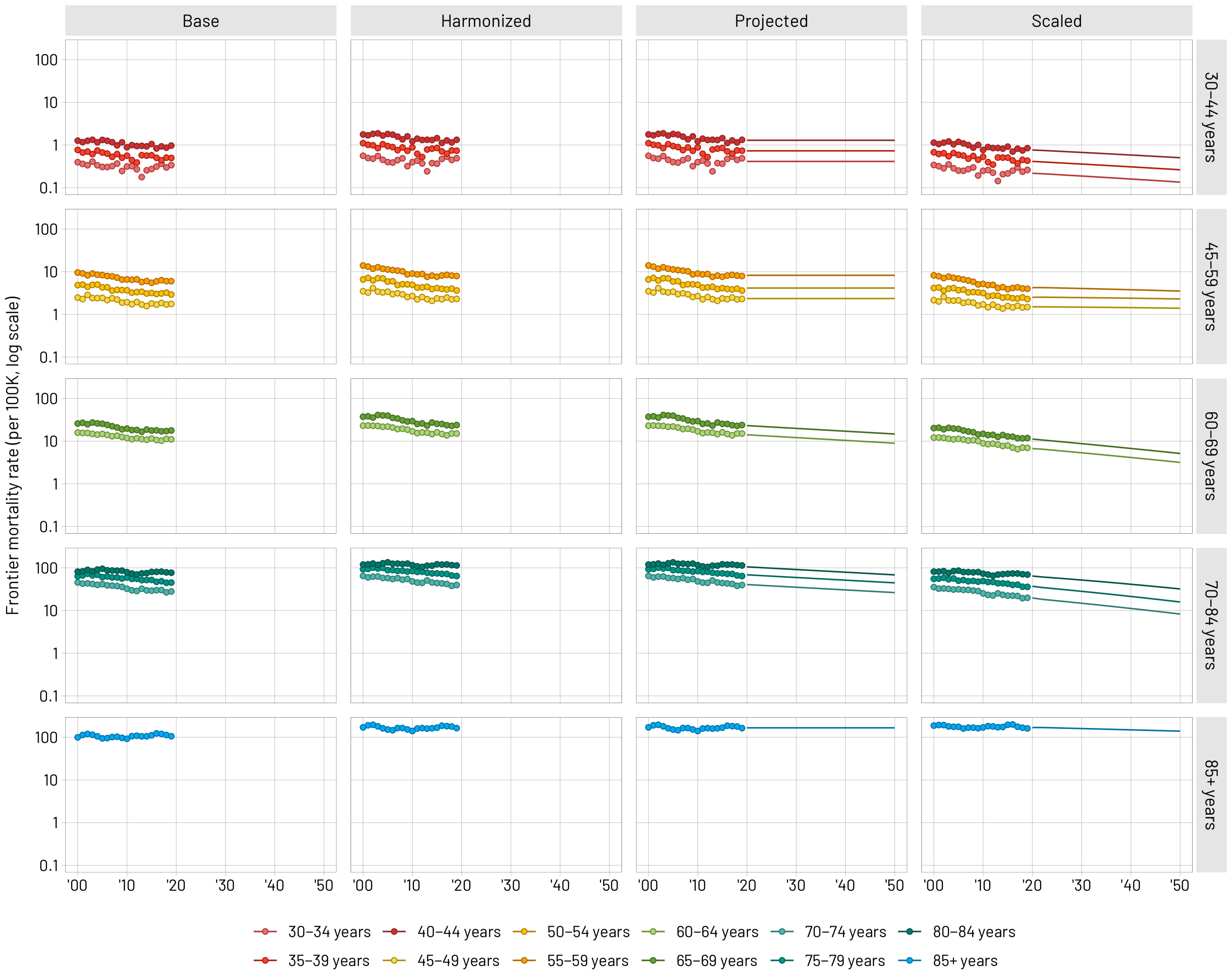

(Q) Cardiovascular diseases

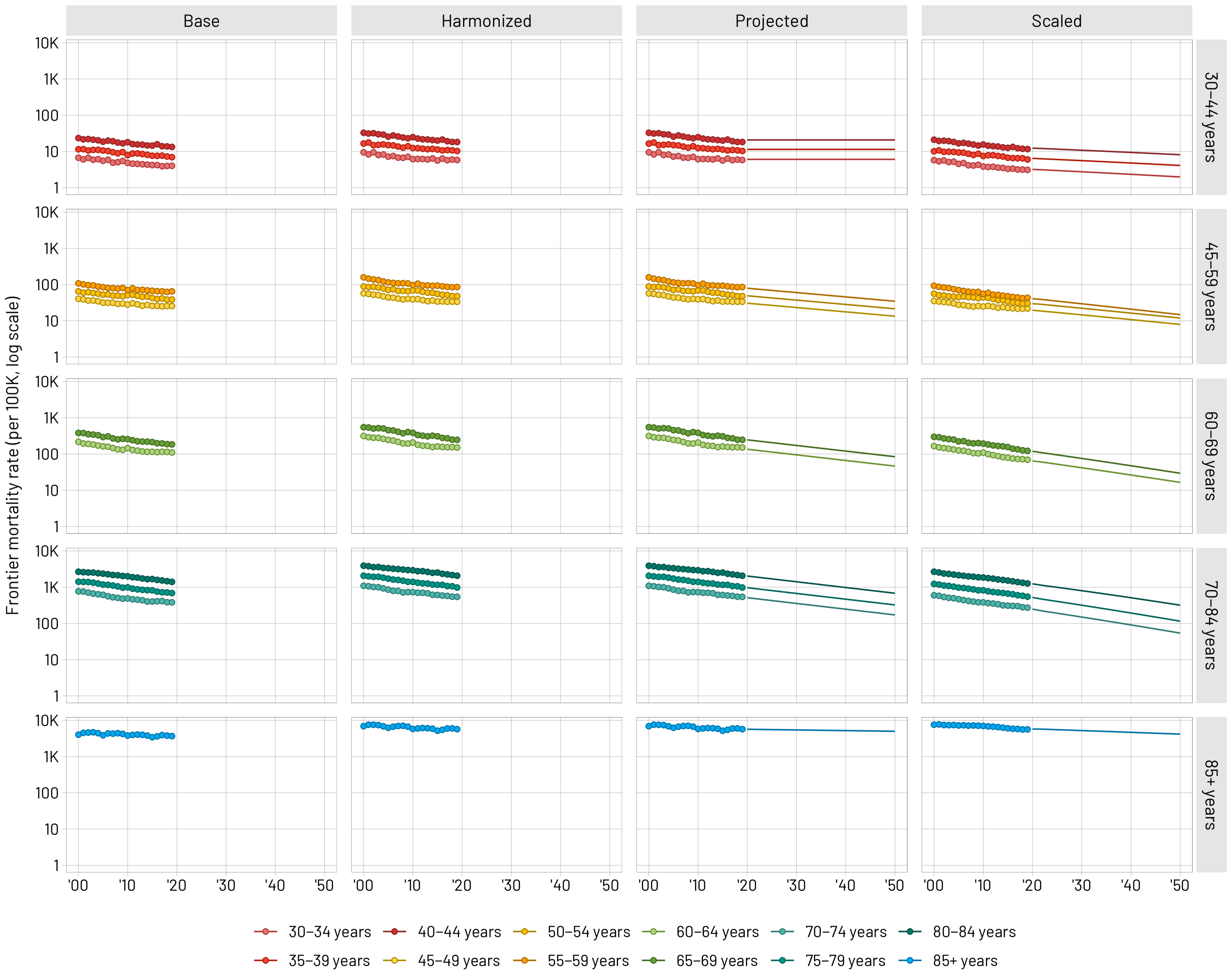

(R) Ischaemic heart disease

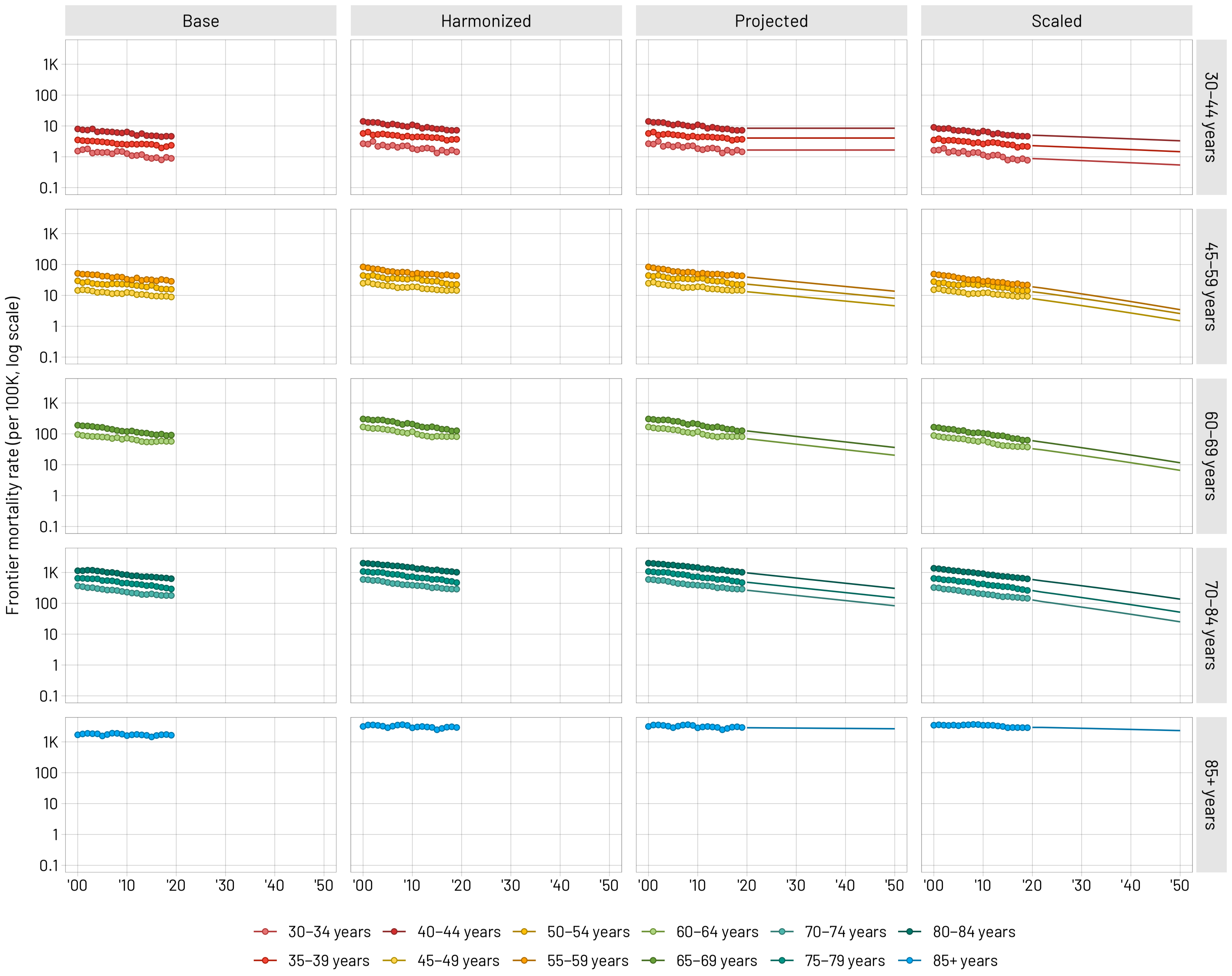

(S) Stroke

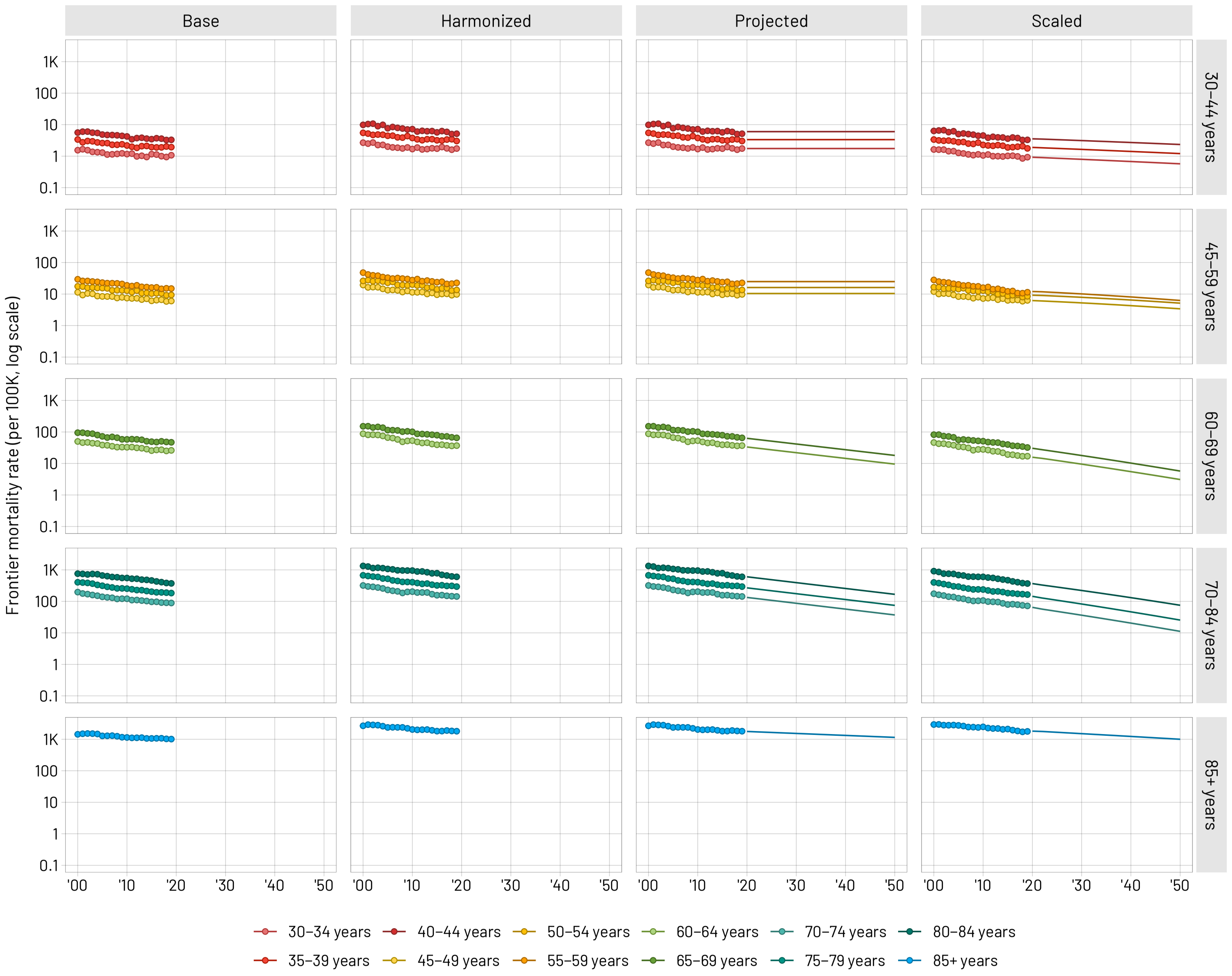
Base
Harmonized
Projected
Scaled
30–44 years
45–59 years
60–69 years
70–84 years
85+ years
Frontier mortality rate (per 100K, log scale)
0.1
1
10
100
1K
'00
'10
'20
'30
'40
'50
30–34 years
35–39 years
40–44 years
45–49 years
50–54 years
55–59 years
60–64 years
65–69 years
70–74 years
75–79 years
80–84 years
85+ years

(T) Other cardiovascular diseases

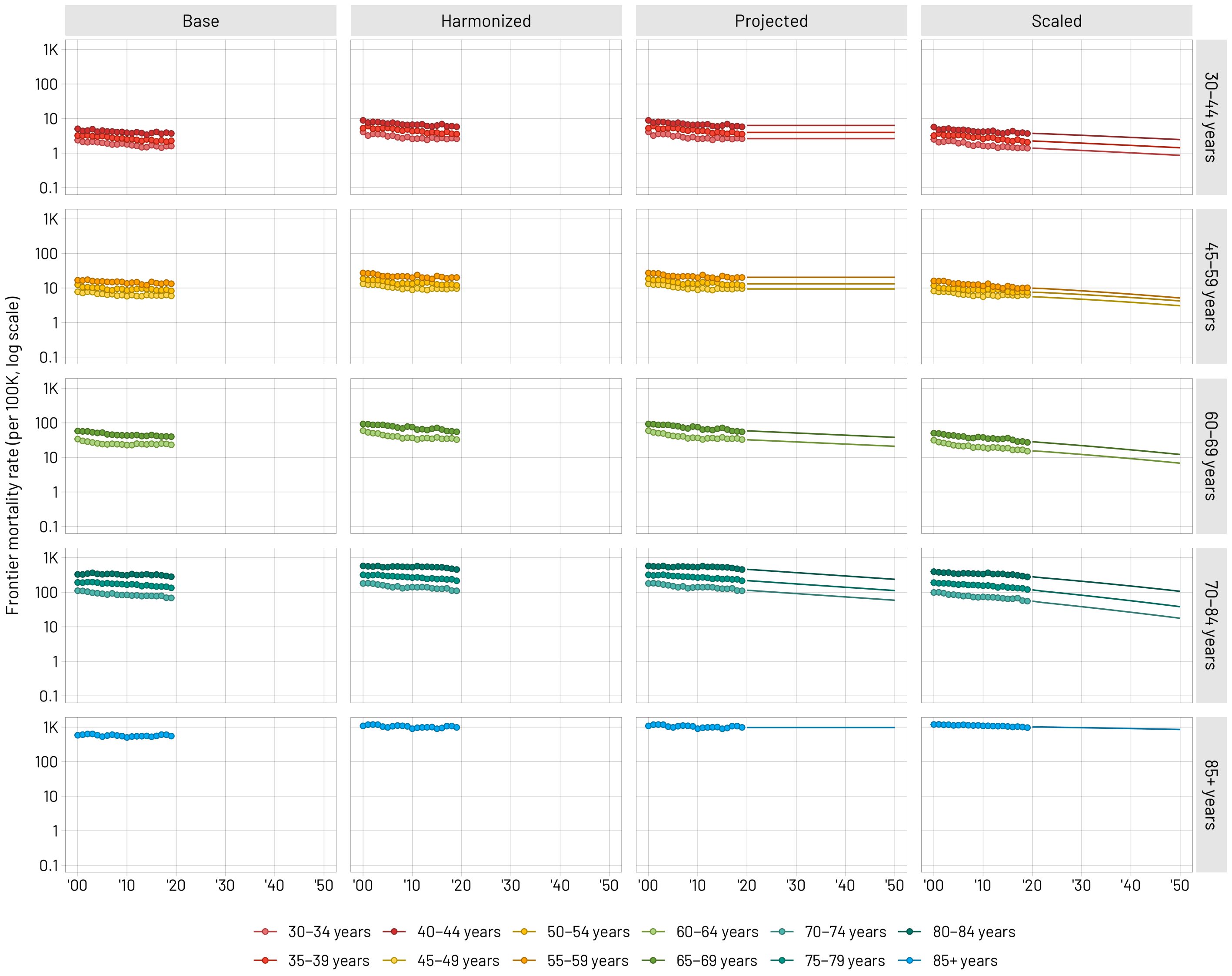

(U) Respiratory diseases

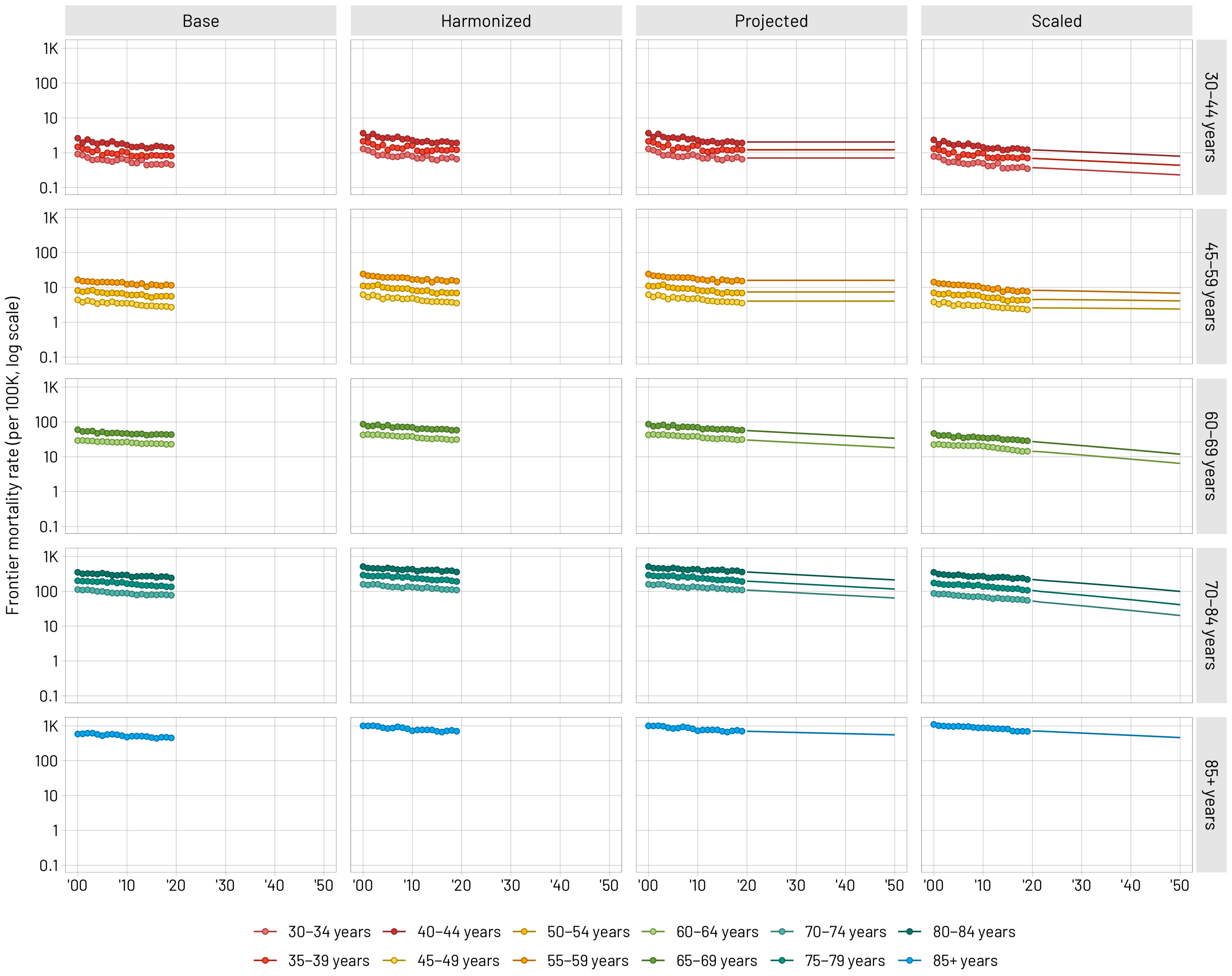

(V) Chronic obstructive pulmonary disease

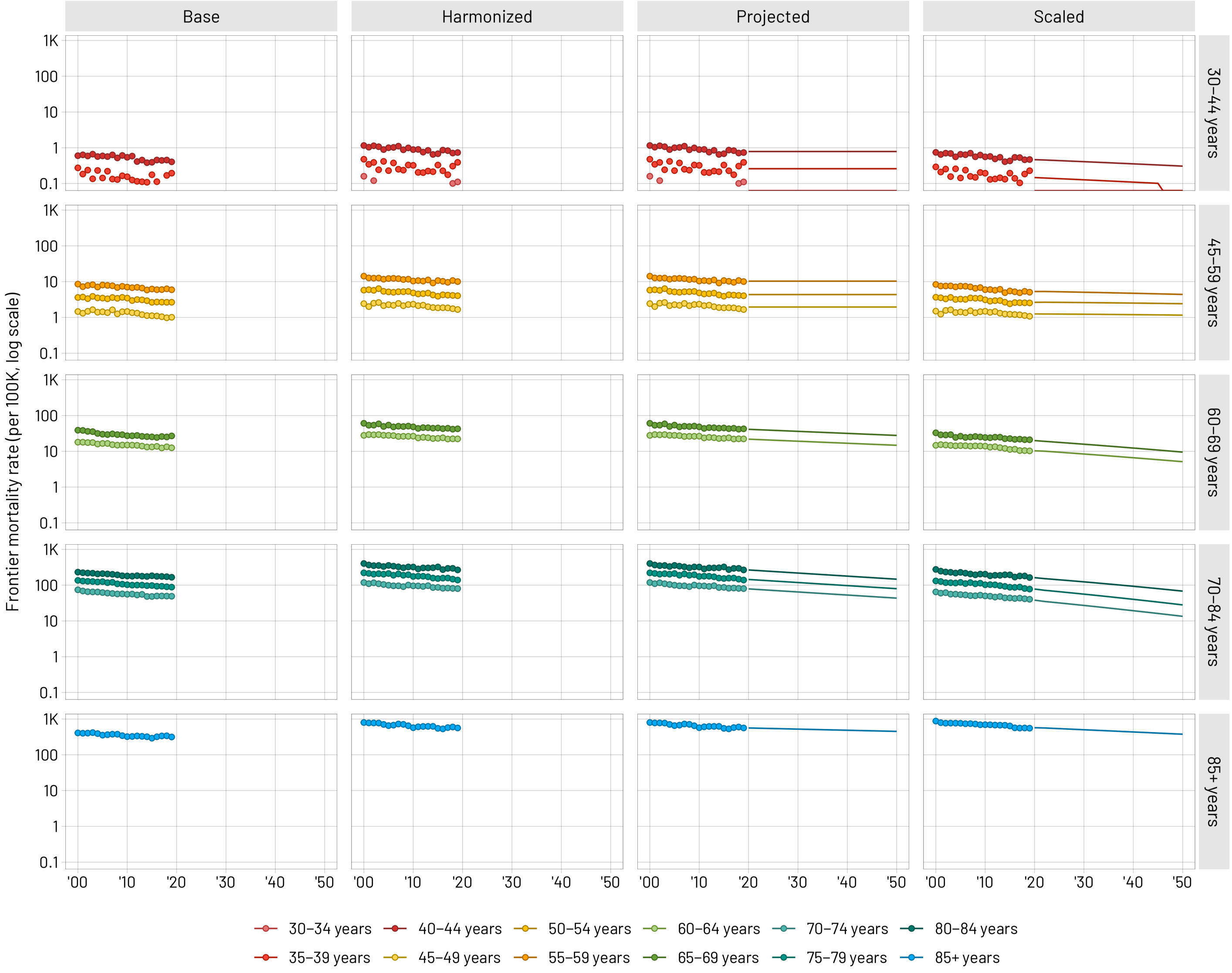

(W) Other respiratory diseases

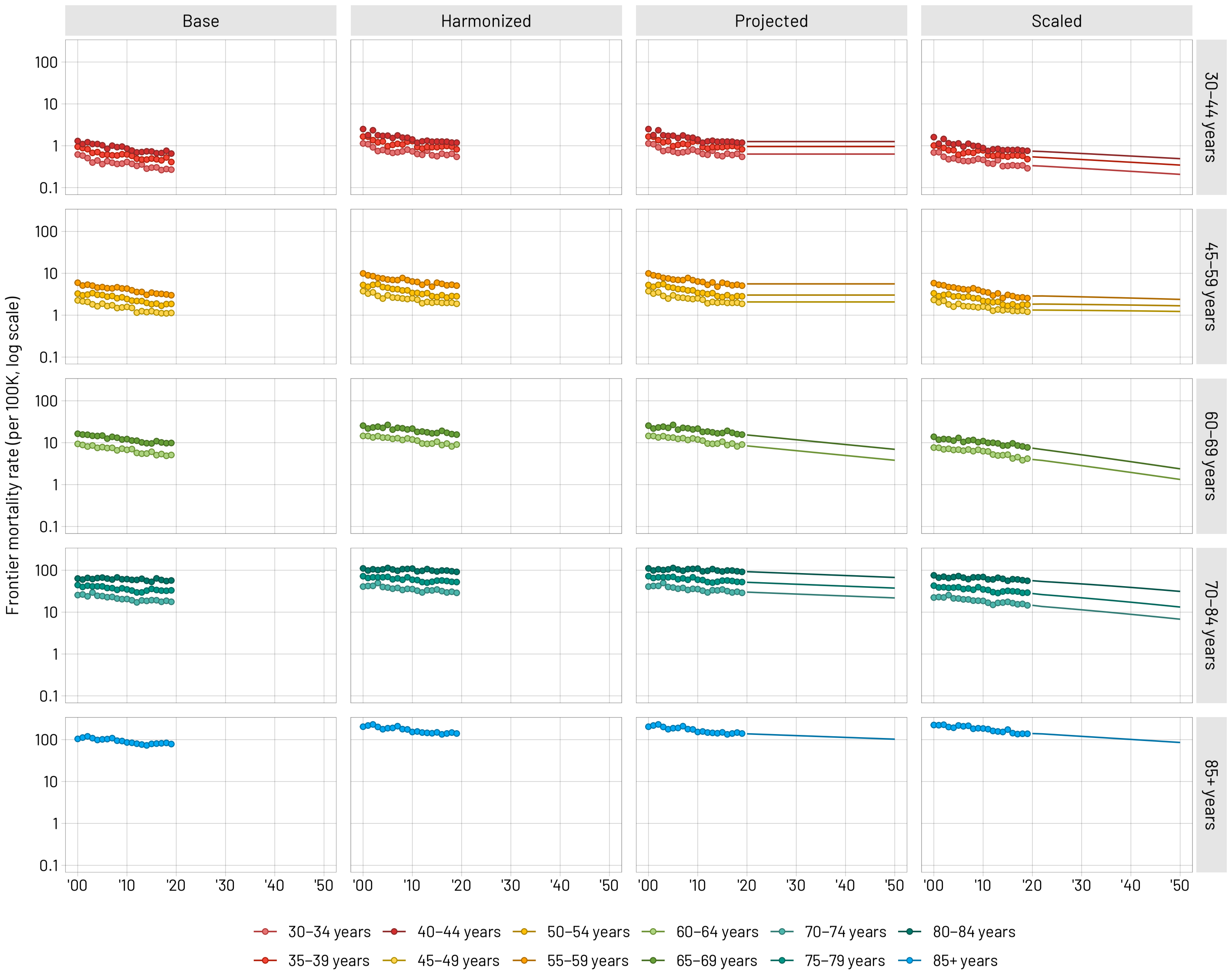

(X) Digestive diseases

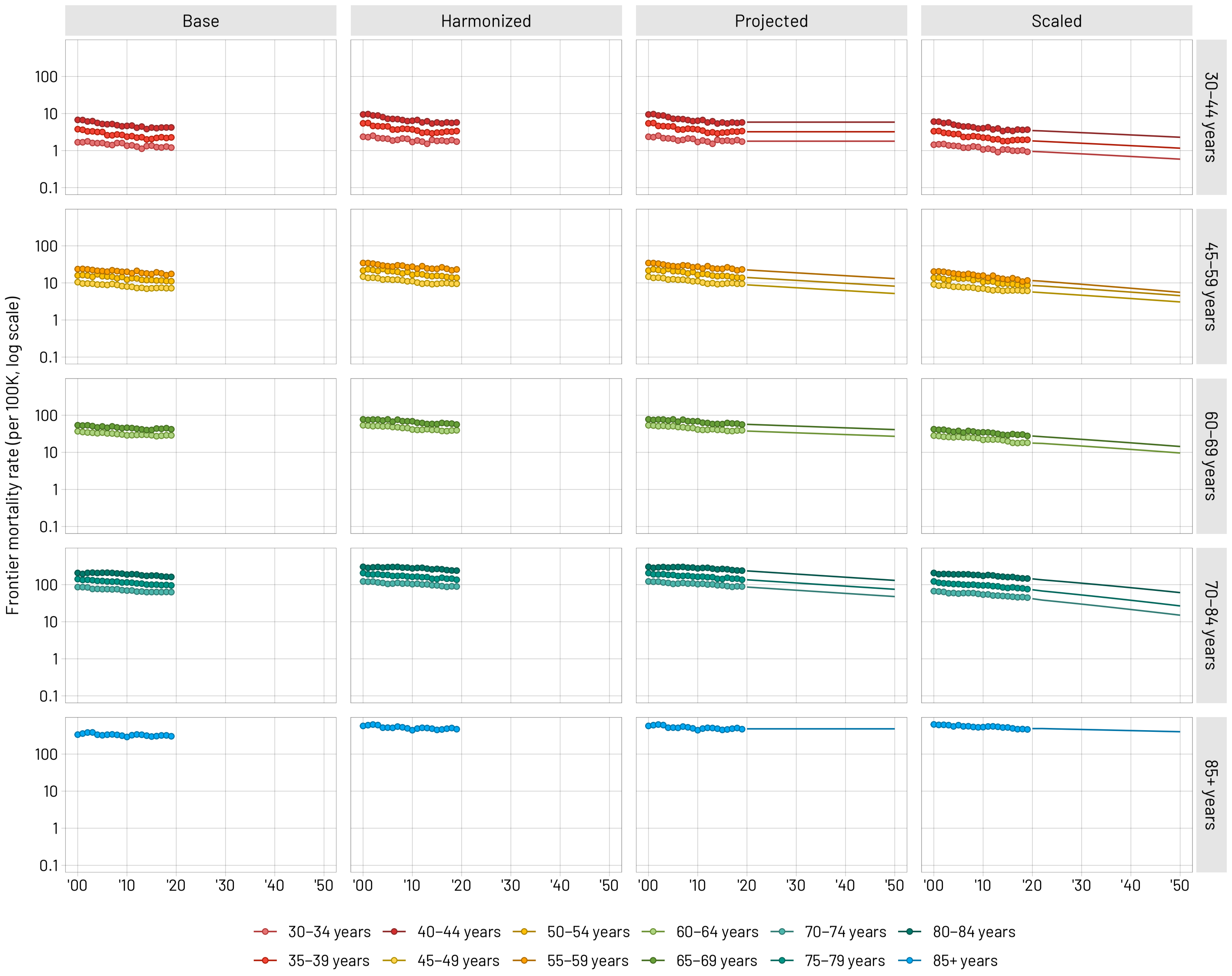

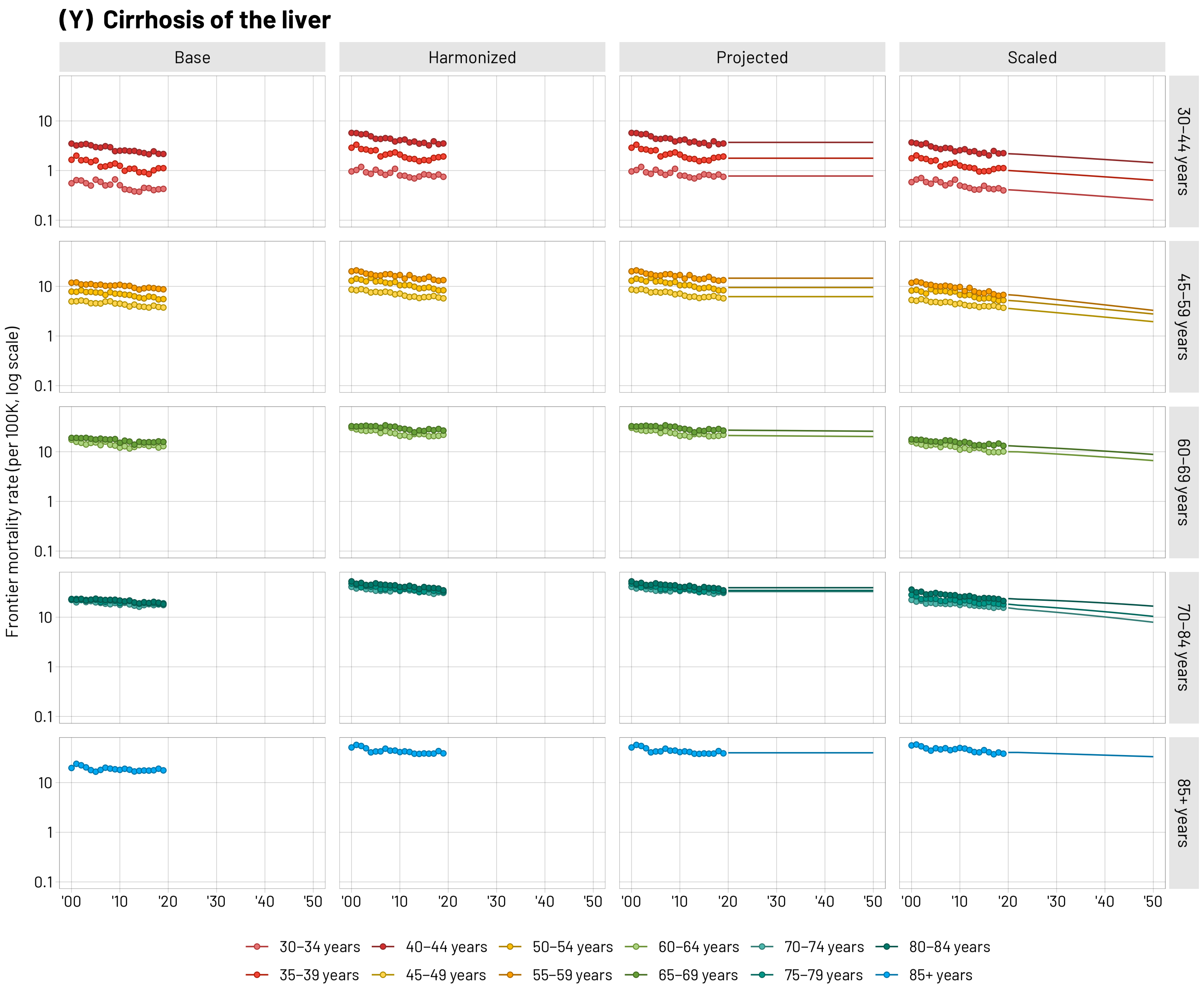
(Y) Cirrhosis of the liver
Base
Harmonized
Projected
Scaled
30–44 years
45–59 years
60–69 years
70–84 years
85+ years
Frontier mortality rate (per 100K, log scale)
0.1
1
10
'00
'10
'20
'30
'40
'50
30–34 years
35–39 years
40–44 years
45–49 years
50–54 years
55–59 years
60–64 years
65–69 years
70–74 years
75–79 years
80–84 years
85+ years

(Z) Other digestive diseases

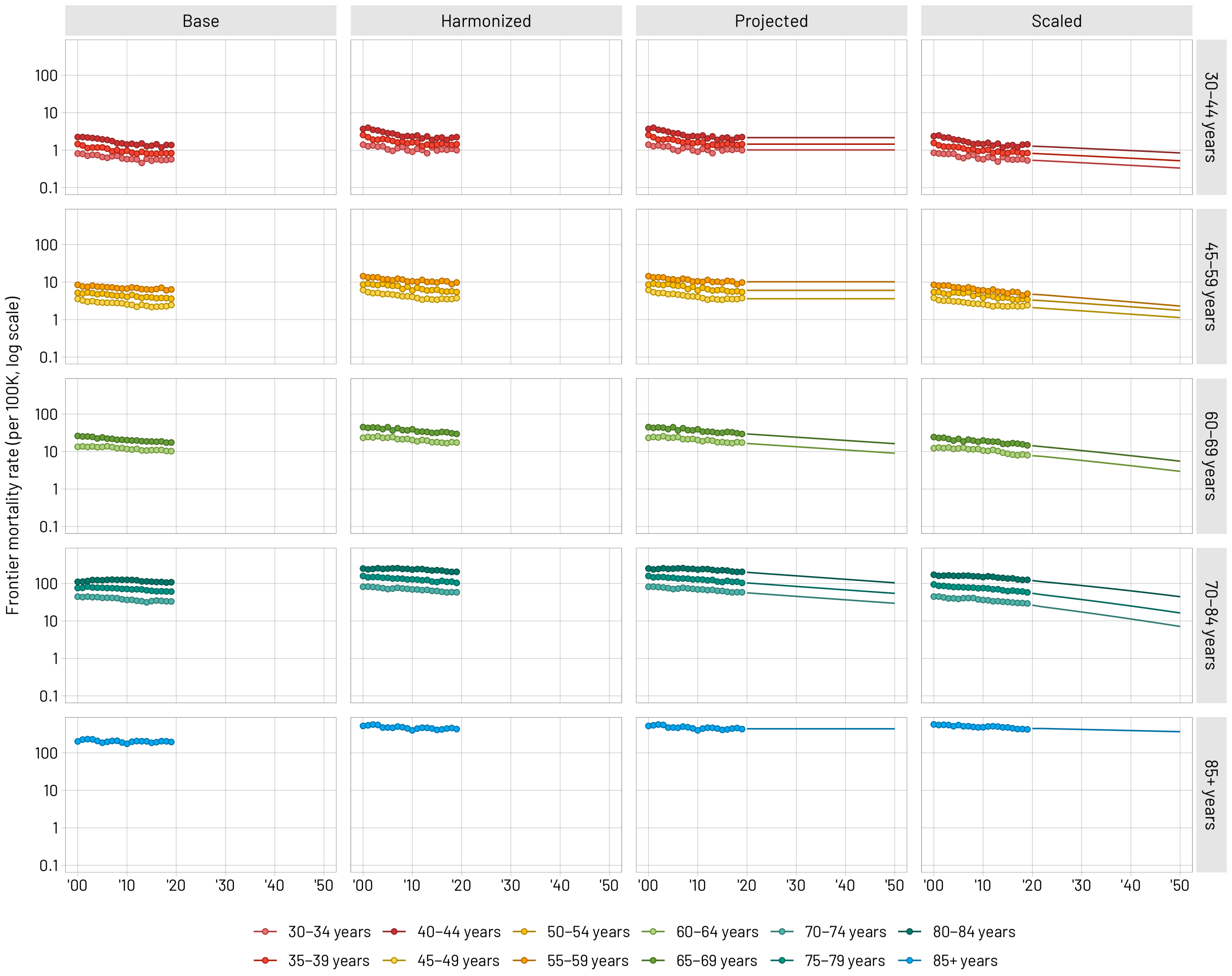

(AA) Other noncommunicable diseases

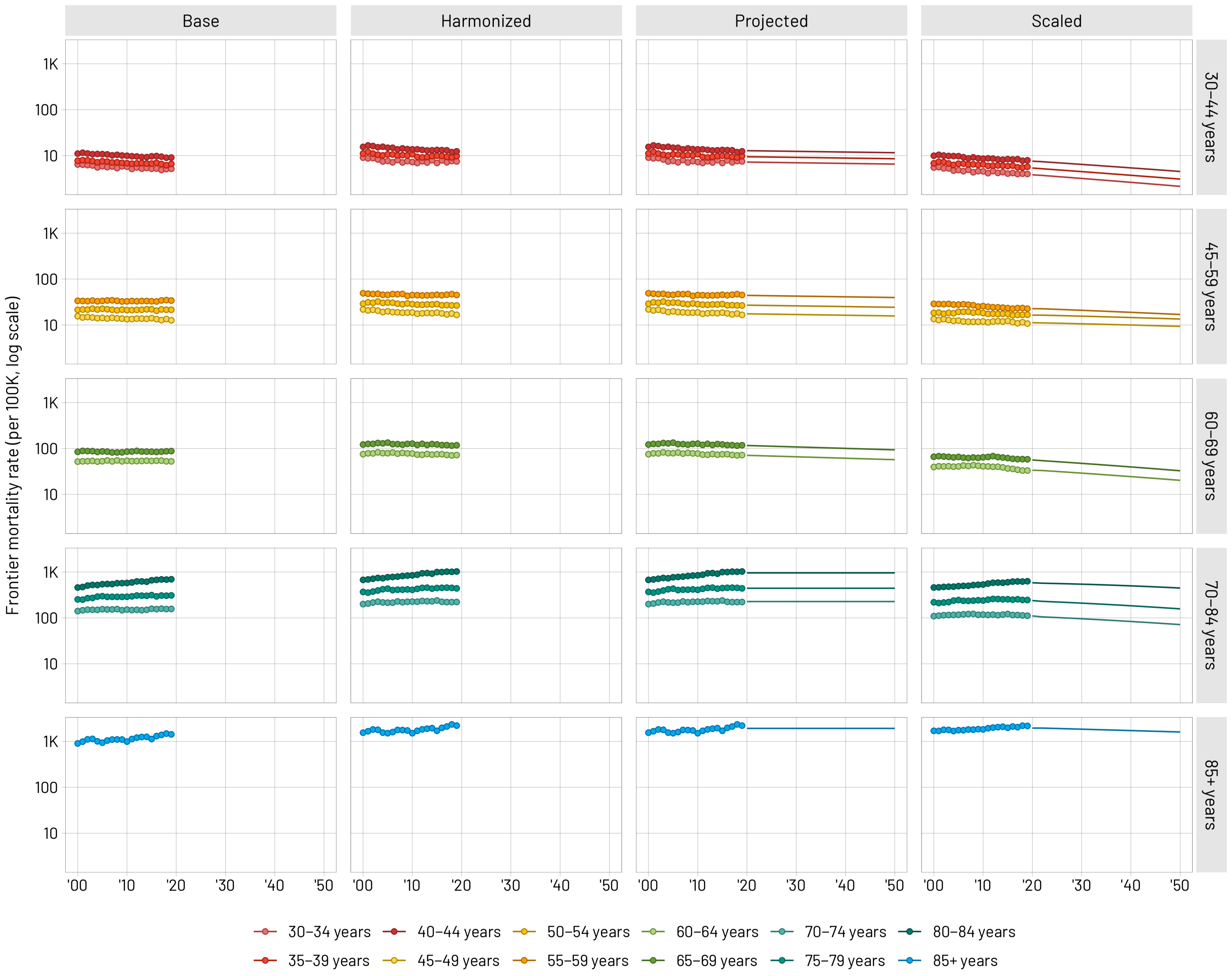

(AB) Injuries

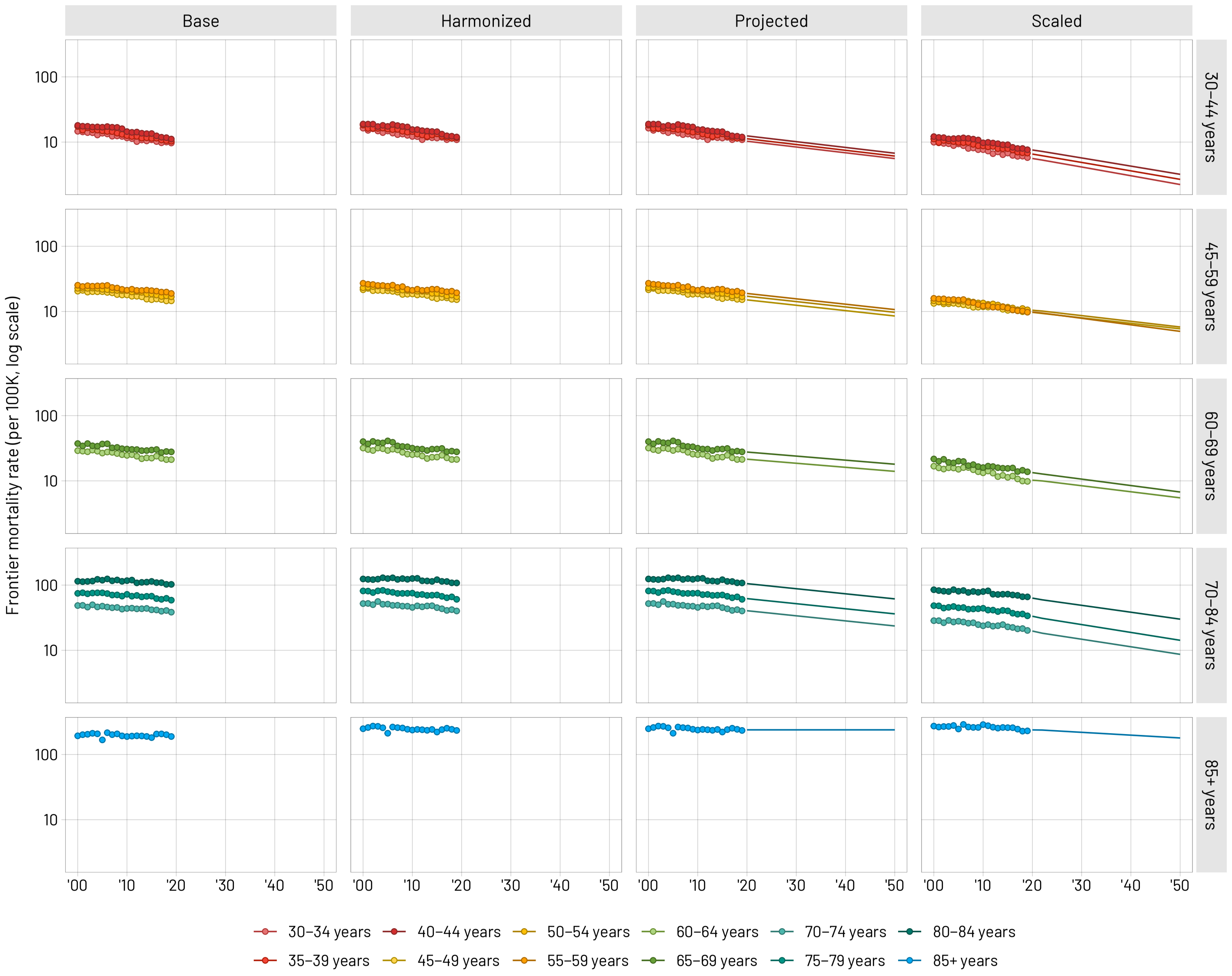

Base
Harmonized
Projected
Scaled
30–44 years
45–59 years
60–69 years
70–84 years
85+ years
Frontier mortality rate (per 100K, log scale)
100
10
'00
'10
'20
'30
'40
'50
30–34 years
35–39 years
40–44 years
45–49 years
50–54 years
55–59 years
60–64 years
65–69 years
70–74 years
75–79 years
80–84 years
85+ years

(AC) Unintentional injuries

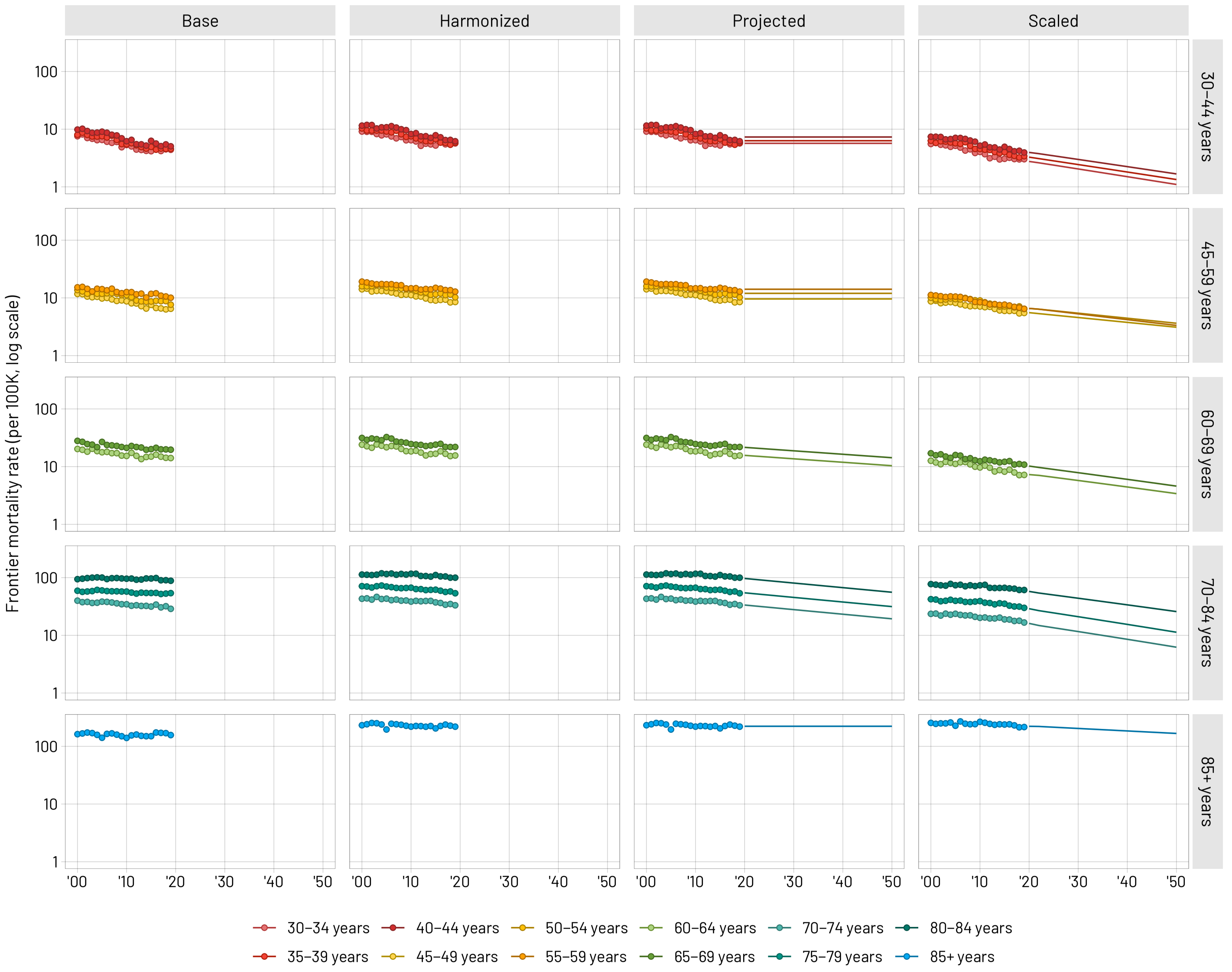

(AD) Road injury

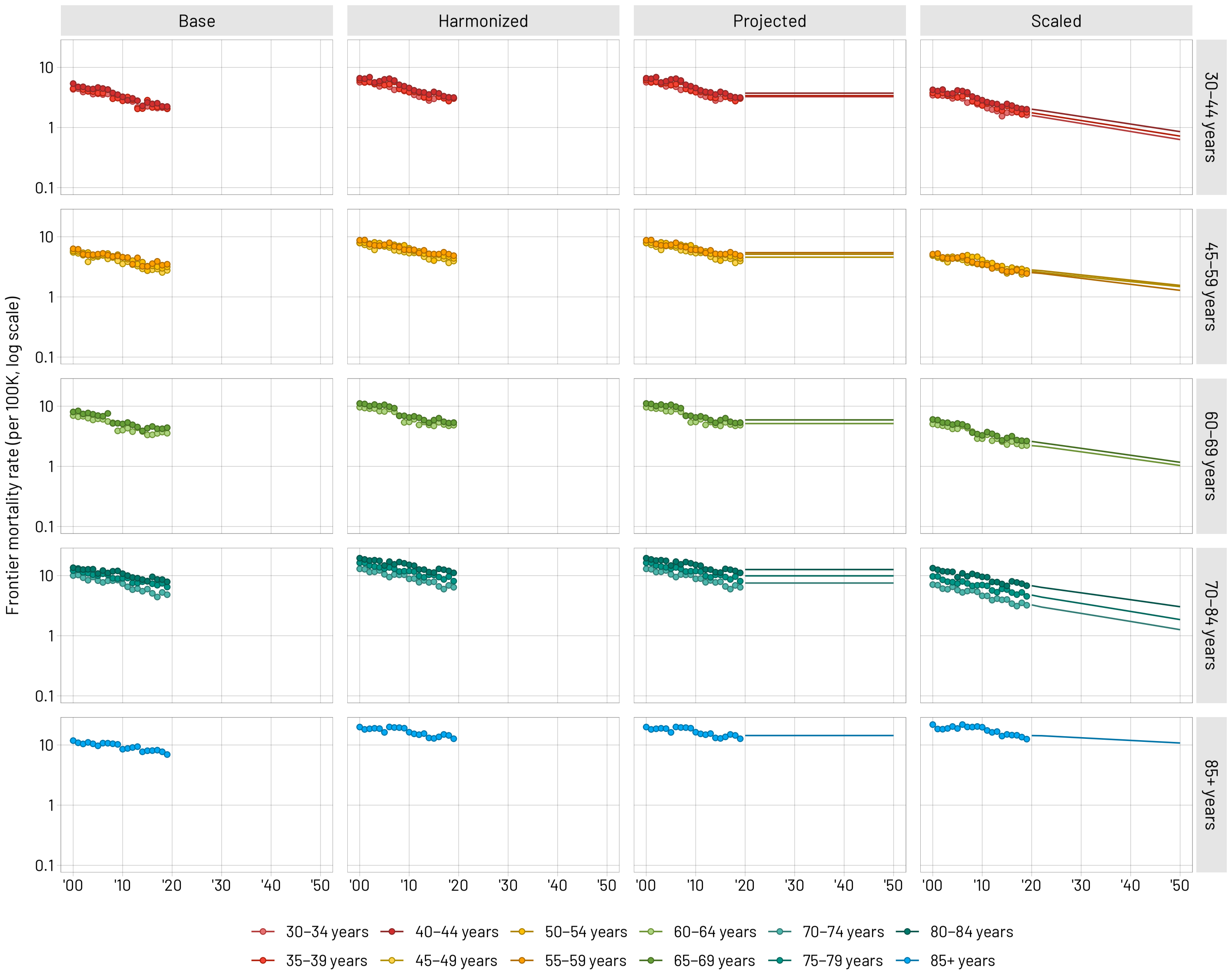

(AE) Other unintentional injuries

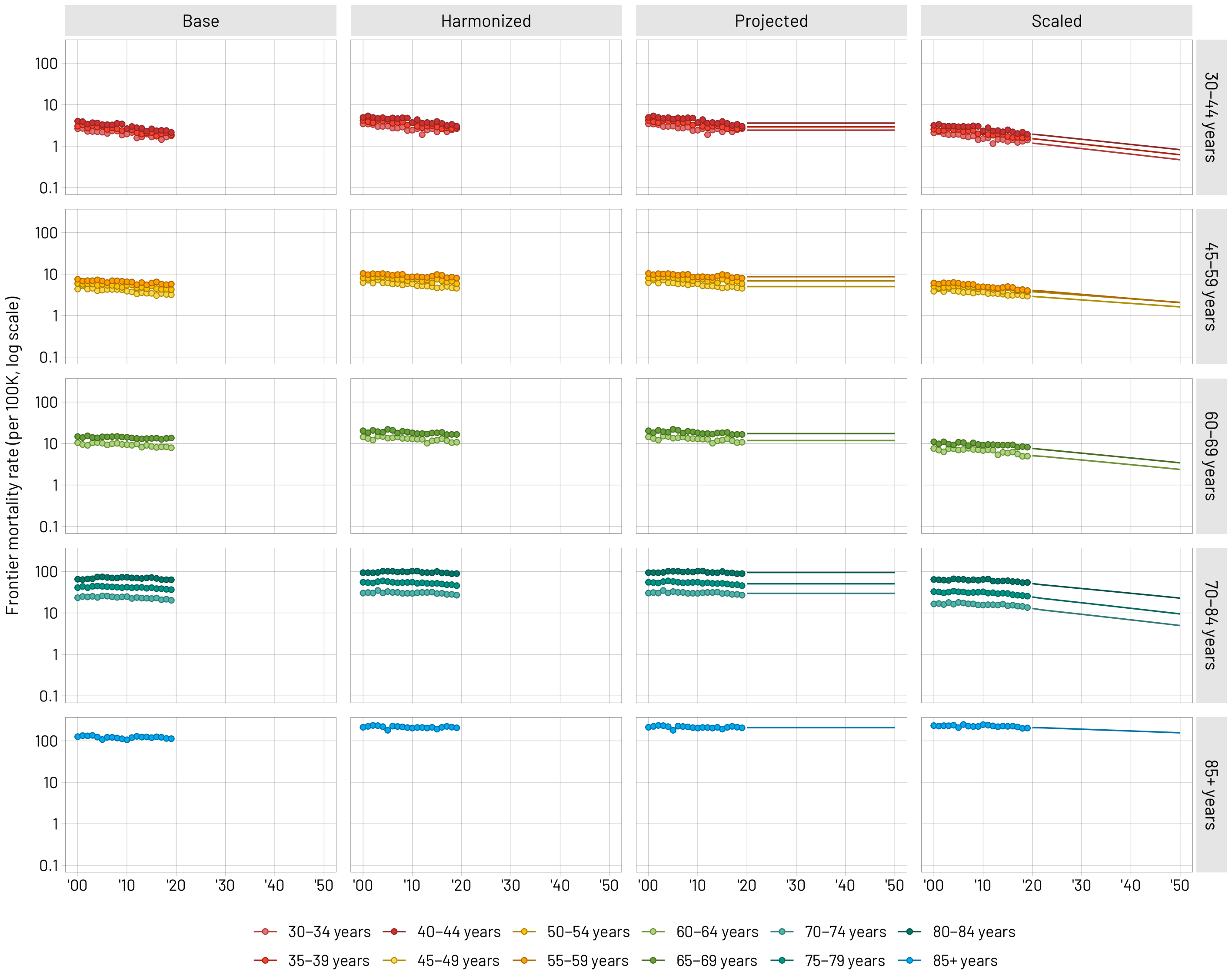

(AF) Intentional injuries

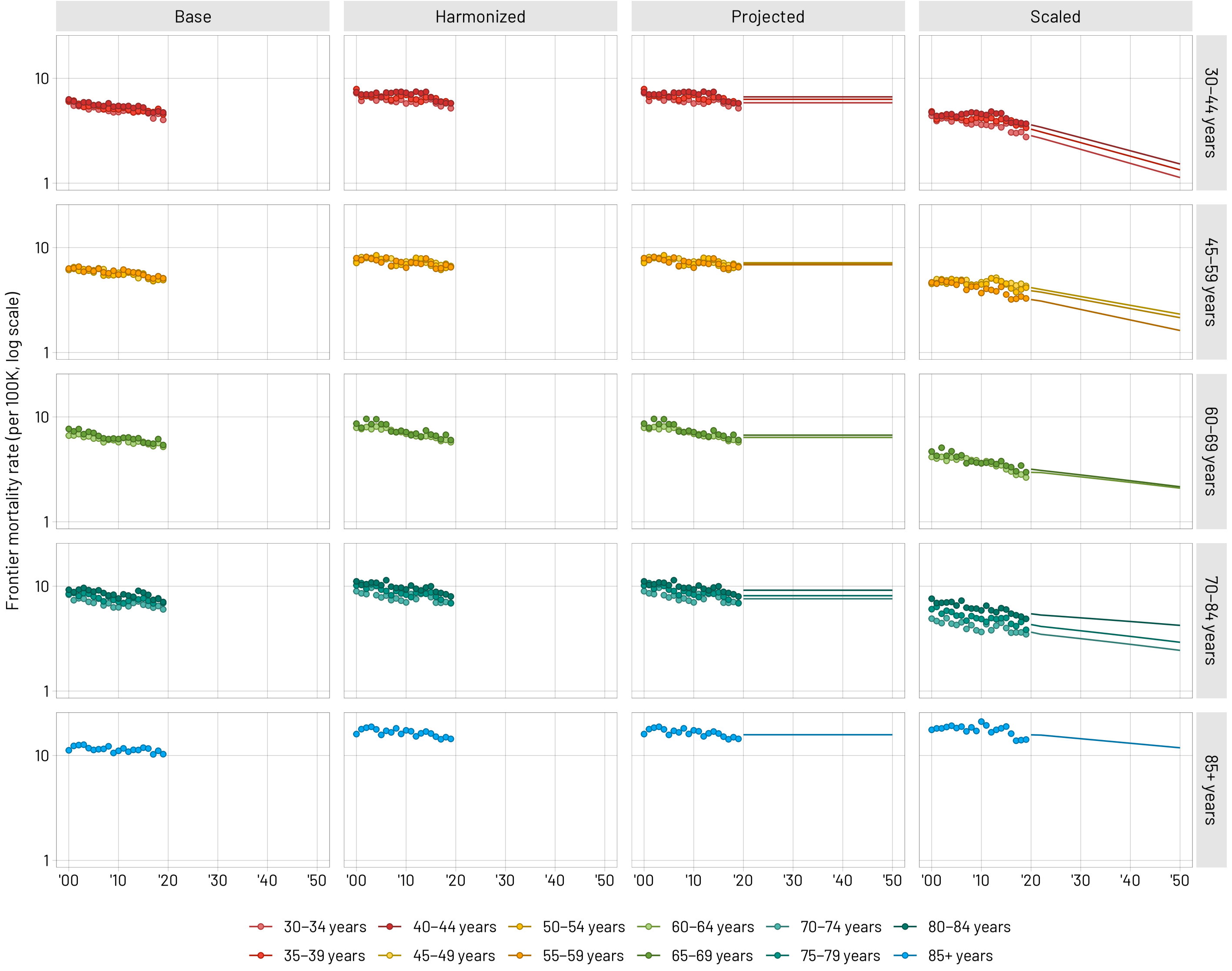

3. Estimating the economic value associated with reducing cause-specific avoidable mortality

We denote $m(x)$ the mortality rate among the age group $[x; x+u)$, where u is the size of the age group ($u = 5$, except for 0-1, 1-4, and 85+ age groups). The probability of dying at age a, $q(a)$, is expressed as $q(a) \sim 1 - e^{-u\sum_{x=0}^{a} m(x)}$, when using a discrete formulation.

We compare a country's age group's probability of dying (C for country) to a reference probability of dying ($\mathcal{F}$ for frontier), that is $q_C(a)$ to $q_{\mathcal{F}}(a)$. The difference in those probabilities can be obtained by the following "avoidable mortality": $\delta_{C,\mathcal{F}}(a) = q_C(a) - q_{\mathcal{F}}(a)$ (in what follows, we examine differentials over one-year periods, denoted $\delta_{C,\mathcal{F},1}$, where u is then set to 1 year).

Now take the population of country C with an age distribution $\alpha_C(a)$ ($\alpha_C(a) = \frac{P_C(a)}{\sum_{a=0}^{\omega} P_C(a)}$, where $P_C(a)$ is the size of the age group $[a; a+u)$ and ω is the oldest age group). In addition, denote v the monetary value assigned to the change in the probability of dying ($\delta_{C,\mathcal{F},1}$ over one year) at a given age a. Then, for a given year, the aggregate monetary value associated with avoidable mortality (comparing C and $\mathcal{F}$) is given by:

$$V_{C,\mathcal{F}} \sim \sum_{a=0}^{\omega} \alpha_C(a) v(\delta_{C,\mathcal{F},1}(a)) \,. \qquad [2]$$

We then assign a value to $v(\delta_{C,\mathcal{F},1}(a))$ that depends on the extent of the differential in mortality risk $\delta_{C,\mathcal{F},1}$ and on the VSL in country C (VSL_C). We apply the methods developed by Chang and colleagues[23], where v increases with $\delta_{C,\mathcal{F},1}$ and its marginal rate of increase decreases with $\delta_{C,\mathcal{F},1}$ (consistent with Cardoso & Dahis (2024)[40] and Hammitt (2020)[33]). The value of VSL_C can be related to the VSL in the US (VSL_{US}) using an income elasticity ε: $VSL_C = VSL_{US} * (\frac{Y_C}{Y_{US}})^{\varepsilon}$, where Y_C and Y_{US} are the gross national income (GNI) per capita of C and the United States, respectively.

To derive VSL_C, we set $\varepsilon = 0.8$ for countries with GNI per capita greater than the US and $\varepsilon = 1.2$ for countries with lower GNI per capita (Viscusi & Masterman 2017[45]; Robinson, Hammitt, & O'Keeffe 2019[15]) (alternative values of income elasticity of 1.0 and 1.5 are tested in sensitivity analyses). We also set a floor constraint for the ratio between VSL and GNI per capita of 20. GNI per capita was expressed in current international dollars (Purchasing Power Parity (PPP); World Bank 2022[46]) for the period 2000-2020; and were projected over 2021-2050 using OECD's projected country-specific growth rates over 2021-2050 for listed countries (OECD and G20 countries), and the world average growth rate during the same time period for all remaining countries. We set VSL_{US} = 160 times US GNI per capita (Robinson, Hammitt, & O'Keeffe 2019[15]); and a discount rate of 3% per year.

Therefore, per year and country, we computed the following proportions per cause K and age group a: $p_{C,K}(a) = \frac{\delta_{C,\mathcal{F},K,1}(a)}{\sum_K \delta_{C,\mathcal{F},K,1}(a)}$. We then derived the following value (per year): $V_{C,\mathcal{F},K} \sim \sum_{a=0}^{\omega} \alpha_C(a) v(\delta_{C,\mathcal{F},1}(a)) p_{C,K}(a)$, aggregated per region R:

$$V_{R,K,\mathcal{F}} \sim \sum_{C \in R} w_C V_{C,K,\mathcal{F}} = \sum_{C \in R} \sum_{a=0}^{\omega} w_C \alpha_C(a) v(\delta_{C,\mathcal{F},1}(a)) p_{C,K}(a). \qquad [3]$$

From [3], we obtained an economic value directly comparable to annual incomes, which captures the percent of income an individual would be willing to forgo to live one year under the lowest possible mortality rate for a given cause of death (in the region R).

Supplementary Information 2 – Detailed Results

In this document, we fully display the results from the estimation of the economic value associated with reducing avoidable mortality by cause of death and country, over the time period 2000-2050.

1. Economic value of reducing avoidable mortality

Table 4 collects the estimated economic values of reducing avoidable mortality for each cause of death, each region, for both females and males, for the years 2000, 2019 and 2050, in terms of percent of annual income. Figures 3 and 4 display the economic values of reducing avoidable mortality for the alternative levels of causes of death 1 and 3, for each region, for both females and males. Figures 5, 6, and 7 display the distribution of the economic value of reducing avoidable mortality by cause of death, region, for both females and males, for either level 1, level 2, or level 3 causes of death.

Table 4. Economic values of reducing avoidable mortality

Economic values of reducing avoidable mortality, measured as percent of annual income, assigned to causes of death for the six regions and the world, in the years 2000, 2019, and 2050; females and males.

Level	Cause of death	2000			2019			2050		
		Females	Males	Total	Females	Males	Total	Females	Males	Total
**China**										
1	Communicable, maternal, perinatal and nutritional conditions	2.4 %	2.6 %	2.5 %	0.8 %	1.1 %	0.9 %	0.1 %	0.2 %	0.2 %
2	Infectious and parasitic diseases	1.6 %	1.9 %	1.8 %	0.5 %	0.8 %	0.7 %	0.0 %	0.1 %	0.1 %
2	Maternal and neonatal conditions	0.7 %	0.6 %	0.6 %	0.3 %	0.2 %	0.3 %	0.1 %	0.1 %	0.1 %
2	Nutritional deficiencies	0.0 %	0.0 %	0.0 %	0.0 %	0.0 %	0.0 %	0.2 %	0.1 %	0.1 %
1	Noncommunicable diseases	8.9 %	11.9 %	10.5 %	11.7 %	17.9 %	14.9 %	21.4 %	25.8 %	23.6 %
2	Cardiovascular diseases	3.3 %	4.1 %	3.7 %	5.9 %	8.0 %	7.0 %	11.8 %	13.1 %	12.4 %
3	Ischaemic heart disease	0.3 %	0.9 %	0.6 %	2.0 %	3.1 %	2.5 %	6.3 %	6.2 %	6.2 %
3	Stroke	2.6 %	2.8 %	2.7 %	3.5 %	4.2 %	3.9 %	3.8 %	5.0 %	4.4 %
3	Other cardiovascular diseases	0.5 %	0.3 %	0.4 %	0.4 %	0.5 %	0.4 %	1.7 %	1.6 %	1.7 %
2	Diabetes mellitus	0.2 %	0.1 %	0.1 %	0.3 %	0.2 %	0.3 %	0.6 %	0.6 %	0.6 %
2	Digestive diseases	0.2 %	0.8 %	0.5 %	0.0 %	0.7 %	0.4 %	0.0 %	0.2 %	0.1 %
3	Cirrhosis of the liver	0.2 %	0.6 %	0.4 %	0.1 %	0.6 %	0.3 %	0.0 %	0.2 %	0.1 %
3	Other digestive diseases	0.1 %	0.2 %	0.1 %	0.0 %	0.1 %	0.1 %	0.0 %	0.0 %	0.0 %
2	Malignant neoplasms	2.7 %	4.4 %	3.5 %	3.8 %	6.8 %	5.3 %	6.4 %	10.0 %	8.2 %
3	Breast cancer	0.0 %	0.0 %	0.0 %	0.1 %	0.0 %	0.1 %	0.6 %	0.0 %	0.3 %
3	Cervix uteri cancer	0.1 %	0.0 %	0.1 %	0.4 %	0.0 %	0.2 %	0.5 %	0.0 %	0.3 %
3	Liver cancer	0.4 %	1.2 %	0.8 %	0.2 %	0.8 %	0.5 %	0.4 %	1.2 %	0.8 %
3	Mouth and oropharynx cancers	0.1 %	0.1 %	0.1 %	0.1 %	0.2 %	0.1 %	0.0 %	0.3 %	0.1 %
3	Oesophagus cancer	0.4 %	0.7 %	0.5 %	0.3 %	0.9 %	0.6 %	0.2 %	0.7 %	0.4 %
3	Stomach cancer	0.6 %	1.0 %	0.8 %	0.7 %	1.4 %	1.0 %	0.2 %	0.8 %	0.5 %
3	Trachea, bronchus, lung cancers	0.6 %	1.1 %	0.9 %	1.5 %	2.4 %	1.9 %	2.9 %	4.2 %	3.6 %
3	Other malignant neoplasms	0.5 %	0.6 %	0.6 %	0.5 %	1.4 %	1.0 %	1.4 %	3.1 %	2.2 %

Level	Cause of death	2000			2019			2050		
2	Respiratory diseases	1.9 %	1.8 %	1.9 %	1.3 %	1.5 %	1.4 %	0.6 %	1.1 %	0.8 %
3	Chronic obstructive pulmonary disease	1.8 %	1.7 %	1.8 %	1.3 %	1.4 %	1.4 %	0.7 %	1.1 %	0.9 %
3	Other respiratory diseases	0.0 %	0.0 %	0.0 %	0.0 %	0.0 %	0.0 %	0.0 %	0.0 %	0.0 %
2	Other noncommunicable diseases	0.7 %	0.8 %	0.7 %	0.5 %	0.7 %	0.6 %	1.9 %	0.9 %	1.4 %
1	Injuries	2.8 %	5.6 %	4.2 %	1.7 %	4.1 %	2.9 %	1.5 %	2.0 %	1.7 %
2	Intentional injuries	0.9 %	0.9 %	0.9 %	0.3 %	0.5 %	0.4 %	0.0 %	0.2 %	0.1 %
2	Unintentional injuries	1.9 %	4.7 %	3.3 %	1.4 %	3.6 %	2.5 %	1.3 %	1.8 %	1.6 %
3	Road injury	0.8 %	2.1 %	1.5 %	0.7 %	1.8 %	1.3 %	0.4 %	0.8 %	0.6 %
3	Other unintentional injuries	1.1 %	2.5 %	1.8 %	0.7 %	1.7 %	1.2 %	0.9 %	0.9 %	0.9 %
**Eurasia & Mediterranean**										
1	Communicable, maternal, perinatal and nutritional conditions	8.7 %	9.5 %	9.1 %	4.8 %	5.5 %	5.2 %	2.4 %	3.2 %	2.8 %
2	Infectious and parasitic diseases	6.5 %	8.2 %	7.3 %	3.4 %	4.5 %	3.9 %	1.6 %	2.4 %	2.0 %
2	Maternal and neonatal conditions	2.0 %	1.1 %	1.5 %	1.2 %	0.9 %	1.1 %	0.6 %	0.6 %	0.6 %
2	Nutritional deficiencies	0.2 %	0.2 %	0.2 %	0.1 %	0.1 %	0.1 %	0.2 %	0.1 %	0.1 %
1	Noncommunicable diseases	12.0 %	13.7 %	12.8 %	12.2 %	15.1 %	13.7 %	16.3 %	19.4 %	17.8 %
2	Cardiovascular diseases	6.2 %	6.6 %	6.4 %	6.1 %	7.4 %	6.8 %	8.0 %	9.3 %	8.7 %
3	Ischaemic heart disease	2.9 %	3.7 %	3.3 %	3.0 %	4.0 %	3.5 %	4.2 %	5.3 %	4.8 %
3	Stroke	2.5 %	2.0 %	2.3 %	2.4 %	2.3 %	2.4 %	2.9 %	2.8 %	2.8 %
3	Other cardiovascular diseases	0.8 %	0.8 %	0.8 %	0.7 %	0.9 %	0.8 %	0.9 %	1.1 %	1.0 %
2	Diabetes mellitus	0.5 %	0.4 %	0.5 %	0.9 %	0.7 %	0.8 %	1.5 %	1.1 %	1.3 %
2	Digestive diseases	1.0 %	1.6 %	1.3 %	1.0 %	1.6 %	1.3 %	1.1 %	1.6 %	1.3 %
3	Cirrhosis of the liver	0.8 %	1.1 %	0.9 %	0.7 %	1.2 %	0.9 %	0.7 %	1.1 %	0.9 %
3	Other digestive diseases	0.3 %	0.4 %	0.3 %	0.3 %	0.4 %	0.3 %	0.4 %	0.4 %	0.4 %
2	Malignant neoplasms	2.4 %	2.4 %	2.4 %	2.5 %	3.1 %	2.8 %	3.2 %	4.1 %	3.6 %
3	Breast cancer	0.4 %	0.0 %	0.2 %	0.5 %	0.0 %	0.3 %	0.7 %	0.0 %	0.3 %
3	Cervix uteri cancer	0.4 %	0.0 %	0.2 %	0.3 %	0.0 %	0.2 %	0.3 %	0.0 %	0.1 %
3	Liver cancer	0.1 %	0.3 %	0.2 %	0.2 %	0.5 %	0.3 %	0.2 %	0.4 %	0.3 %
3	Mouth and oropharynx cancers	0.1 %	0.3 %	0.2 %	0.1 %	0.3 %	0.2 %	0.1 %	0.4 %	0.3 %
3	Oesophagus cancer	0.1 %	0.1 %	0.1 %	0.1 %	0.2 %	0.1 %	0.1 %	0.2 %	0.2 %
3	Stomach cancer	0.2 %	0.3 %	0.2 %	0.2 %	0.2 %	0.2 %	0.1 %	0.3 %	0.2 %

Level	Cause of death	2000			2019			2050		
3	Trachea, bronchus, lung cancers	0.2 %	0.7 %	0.4 %	0.3 %	0.8 %	0.5 %	0.3 %	0.9 %	0.6 %
3	Other malignant neoplasms	0.8 %	1.0 %	0.9 %	0.9 %	1.3 %	1.1 %	1.3 %	2.0 %	1.7 %
2	Respiratory diseases	0.6 %	1.0 %	0.8 %	0.6 %	1.0 %	0.8 %	0.7 %	1.3 %	1.0 %
3	Chronic obstructive pulmonary disease	0.3 %	0.7 %	0.5 %	0.3 %	0.7 %	0.5 %	0.5 %	1.0 %	0.7 %
3	Other respiratory diseases	0.3 %	0.3 %	0.3 %	0.2 %	0.3 %	0.3 %	0.2 %	0.3 %	0.3 %
2	Other noncommunicable diseases	1.3 %	1.7 %	1.5 %	1.2 %	1.5 %	1.4 %	1.8 %	2.1 %	1.9 %
1	Injuries	2.2 %	7.1 %	4.7 %	1.3 %	4.8 %	3.0 %	1.1 %	3.4 %	2.2 %
2	Intentional injuries	0.7 %	2.4 %	1.5 %	0.3 %	1.4 %	0.9 %	0.3 %	1.1 %	0.7 %
2	Unintentional injuries	1.5 %	4.8 %	3.1 %	1.0 %	3.3 %	2.2 %	0.7 %	2.3 %	1.5 %
3	Road injury	0.5 %	1.9 %	1.2 %	0.5 %	1.7 %	1.1 %	0.3 %	1.3 %	0.8 %
3	Other unintentional injuries	1.0 %	2.8 %	1.9 %	0.5 %	1.6 %	1.0 %	0.4 %	1.0 %	0.7 %
**High-income**										
1	Communicable, maternal, perinatal and nutritional conditions	1.3 %	2.0 %	1.6 %	1.5 %	1.8 %	1.6 %	1.7 %	2.0 %	1.8 %
2	Infectious and parasitic diseases	0.8 %	1.6 %	1.2 %	0.9 %	1.4 %	1.2 %	1.2 %	1.6 %	1.4 %
2	Maternal and neonatal conditions	0.3 %	0.3 %	0.3 %	0.2 %	0.2 %	0.2 %	0.1 %	0.1 %	0.1 %
2	Nutritional deficiencies	0.1 %	0.0 %	0.1 %	0.1 %	0.1 %	0.1 %	0.1 %	0.1 %	0.1 %
1	Noncommunicable diseases	10.0 %	18.4 %	14.1 %	12.1 %	20.3 %	16.2 %	16.2 %	22.2 %	19.2 %
2	Cardiovascular diseases	2.3 %	6.0 %	4.1 %	2.0 %	5.5 %	3.7 %	2.8 %	5.8 %	4.3 %
3	Ischaemic heart disease	1.6 %	4.1 %	2.8 %	1.0 %	3.4 %	2.2 %	1.3 %	3.2 %	2.2 %
3	Stroke	0.4 %	0.8 %	0.6 %	0.3 %	0.7 %	0.5 %	0.6 %	0.8 %	0.7 %
3	Other cardiovascular diseases	0.6 %	1.0 %	0.8 %	0.9 %	1.3 %	1.1 %	1.5 %	1.9 %	1.7 %
2	Diabetes mellitus	0.4 %	0.4 %	0.4 %	0.3 %	0.5 %	0.4 %	0.3 %	0.7 %	0.5 %
2	Digestive diseases	0.4 %	1.4 %	0.9 %	0.6 %	1.4 %	1.0 %	0.8 %	1.4 %	1.1 %
3	Cirrhosis of the liver	0.2 %	0.9 %	0.5 %	0.3 %	0.8 %	0.5 %	0.3 %	0.7 %	0.5 %
3	Other digestive diseases	0.2 %	0.4 %	0.3 %	0.3 %	0.5 %	0.4 %	0.5 %	0.7 %	0.6 %
2	Malignant neoplasms	5.0 %	7.4 %	6.2 %	5.2 %	7.2 %	6.2 %	5.2 %	6.3 %	5.8 %
3	Breast cancer	1.1 %	0.0 %	0.6 %	0.9 %	0.0 %	0.5 %	0.8 %	0.0 %	0.4 %
3	Cervix uteri cancer	0.1 %	0.0 %	0.1 %	0.1 %	0.0 %	0.1 %	0.1 %	0.0 %	0.0 %
3	Liver cancer	0.1 %	0.6 %	0.3 %	0.1 %	0.5 %	0.3 %	0.1 %	0.5 %	0.3 %
3	Mouth and oropharynx cancers	0.0 %	0.3 %	0.2 %	0.1 %	0.3 %	0.2 %	0.1 %	0.3 %	0.2 %

Level	Cause of death	2000			2019			2050		
3	Oesophagus cancer	0.1 %	0.4 %	0.2 %	0.1 %	0.4 %	0.3 %	0.1 %	0.4 %	0.2 %
3	Stomach cancer	0.2 %	0.6 %	0.4 %	0.1 %	0.5 %	0.3 %	0.1 %	0.2 %	0.1 %
3	Trachea, bronchus, lung cancers	1.4 %	2.8 %	2.1 %	1.8 %	2.6 %	2.2 %	1.4 %	1.7 %	1.5 %
3	Other malignant neoplasms	1.9 %	3.3 %	2.6 %	1.9 %	3.5 %	2.7 %	2.5 %	3.8 %	3.1 %
2	Respiratory diseases	0.7 %	1.2 %	0.9 %	1.2 %	1.7 %	1.5 %	2.2 %	2.5 %	2.3 %
3	Chronic obstructive pulmonary disease	0.4 %	0.8 %	0.6 %	0.8 %	1.1 %	0.9 %	1.4 %	1.5 %	1.4 %
3	Other respiratory diseases	0.2 %	0.4 %	0.3 %	0.4 %	0.6 %	0.5 %	0.7 %	0.9 %	0.8 %
2	Other noncommunicable diseases	1.4 %	2.1 %	1.8 %	3.2 %	4.1 %	3.7 %	5.3 %	5.7 %	5.5 %
1	Injuries	1.1 %	5.7 %	3.4 %	1.3 %	4.5 %	2.9 %	1.4 %	3.6 %	2.5 %
2	Intentional injuries	0.4 %	2.3 %	1.3 %	0.5 %	2.2 %	1.4 %	0.4 %	1.6 %	1.0 %
2	Unintentional injuries	0.6 %	3.3 %	1.9 %	0.7 %	2.2 %	1.4 %	0.9 %	1.9 %	1.4 %
3	Road injury	0.4 %	1.8 %	1.1 %	0.3 %	0.9 %	0.6 %	0.2 %	0.7 %	0.5 %
3	Other unintentional injuries	0.3 %	1.3 %	0.8 %	0.4 %	1.2 %	0.8 %	0.6 %	1.1 %	0.9 %
**India**										
1	Communicable, maternal, perinatal and nutritional conditions	17.7 %	15.1 %	16.4 %	8.1 %	7.5 %	7.8 %	3.2 %	3.4 %	3.3 %
2	Infectious and parasitic diseases	14.0 %	13.2 %	13.6 %	6.5 %	6.3 %	6.4 %	2.6 %	2.8 %	2.7 %
2	Maternal and neonatal conditions	3.1 %	1.5 %	2.3 %	1.4 %	1.1 %	1.2 %	0.5 %	0.6 %	0.6 %
2	Nutritional deficiencies	0.5 %	0.3 %	0.4 %	0.1 %	0.0 %	0.1 %	0.0 %	0.0 %	0.0 %
1	Noncommunicable diseases	7.3 %	9.1 %	8.2 %	10.5 %	12.7 %	11.6 %	17.1 %	20.7 %	18.9 %
2	Cardiovascular diseases	2.7 %	3.8 %	3.3 %	4.5 %	5.9 %	5.2 %	7.6 %	8.7 %	8.2 %
3	Ischaemic heart disease	1.2 %	2.3 %	1.8 %	2.4 %	4.0 %	3.2 %	4.4 %	6.2 %	5.3 %
3	Stroke	0.8 %	0.9 %	0.9 %	1.3 %	1.3 %	1.3 %	2.2 %	1.8 %	2.0 %
3	Other cardiovascular diseases	0.7 %	0.5 %	0.6 %	0.8 %	0.5 %	0.6 %	1.0 %	0.6 %	0.8 %
2	Diabetes mellitus	0.3 %	0.3 %	0.3 %	0.6 %	0.5 %	0.6 %	1.1 %	1.3 %	1.2 %
2	Digestive diseases	1.0 %	1.7 %	1.4 %	0.9 %	1.8 %	1.4 %	0.7 %	1.6 %	1.2 %
3	Cirrhosis of the liver	0.5 %	1.0 %	0.8 %	0.5 %	1.3 %	0.9 %	0.5 %	1.2 %	0.8 %
3	Other digestive diseases	0.6 %	0.7 %	0.6 %	0.4 %	0.5 %	0.4 %	0.3 %	0.4 %	0.3 %
2	Malignant neoplasms	0.8 %	0.6 %	0.7 %	1.6 %	1.3 %	1.4 %	2.9 %	3.8 %	3.4 %
3	Breast cancer	0.1 %	0.0 %	0.0 %	0.3 %	0.0 %	0.2 %	0.6 %	0.0 %	0.3 %
3	Cervix uteri cancer	0.2 %	0.0 %	0.1 %	0.3 %	0.0 %	0.2 %	0.4 %	0.0 %	0.2 %

Level	Cause of death		2000			2019			2050	
3	Liver cancer	0.0 %	0.0 %	0.0 %	0.0 %	0.1 %	0.0 %	0.0 %	0.1 %	0.1 %
3	Mouth and oropharynx cancers	0.2 %	0.3 %	0.2 %	0.3 %	0.5 %	0.4 %	0.4 %	1.1 %	0.8 %
3	Oesophagus cancer	0.1 %	0.1 %	0.1 %	0.1 %	0.1 %	0.1 %	0.1 %	0.2 %	0.2 %
3	Stomach cancer	0.1 %	0.1 %	0.1 %	0.2 %	0.2 %	0.2 %	0.3 %	0.2 %	0.2 %
3	Trachea, bronchus, lung cancers	0.0 %	0.1 %	0.1 %	0.1 %	0.3 %	0.2 %	0.2 %	0.6 %	0.4 %
3	Other malignant neoplasms	0.2 %	0.2 %	0.2 %	0.3 %	0.5 %	0.4 %	0.9 %	1.8 %	1.4 %
2	Respiratory diseases	1.5 %	1.6 %	1.6 %	2.0 %	2.1 %	2.0 %	3.3 %	3.1 %	3.2 %
3	Chronic obstructive pulmonary disease	0.9 %	1.1 %	1.0 %	1.3 %	1.5 %	1.4 %	2.6 %	2.6 %	2.6 %
3	Other respiratory diseases	0.6 %	0.5 %	0.5 %	0.7 %	0.5 %	0.6 %	0.7 %	0.5 %	0.6 %
2	Other noncommunicable diseases	1.0 %	1.1 %	1.1 %	1.0 %	1.2 %	1.1 %	1.6 %	2.2 %	1.9 %
1	Injuries	3.3 %	5.0 %	4.2 %	2.6 %	4.5 %	3.6 %	2.6 %	3.9 %	3.3 %
2	Intentional injuries	1.2 %	1.4 %	1.3 %	0.8 %	1.3 %	1.1 %	0.6 %	1.1 %	0.9 %
2	Unintentional injuries	2.0 %	3.6 %	2.8 %	1.7 %	3.2 %	2.5 %	2.0 %	2.8 %	2.4 %
3	Road injury	0.3 %	1.3 %	0.8 %	0.3 %	1.5 %	0.9 %	0.4 %	1.3 %	0.8 %
3	Other unintentional injuries	1.8 %	2.2 %	2.0 %	1.4 %	1.7 %	1.6 %	1.6 %	1.5 %	1.5 %
**Latin America & Caribbean**										
1	Communicable, maternal, perinatal and nutritional conditions	5.6 %	6.4 %	6.0 %	3.4 %	3.9 %	3.6 %	3.1 %	3.3 %	3.2 %
2	Infectious and parasitic diseases	3.6 %	4.8 %	4.2 %	2.3 %	2.9 %	2.6 %	2.4 %	2.8 %	2.6 %
2	Maternal and neonatal conditions	1.5 %	1.1 %	1.3 %	0.8 %	0.7 %	0.8 %	0.3 %	0.3 %	0.3 %
2	Nutritional deficiencies	0.5 %	0.5 %	0.5 %	0.2 %	0.2 %	0.2 %	0.2 %	0.2 %	0.2 %
1	Noncommunicable diseases	10.2 %	12.0 %	11.1 %	11.5 %	14.2 %	12.8 %	15.3 %	18.5 %	16.9 %
2	Cardiovascular diseases	2.9 %	3.7 %	3.3 %	3.1 %	4.6 %	3.9 %	4.1 %	6.1 %	5.1 %
3	Ischaemic heart disease	1.0 %	1.8 %	1.4 %	1.4 %	2.5 %	1.9 %	2.3 %	3.6 %	2.9 %
3	Stroke	1.2 %	1.1 %	1.2 %	1.0 %	1.1 %	1.1 %	0.9 %	1.2 %	1.0 %
3	Other cardiovascular diseases	0.7 %	0.7 %	0.7 %	0.8 %	0.9 %	0.8 %	1.0 %	1.3 %	1.1 %
2	Diabetes mellitus	1.1 %	0.7 %	0.9 %	1.4 %	1.2 %	1.3 %	1.9 %	1.9 %	1.9 %
2	Digestive diseases	0.9 %	2.1 %	1.5 %	1.0 %	2.2 %	1.6 %	1.4 %	2.5 %	1.9 %
3	Cirrhosis of the liver	0.3 %	1.2 %	0.8 %	0.3 %	1.2 %	0.7 %	0.3 %	1.2 %	0.8 %
3	Other digestive diseases	0.6 %	0.8 %	0.7 %	0.8 %	1.0 %	0.9 %	1.1 %	1.2 %	1.2 %
2	Malignant neoplasms	2.6 %	2.1 %	2.3 %	2.9 %	2.5 %	2.7 %	4.0 %	3.7 %	3.8 %

Level	Cause of death	2000			2019			2050		
3	Breast cancer	0.4 %	0.0 %	0.2 %	0.5 %	0.0 %	0.3 %	0.8 %	0.0 %	0.4 %
3	Cervix uteri cancer	0.6 %	0.0 %	0.3 %	0.5 %	0.0 %	0.3 %	0.4 %	0.0 %	0.2 %
3	Liver cancer	0.1 %	0.1 %	0.1 %	0.1 %	0.2 %	0.2 %	0.2 %	0.3 %	0.2 %
3	Mouth and oropharynx cancers	0.0 %	0.1 %	0.1 %	0.0 %	0.2 %	0.1 %	0.0 %	0.2 %	0.1 %
3	Oesophagus cancer	0.0 %	0.2 %	0.1 %	0.0 %	0.2 %	0.1 %	0.0 %	0.2 %	0.1 %
3	Stomach cancer	0.2 %	0.3 %	0.3 %	0.3 %	0.4 %	0.3 %	0.2 %	0.3 %	0.3 %
3	Trachea, bronchus, lung cancers	0.2 %	0.4 %	0.3 %	0.4 %	0.4 %	0.4 %	0.5 %	0.5 %	0.5 %
3	Other malignant neoplasms	0.9 %	1.3 %	1.1 %	1.0 %	1.6 %	1.3 %	1.8 %	2.5 %	2.2 %
2	Respiratory diseases	0.9 %	1.1 %	1.0 %	0.8 %	0.9 %	0.9 %	1.1 %	1.0 %	1.0 %
3	Chronic obstructive pulmonary disease	0.4 %	0.5 %	0.5 %	0.5 %	0.5 %	0.5 %	0.7 %	0.6 %	0.7 %
3	Other respiratory diseases	0.5 %	0.5 %	0.5 %	0.4 %	0.4 %	0.4 %	0.3 %	0.4 %	0.4 %
2	Other noncommunicable diseases	1.8 %	2.3 %	2.0 %	2.3 %	2.8 %	2.5 %	3.0 %	3.4 %	3.2 %
1	Injuries	2.2 %	11.4 %	6.7 %	1.6 %	8.8 %	5.1 %	1.0 %	4.6 %	2.8 %
2	Intentional injuries	0.8 %	6.1 %	3.4 %	0.7 %	5.1 %	2.9 %	0.4 %	2.8 %	1.6 %
2	Unintentional injuries	1.3 %	5.3 %	3.3 %	0.8 %	3.7 %	2.3 %	0.6 %	1.8 %	1.2 %
3	Road injury	0.6 %	2.6 %	1.6 %	0.4 %	2.1 %	1.3 %	0.2 %	1.0 %	0.6 %
3	Other unintentional injuries	0.7 %	2.7 %	1.7 %	0.4 %	1.5 %	1.0 %	0.4 %	0.8 %	0.6 %
**Sub-Saharan Africa**										
1	Communicable, maternal, perinatal and nutritional conditions	35.1 %	31.8 %	33.5 %	20.4 %	19.6 %	20.0 %	8.7 %	10.2 %	9.5 %
2	Infectious and parasitic diseases	28.8 %	29.3 %	29.0 %	15.8 %	17.4 %	16.6 %	5.9 %	8.5 %	7.2 %
2	Maternal and neonatal conditions	5.5 %	1.6 %	3.6 %	4.2 %	1.8 %	3.0 %	2.4 %	1.2 %	1.8 %
2	Nutritional deficiencies	0.8 %	0.9 %	0.9 %	0.4 %	0.4 %	0.4 %	0.4 %	0.4 %	0.4 %
1	Noncommunicable diseases	7.2 %	7.7 %	7.5 %	8.5 %	8.5 %	8.5 %	11.7 %	11.1 %	11.4 %
2	Cardiovascular diseases	2.4 %	2.4 %	2.4 %	2.5 %	2.5 %	2.5 %	3.0 %	3.1 %	3.0 %
3	Ischaemic heart disease	0.6 %	0.8 %	0.7 %	0.7 %	0.9 %	0.8 %	1.1 %	1.3 %	1.2 %
3	Stroke	1.1 %	1.0 %	1.0 %	1.0 %	1.0 %	1.0 %	1.2 %	1.1 %	1.1 %
3	Other cardiovascular diseases	0.8 %	0.6 %	0.7 %	0.7 %	0.6 %	0.6 %	0.8 %	0.7 %	0.7 %
2	Diabetes mellitus	0.4 %	0.4 %	0.4 %	0.5 %	0.5 %	0.5 %	0.7 %	0.7 %	0.7 %
2	Digestive diseases	1.0 %	1.8 %	1.4 %	1.0 %	1.8 %	1.4 %	1.0 %	1.8 %	1.4 %
3	Cirrhosis of the liver	0.6 %	1.1 %	0.8 %	0.6 %	1.1 %	0.8 %	0.5 %	1.0 %	0.8 %

Level	Cause of death	2000			2019			2050		
3	Other digestive diseases	0.4 %	0.7 %	0.5 %	0.4 %	0.7 %	0.6 %	0.5 %	0.8 %	0.7 %
2	Malignant neoplasms	1.3 %	0.7 %	1.0 %	2.2 %	1.1 %	1.7 %	4.4 %	2.2 %	3.3 %
3	Breast cancer	0.2 %	0.0 %	0.1 %	0.5 %	0.0 %	0.3 %	1.1 %	0.0 %	0.5 %
3	Cervix uteri cancer	0.5 %	0.0 %	0.3 %	0.6 %	0.0 %	0.3 %	1.0 %	0.0 %	0.5 %
3	Liver cancer	0.1 %	0.1 %	0.1 %	0.1 %	0.2 %	0.1 %	0.2 %	0.3 %	0.2 %
3	Mouth and oropharynx cancers	0.0 %	0.1 %	0.0 %	0.1 %	0.1 %	0.1 %	0.1 %	0.1 %	0.1 %
3	Oesophagus cancer	0.1 %	0.1 %	0.1 %	0.1 %	0.1 %	0.1 %	0.1 %	0.2 %	0.1 %
3	Stomach cancer	0.0 %	0.0 %	0.0 %	0.1 %	0.1 %	0.1 %	0.1 %	0.1 %	0.1 %
3	Trachea, bronchus, lung cancers	0.0 %	0.0 %	0.0 %	0.0 %	0.1 %	0.0 %	0.1 %	0.1 %	0.1 %
3	Other malignant neoplasms	0.4 %	0.5 %	0.4 %	0.8 %	0.8 %	0.8 %	1.7 %	1.5 %	1.6 %
2	Respiratory diseases	0.6 %	0.6 %	0.6 %	0.5 %	0.5 %	0.5 %	0.6 %	0.6 %	0.6 %
3	Chronic obstructive pulmonary disease	0.2 %	0.2 %	0.2 %	0.2 %	0.2 %	0.2 %	0.2 %	0.3 %	0.3 %
3	Other respiratory diseases	0.4 %	0.3 %	0.4 %	0.3 %	0.3 %	0.3 %	0.4 %	0.4 %	0.4 %
2	Other noncommunicable diseases	1.6 %	1.9 %	1.7 %	1.8 %	2.2 %	2.0 %	2.1 %	2.6 %	2.4 %
1	Injuries	3.6 %	8.5 %	6.0 %	3.2 %	7.4 %	5.3 %	3.5 %	7.9 %	5.7 %
2	Intentional injuries	1.3 %	3.6 %	2.4 %	0.7 %	2.3 %	1.5 %	1.0 %	2.5 %	1.7 %
2	Unintentional injuries	2.3 %	4.8 %	3.6 %	2.5 %	5.1 %	3.8 %	2.5 %	5.4 %	4.0 %
3	Road injury	1.1 %	2.6 %	1.8 %	1.2 %	3.0 %	2.1 %	1.3 %	3.6 %	2.5 %
3	Other unintentional injuries	1.2 %	2.2 %	1.7 %	1.2 %	2.0 %	1.6 %	1.2 %	1.8 %	1.5 %
**World**										
1	Communicable, maternal, perinatal and nutritional conditions	10.3 %	9.9 %	10.1 %	6.3 %	6.4 %	6.3 %	3.6 %	4.2 %	3.9 %
2	Infectious and parasitic diseases	8.0 %	8.7 %	8.4 %	4.8 %	5.4 %	5.1 %	2.5 %	3.5 %	3.0 %
2	Maternal and neonatal conditions	2.0 %	1.0 %	1.5 %	1.3 %	0.8 %	1.1 %	0.8 %	0.6 %	0.7 %
2	Nutritional deficiencies	0.3 %	0.3 %	0.3 %	0.2 %	0.1 %	0.1 %	0.2 %	0.2 %	0.2 %
1	Noncommunicable diseases	9.5 %	12.5 %	11.0 %	11.2 %	15.0 %	13.1 %	16.0 %	19.0 %	17.5 %
2	Cardiovascular diseases	3.6 %	4.8 %	4.2 %	4.4 %	6.0 %	5.2 %	6.4 %	7.7 %	7.0 %
3	Ischaemic heart disease	1.4 %	2.4 %	1.9 %	1.9 %	3.2 %	2.6 %	3.3 %	4.3 %	3.8 %
3	Stroke	1.6 %	1.6 %	1.6 %	1.8 %	2.0 %	1.9 %	2.1 %	2.2 %	2.1 %
3	Other cardiovascular diseases	0.7 %	0.7 %	0.7 %	0.7 %	0.8 %	0.7 %	1.1 %	1.1 %	1.1 %
2	Diabetes mellitus	0.4 %	0.3 %	0.4 %	0.6 %	0.5 %	0.6 %	1.0 %	1.0 %	1.0 %

Level	Cause of death	2000			2019			2050		
2	Digestive diseases	0.7 %	1.5 %	1.1 %	0.7 %	1.5 %	1.1 %	0.8 %	1.5 %	1.2 %
3	Cirrhosis of the liver	0.4 %	1.0 %	0.7 %	0.4 %	1.0 %	0.7 %	0.5 %	0.9 %	0.7 %
3	Other digestive diseases	0.3 %	0.5 %	0.4 %	0.3 %	0.5 %	0.4 %	0.4 %	0.5 %	0.5 %
2	Malignant neoplasms	2.5 %	3.1 %	2.8 %	3.0 %	3.7 %	3.4 %	4.2 %	4.7 %	4.4 %
3	Breast cancer	0.4 %	0.0 %	0.2 %	0.5 %	0.0 %	0.2 %	0.8 %	0.0 %	0.4 %
3	Cervix uteri cancer	0.3 %	0.0 %	0.1 %	0.4 %	0.0 %	0.2 %	0.5 %	0.0 %	0.2 %
3	Liver cancer	0.2 %	0.5 %	0.3 %	0.1 %	0.4 %	0.3 %	0.2 %	0.4 %	0.3 %
3	Mouth and oropharynx cancers	0.1 %	0.2 %	0.2 %	0.1 %	0.3 %	0.2 %	0.2 %	0.4 %	0.3 %
3	Oesophagus cancer	0.1 %	0.3 %	0.2 %	0.1 %	0.3 %	0.2 %	0.1 %	0.3 %	0.2 %
3	Stomach cancer	0.3 %	0.4 %	0.4 %	0.3 %	0.5 %	0.4 %	0.2 %	0.3 %	0.2 %
3	Trachea, bronchus, lung cancers	0.5 %	0.9 %	0.7 %	0.7 %	1.1 %	0.9 %	0.8 %	1.2 %	1.0 %
3	Other malignant neoplasms	0.8 %	1.1 %	1.0 %	0.9 %	1.5 %	1.2 %	1.5 %	2.3 %	1.9 %
2	Respiratory diseases	1.1 %	1.3 %	1.2 %	1.1 %	1.3 %	1.2 %	1.3 %	1.6 %	1.5 %
3	Chronic obstructive pulmonary disease	0.8 %	0.9 %	0.8 %	0.8 %	1.0 %	0.9 %	1.0 %	1.2 %	1.1 %
3	Other respiratory diseases	0.3 %	0.3 %	0.3 %	0.3 %	0.3 %	0.3 %	0.4 %	0.4 %	0.4 %
2	Other noncommunicable diseases	1.2 %	1.5 %	1.4 %	1.5 %	1.9 %	1.7 %	2.4 %	2.6 %	2.5 %
1	Injuries	2.5 %	6.6 %	4.6 %	1.9 %	5.2 %	3.6 %	2.0 %	4.4 %	3.2 %
2	Intentional injuries	0.9 %	2.3 %	1.6 %	0.5 %	1.7 %	1.1 %	0.5 %	1.5 %	1.0 %
2	Unintentional injuries	1.6 %	4.3 %	3.0 %	1.3 %	3.5 %	2.4 %	1.5 %	2.9 %	2.2 %
3	Road injury	0.6 %	2.0 %	1.3 %	0.6 %	1.8 %	1.2 %	0.5 %	1.6 %	1.1 %
3	Other unintentional injuries	1.0 %	2.3 %	1.7 %	0.8 %	1.6 %	1.2 %	0.9 %	1.3 %	1.1 %

Figure 3. Economic values of reducing avoidable mortality: Level 1 causes of death

Economic values of reducing avoidable mortality, measured as percent of annual income, assigned to selected causes of death (level 1 causes of death) for the six regions and the world, in the years 2000, 2019, and 2050; females and males.

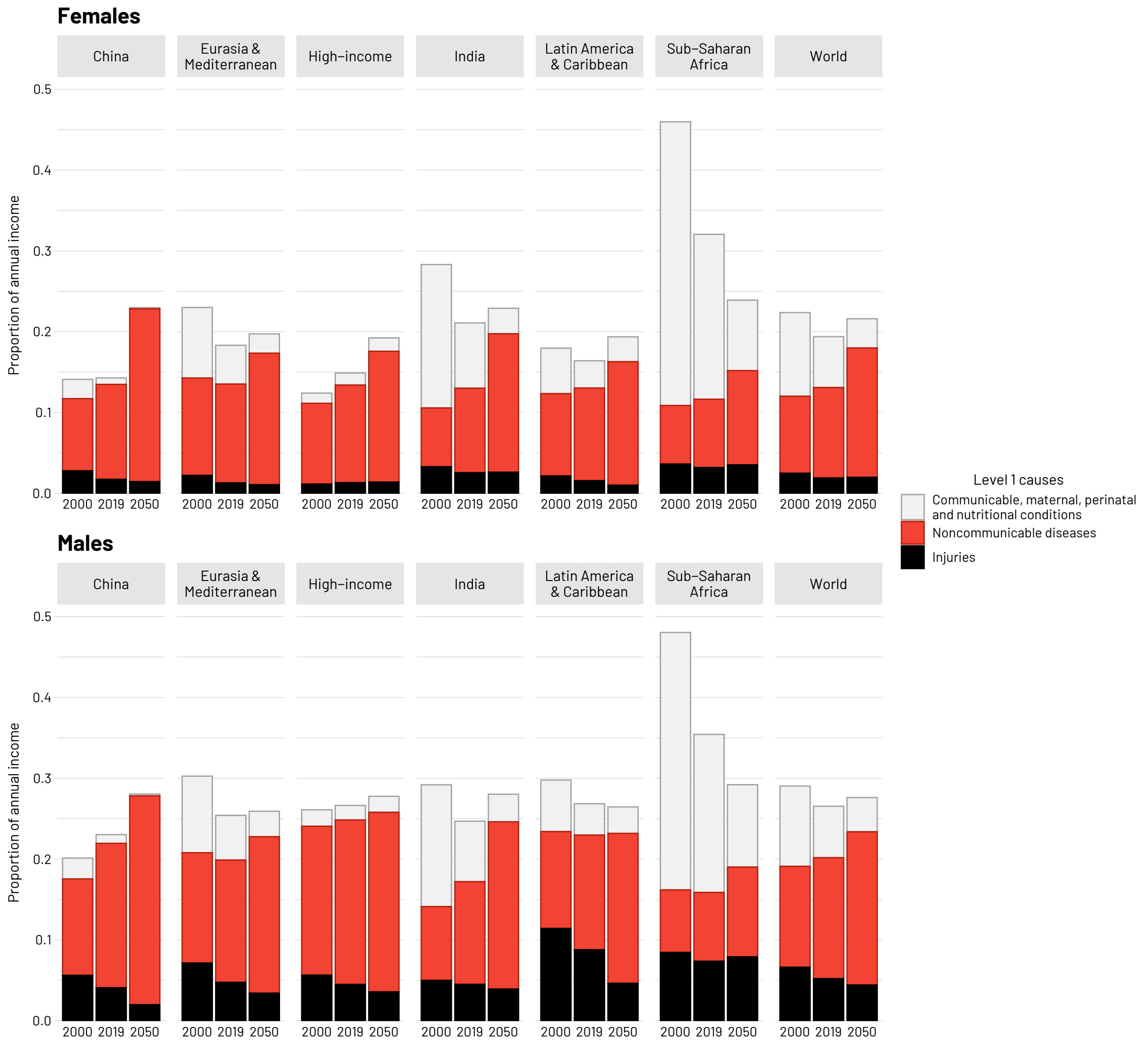

Figure 4. Economic values of reducing avoidable mortality: Level 3 causes of death

Economic values of reducing avoidable mortality, measured as percent of annual income, assigned to selected causes of death (level 3 causes of death) for the six regions and the world, in the years 2000, 2019, and 2050; females and males.

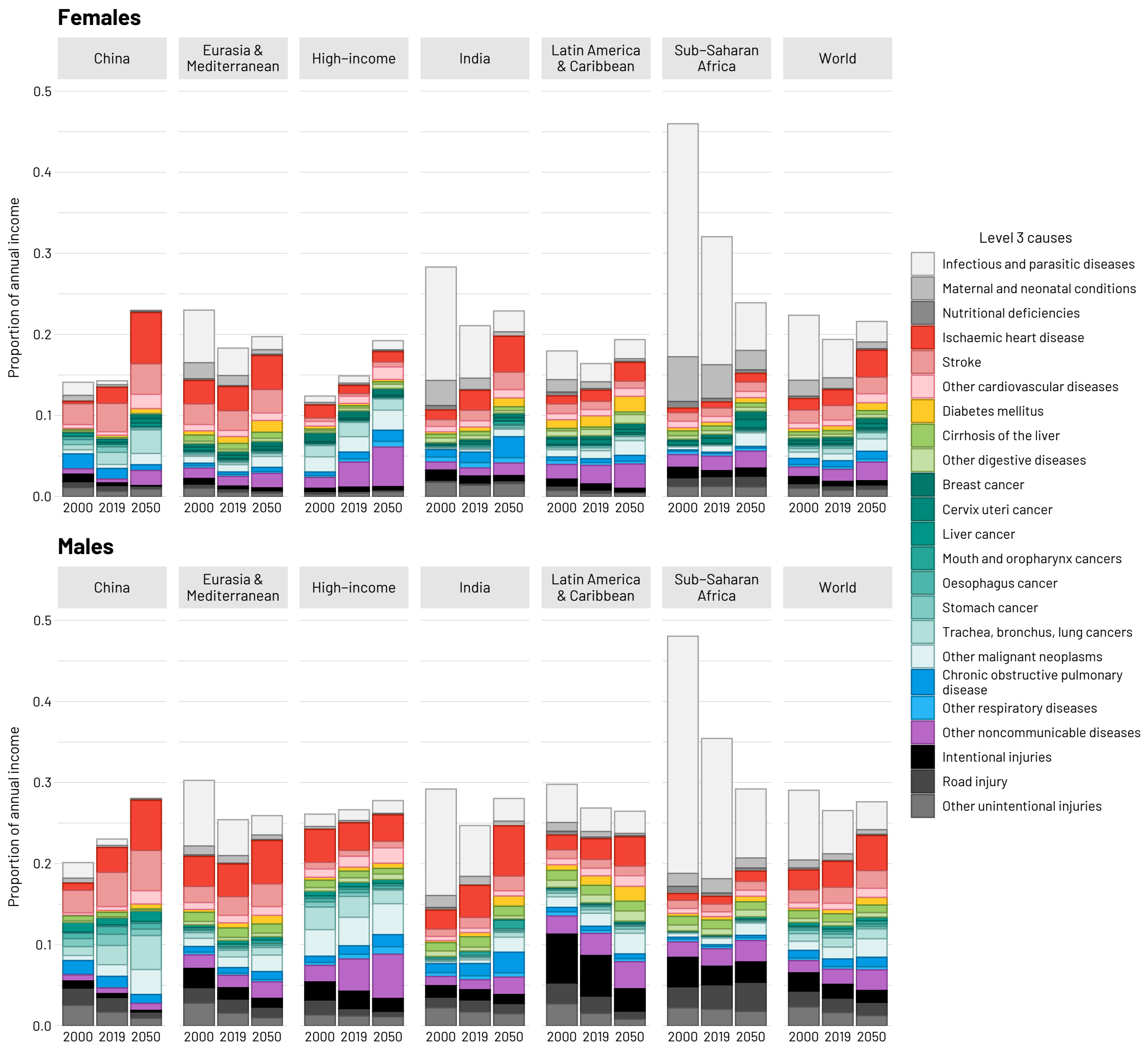

Figure 5. Distribution of economic values of reducing avoidable mortality: Level 1 causes of death

Distribution by cause of death (level 1 causes) of the monetary value associated with reducing avoidable mortality for the six regions and the world, in the years 2000, 2019, and 2050. These fractions (all summing up to 1) are scalars that when multiplied to an economic value of reducing avoidable mortality yield an economic value associated with reducing avoidable mortality by cause for a given calendar year and region.

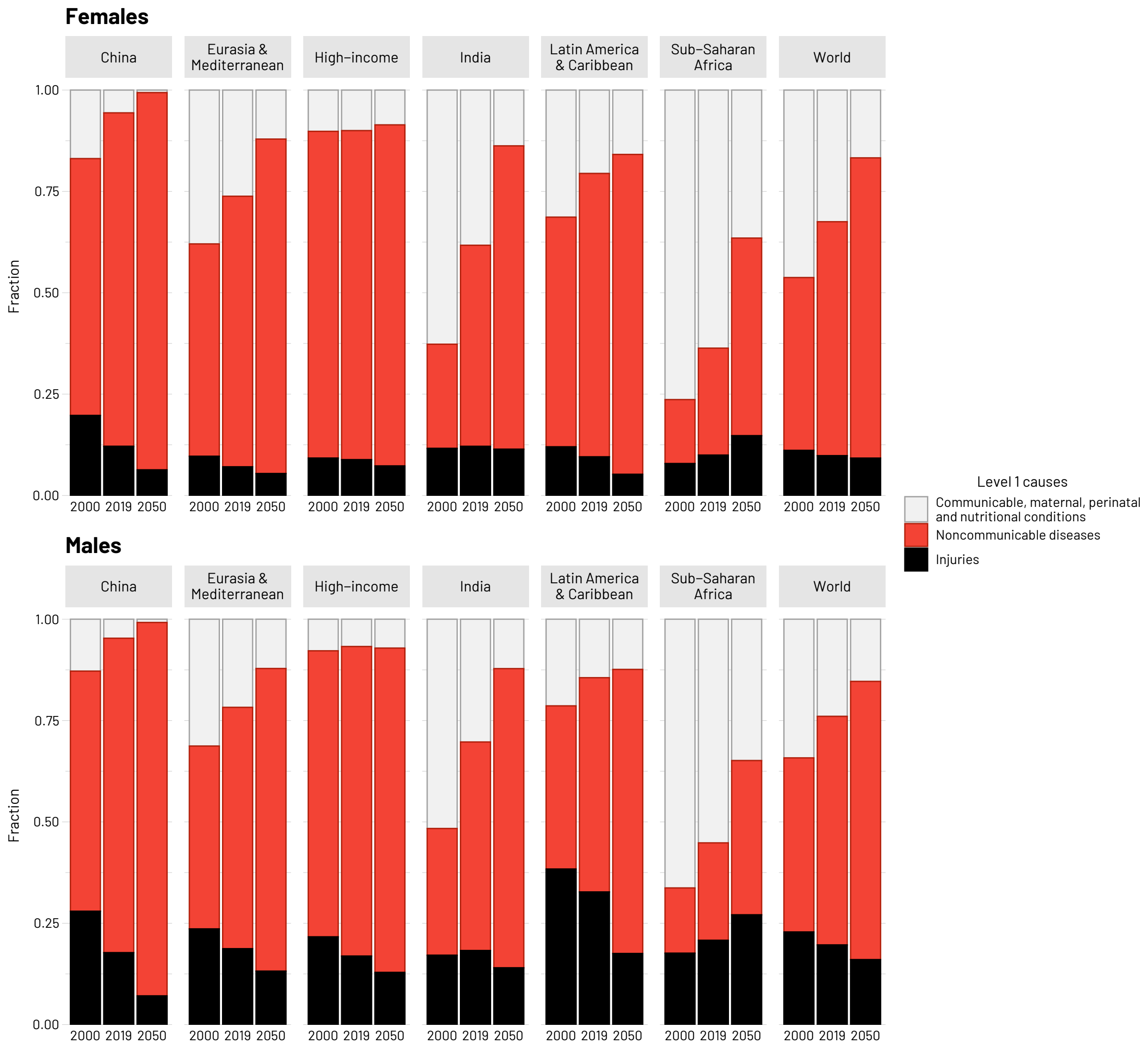

Figure 6. Distribution of economic values of reducing avoidable mortality: Level 2 causes of death

Distribution by cause of death (level 2 causes) of the monetary value associated with reducing avoidable mortality for the six regions and the world, in the years 2000, 2019, and 2050. These fractions (all summing up to 1) are scalars that when multiplied to an economic value of reducing avoidable mortality yield an economic value associated with reducing avoidable mortality by cause for a given calendar year and region.

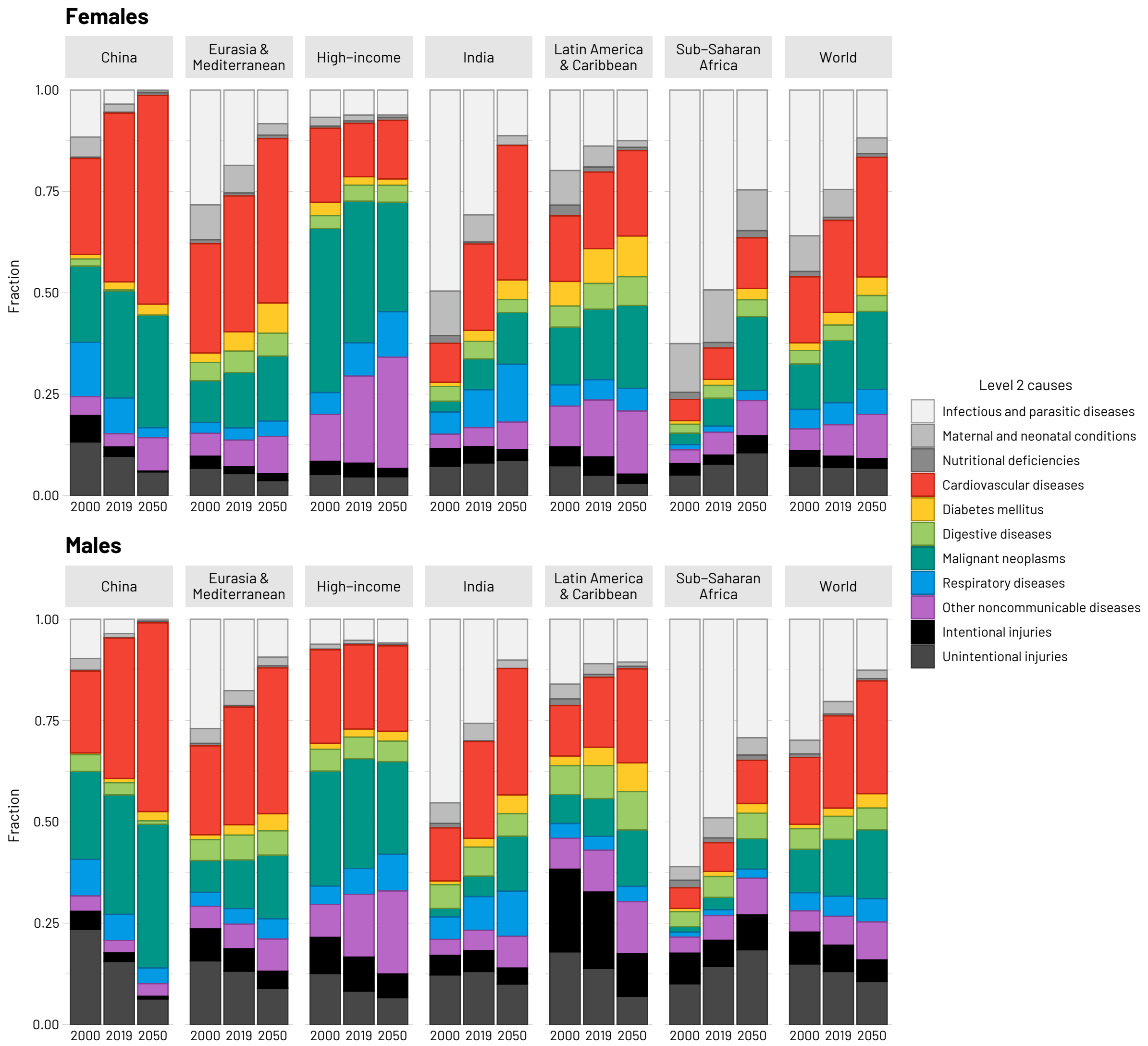

Figure 7. Distribution of economic values of reducing avoidable mortality: Level 3 causes of death

Distribution by cause of death (level 3 causes) of the monetary value associated with reducing avoidable mortality for the six regions and the world, in the years 2000, 2019, and 2050. These fractions (all summing up to 1) are scalars that when multiplied to an economic value of reducing avoidable mortality yield an economic value associated with reducing avoidable mortality by cause for a given calendar year and region.

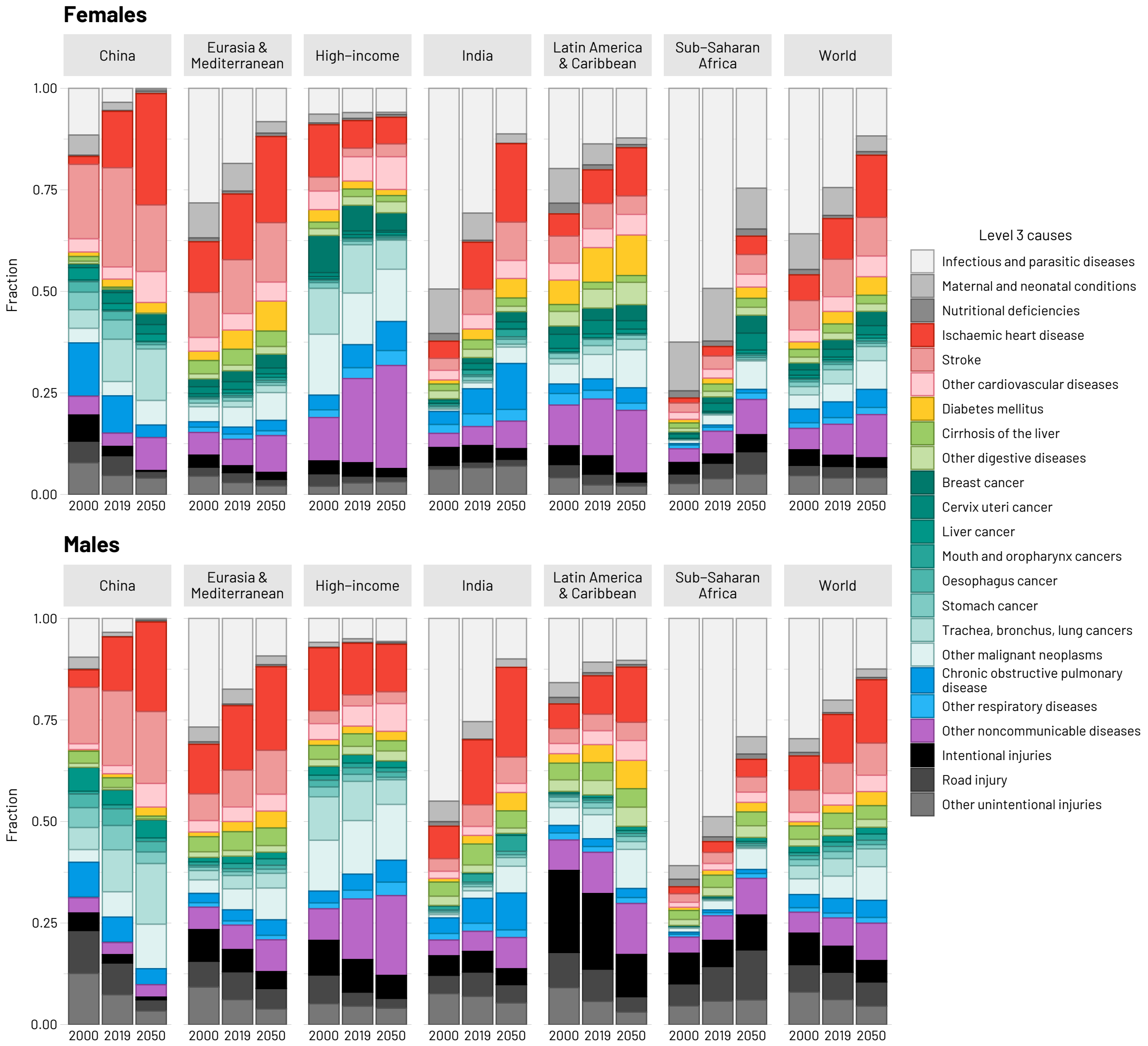

2. Additional findings

Figure 8 displays the rankings of causes of death (level 2 causes) by using either economic value or mortality level as ranking criterion, for the six analytical regions, females and males, for the years 2019 and 2050.

Figure 8. Rankings of causes of death by economic value and mortality level

Rankings of causes of death (level 2 causes), by either economic value of reducing avoidable mortality (left) or mortality levels (right, total number of deaths), for the six regions and the world, females and males, in the years 2019 and 2050.

(A) China

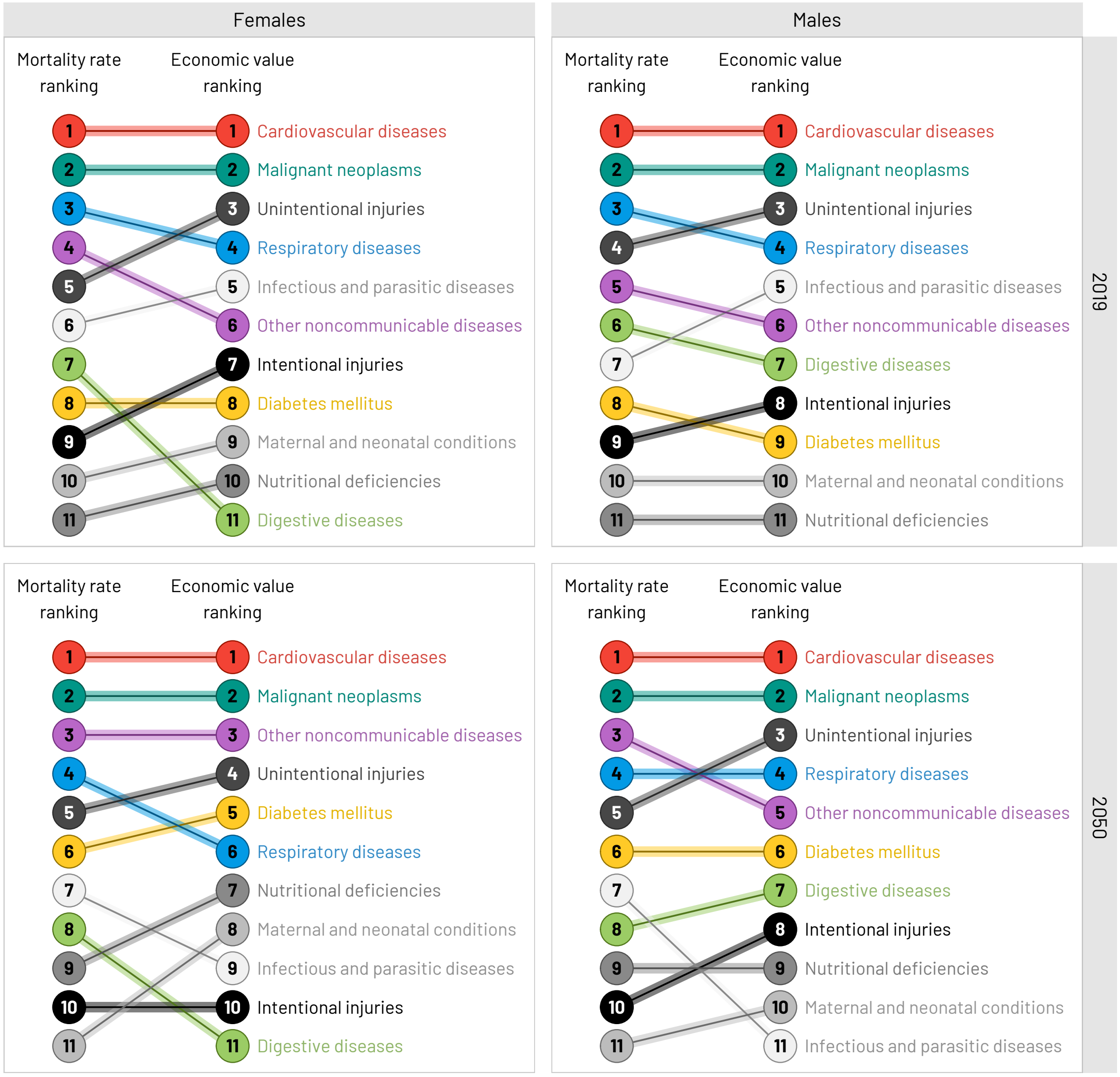

(B) Eurasia & Mediterranean

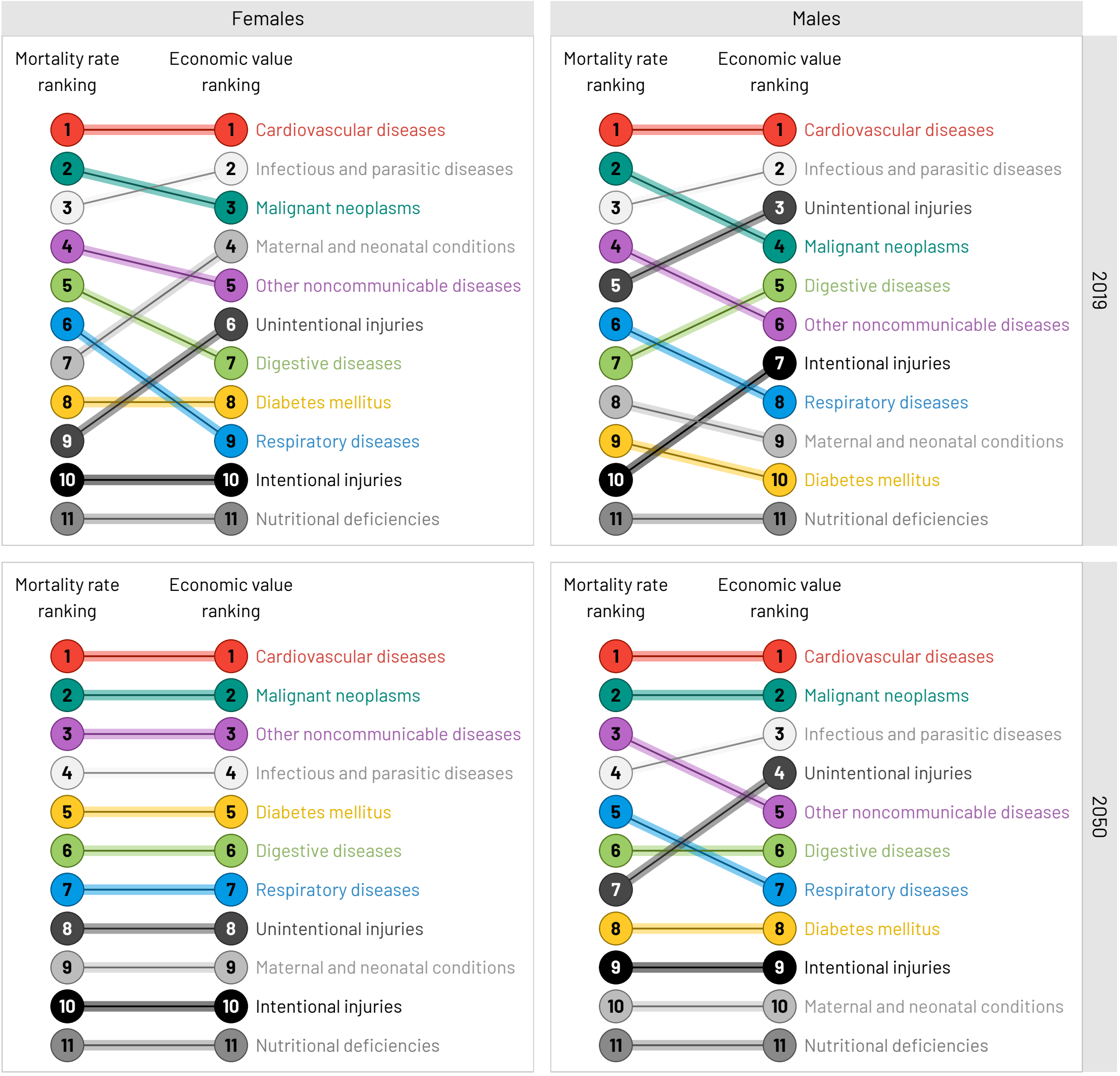

(C) High-income

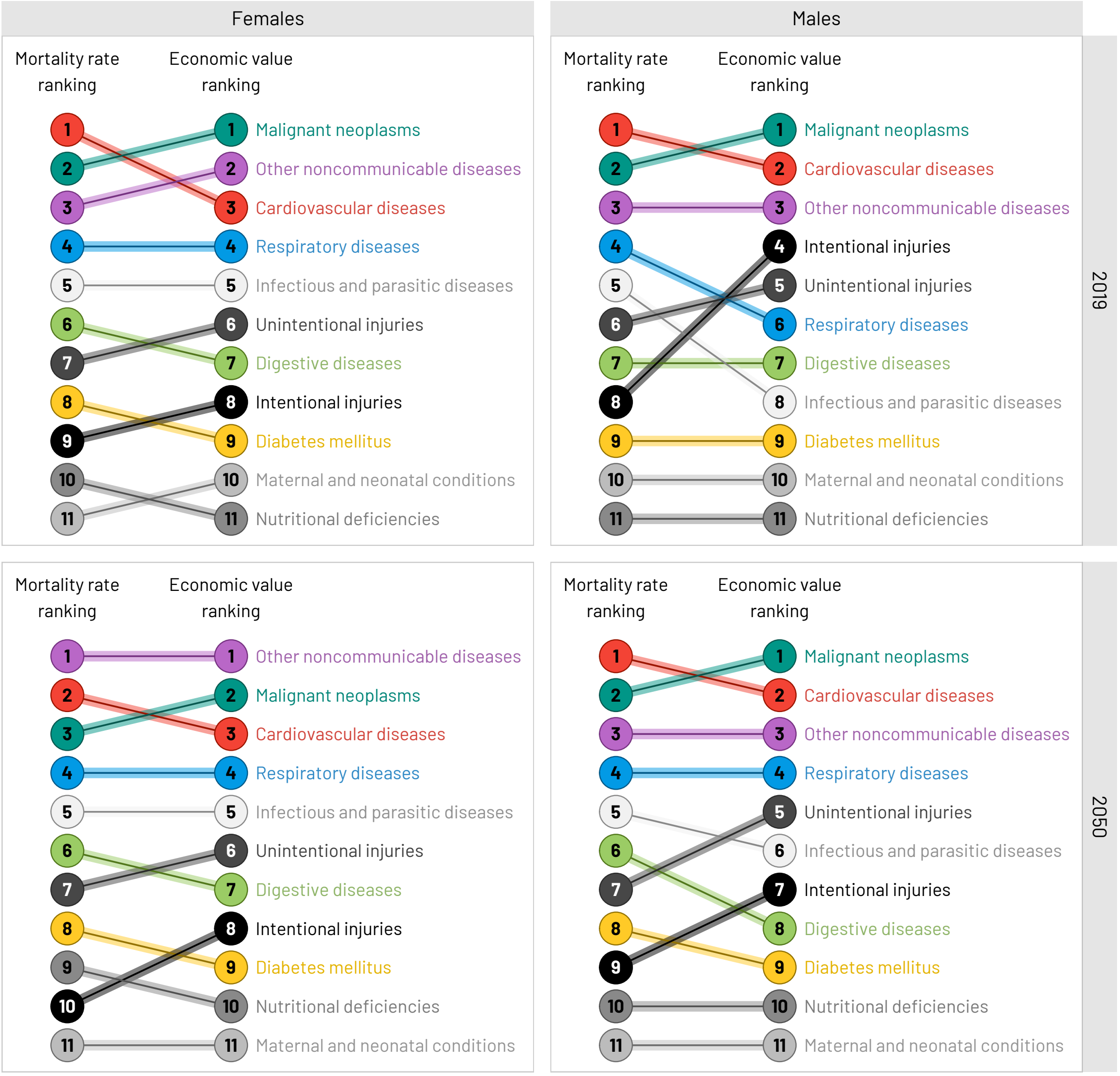

(D) India

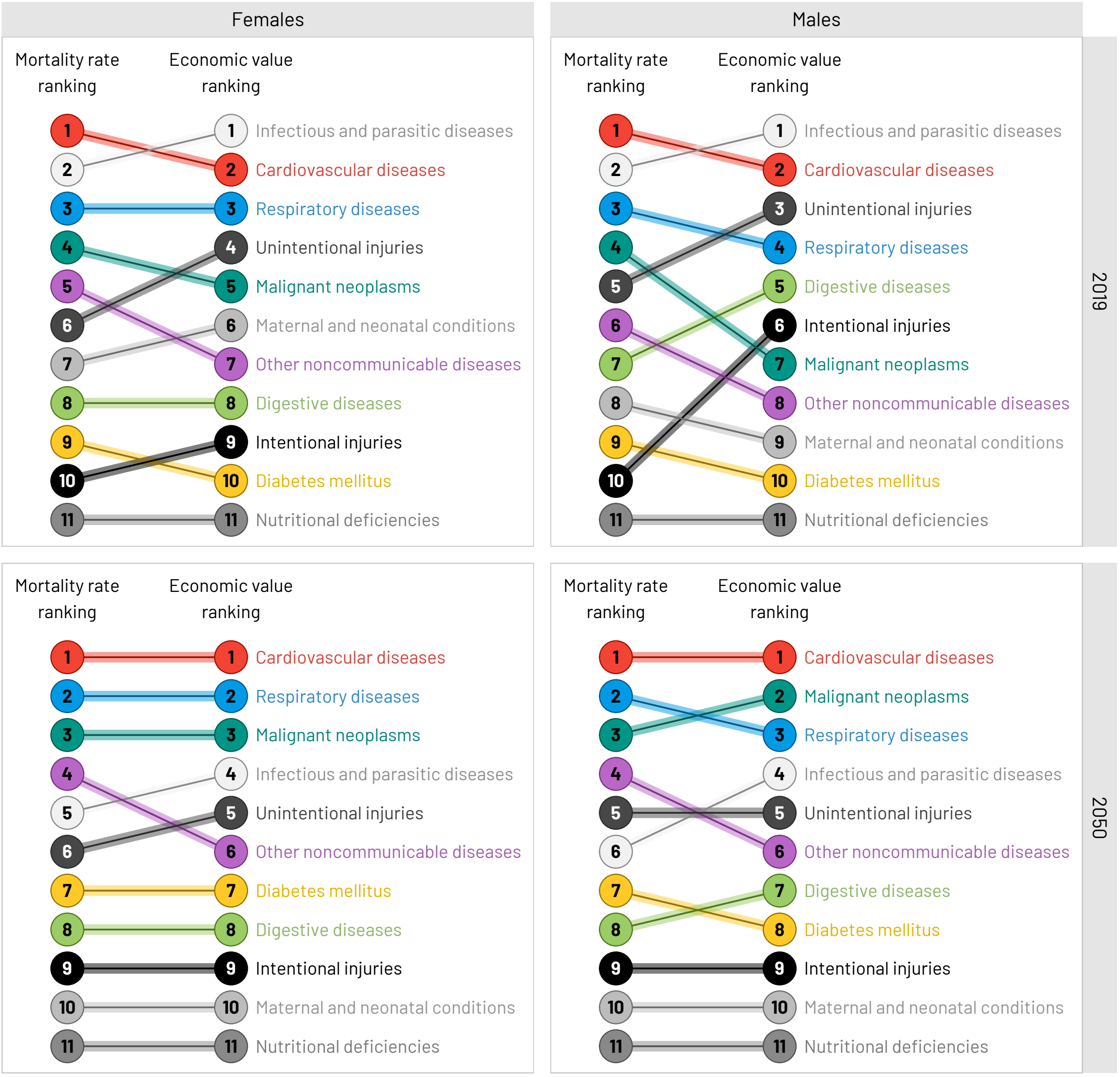

Females
Males
Mortality rate ranking
Economic value ranking
1 Infectious and parasitic diseases
2 Cardiovascular diseases
3 Respiratory diseases
4 Unintentional injuries
5 Malignant neoplasms
6 Maternal and neonatal conditions
7 Other noncommunicable diseases
8 Digestive diseases
9 Intentional injuries
10 Diabetes mellitus
11 Nutritional deficiencies
1 Infectious and parasitic diseases
2 Cardiovascular diseases
3 Unintentional injuries
4 Respiratory diseases
5 Digestive diseases
6 Intentional injuries
7 Malignant neoplasms
8 Other noncommunicable diseases
9 Maternal and neonatal conditions
10 Diabetes mellitus
11 Nutritional deficiencies
2019
1 Cardiovascular diseases
2 Respiratory diseases
3 Malignant neoplasms
4 Infectious and parasitic diseases
5 Unintentional injuries
6 Other noncommunicable diseases
7 Diabetes mellitus
8 Digestive diseases
9 Intentional injuries
10 Maternal and neonatal conditions
11 Nutritional deficiencies
1 Cardiovascular diseases
2 Malignant neoplasms
3 Respiratory diseases
4 Infectious and parasitic diseases
5 Unintentional injuries
6 Other noncommunicable diseases
7 Digestive diseases
8 Diabetes mellitus
9 Intentional injuries
10 Maternal and neonatal conditions
11 Nutritional deficiencies
2050

(E) Latin America & Caribbean

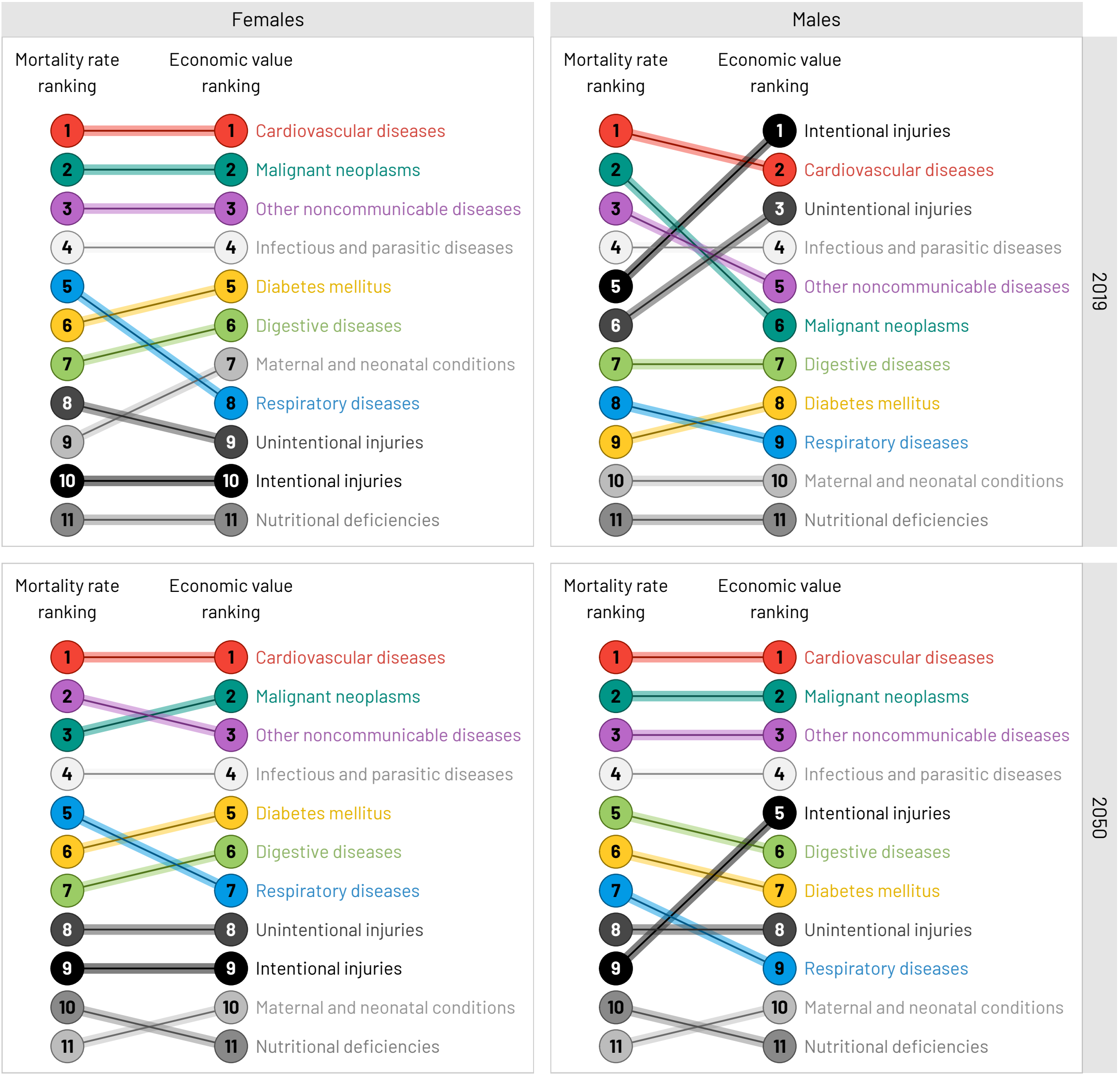

(F) Sub-Saharan Africa

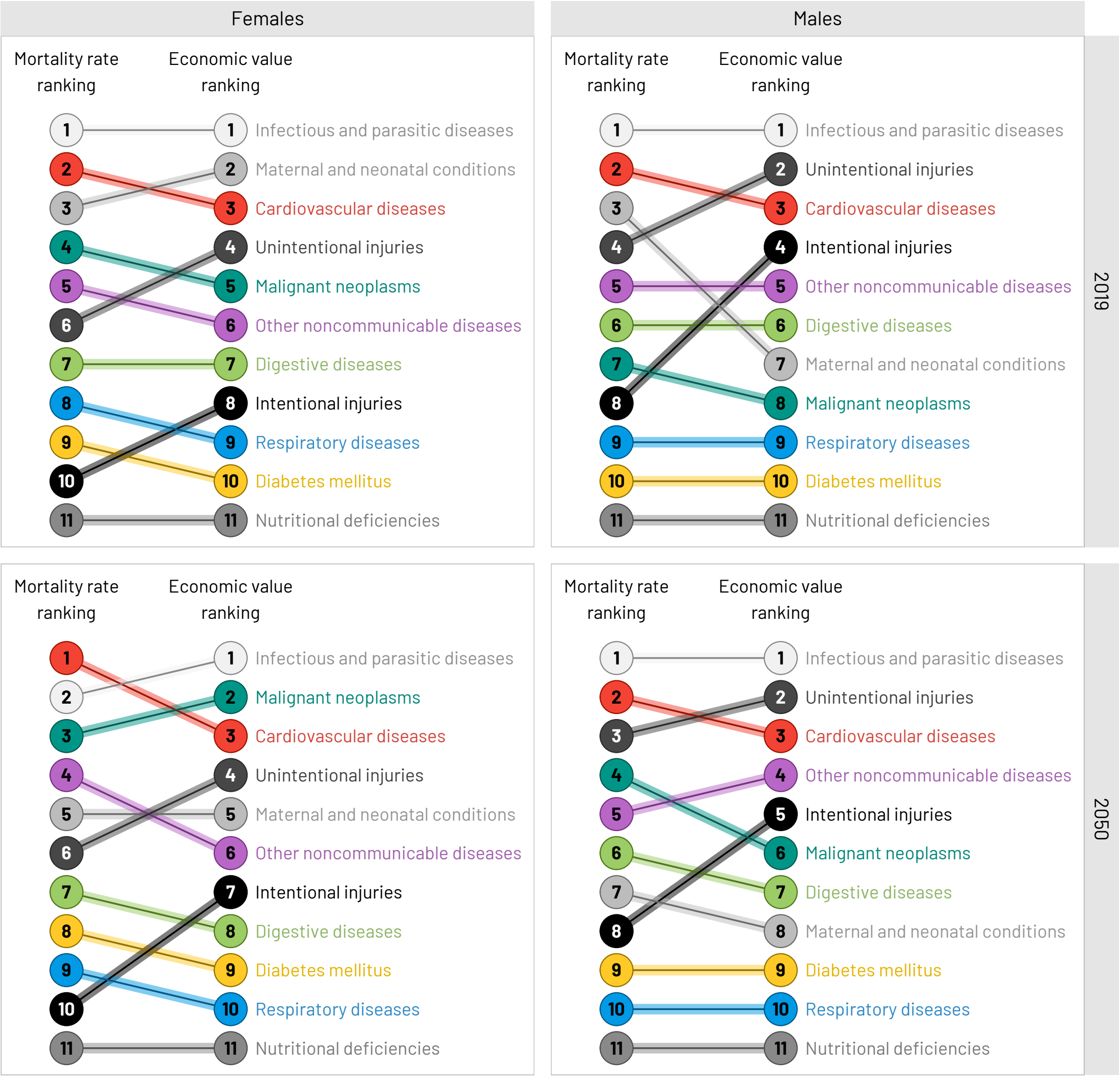

(G) World

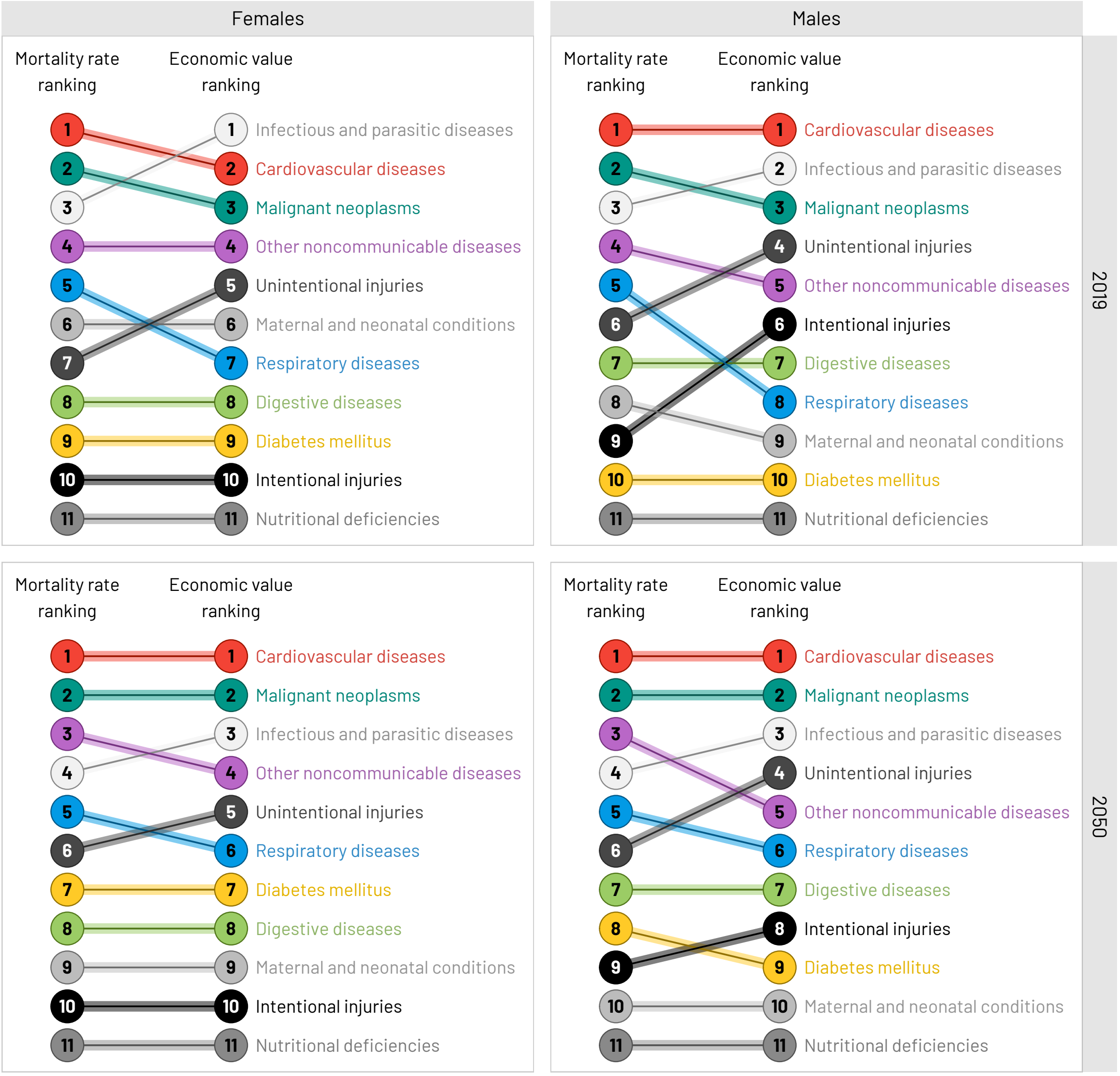
Females
Males
2019
2050
Mortality rate ranking
Economic value ranking
Infectious and parasitic diseases
Cardiovascular diseases
Malignant neoplasms
Other noncommunicable diseases
Unintentional injuries
Maternal and neonatal conditions
Respiratory diseases
Digestive diseases
Diabetes mellitus
Intentional injuries
Nutritional deficiencies
